# Supplementary material for: Modeling the detection range of pulsed calls from resident killer whale in nearshore waters of British Columbia, Canada
Source: PLoS One. 2025 Sep 26;20(9):e0331942. doi: 10.1371/journal.pone.0331942 (PMC12469096; doi:10.1371/journal.pone.0331942)
Supplement: S1 Table — (DOCX) [file pone.0331942.s001.docx]

| **Model Site** | **End point** | | | |
| --- | --- | --- | --- | --- |
|  | **Latitude(°)** | **Longitude(°)** | **Easting** | **Northing** |
| **East Point** | 48.862 | −122.769 | 516973.2 | 5412162.1 |
|  | 48.694 | −122.704 | 521815.9 | 5393503.5 |
|  | 48.704 | −123.065 | 495197.0 | 5394584.2 |
|  | 48.675 | −123.305 | 477514.4 | 5391402.2 |
| **Enterprise Reef** | 48.911 | −123.495 | 463738.7 | 5417653.7 |
|  | 48.856 | −123.430 | 468489.2 | 5411510.8 |
|  | 48.678 | −123.336 | 475287.4 | 5391771.5 |
|  | 48.821 | −123.323 | 476270.3 | 5407661.2 |
|  | 48.860 | −123.350 | 474304.6 | 5412002.2 |
| **Mouat Point** | 48.860 | −123.350 | 474304.6 | 5412002.2 |
|  | 48.835 | −123.451 | 466926.8 | 5409236.5 |
|  | 48.683 | −123.522 | 461571.8 | 5392325.7 |
|  | 48.623 | −123.384 | 471696.7 | 5385586.1 |
|  | 48.626 | −123.199 | 485341.0 | 5385916.7 |
| **Port Renfrew** | 48.589 | −124.741 | 371613.5 | 5383178.1 |
|  | 48.465 | −124.831 | 364632.4 | 5369616.4 |
|  | 48.341 | −124.463 | 391584.0 | 5355253.7 |
|  | 48.438 | −124.233 | 408779.9 | 5365684.4 |
|  | 48.531 | −124.461 | 392161.2 | 5376400.1 |
| **Sheringham Point** | 48.438 | −124.233 | 408779.9 | 5365684.4 |
|  | 48.257 | −124.242 | 407797.0 | 5345618.5 |
|  | 48.174 | −123.709 | 447275.6 | 5335870.7 |
|  | 48.170 | −123.963 | 428437.3 | 5335625.0 |
|  | 48.309 | −123.651 | 451698.5 | 5350859.5 |
| **Sturdies Bay** | 49.082 | −123.312 | 477253.2 | 5436655.9 |
|  | 49.005 | −123.145 | 489375.2 | 5427973.9 |
|  | 48.891 | −122.842 | 511571.7 | 5415319.4 |
|  | 48.858 | −123.008 | 499420.7 | 5411683.4 |
|  | 48.853 | −123.305 | 477610.1 | 5411164.7 |
| **Swiftsure Bank** | 48.589 | −124.741 | 371613.5 | 5383178.1 |
|  | 48.393 | −124.747 | 370661.3 | 5361500.0 |
|  | 48.693 | −124.984 | 354007.3 | 5395206.3 |
|  | 48.620 | −125.187 | 338832.8 | 5387487.7 |
|  | 48.433 | −125.207 | 336783.3 | 5366782.4 |
|  | 48.343 | −124.969 | 354077.4 | 5356300.0 |
| **Tilly Point** | 48.740 | −123.312 | 477068.7 | 5398614.5 |
|  | 48.629 | −123.291 | 478562.8 | 5386267.1 |
|  | 48.704 | −123.065 | 495197.0 | 5394584.2 |
|  | 48.582 | −123.024 | 498261.2 | 5381014.2 |
|  | 48.749 | −122.944 | 504128.9 | 5399501.9 |
