# Supplementary material for: Modeling the detection range of pulsed calls from resident killer whale in nearshore waters of British Columbia, Canada
Source: PLoS One. 2025 Sep 26;20(9):e0331942. doi: 10.1371/journal.pone.0331942 (PMC12469096; doi:10.1371/journal.pone.0331942)
Supplement: S3 Table — (DOCX) [file pone.0331942.s003.docx]

| **Model Site** | **Season** | **Time period selected** | **Origin of recordings** | **Number of 1‑minute measurements** | **Latitude (°N)** | **Longitude (°W)** | **Hydrophone depth (m)** |
| --- | --- | --- | --- | --- | --- | --- | --- |
| **East Point** | Summer | 2015 Jul 20 to 2015 Aug 19 | WTN Hydrophone | 43120 | 48.781 | -123.049 | 15 |
|  | Winter | 2018 Dec 13 to 2019 Feb 07 | VFPA-TC AMAR^a^ | 81640 | 48.760 | -123.063 | 15 |
| **Enterprise Reef** | Summer | 2016 Apr 29 to 2016 May 22 | WTN Hydrophone | 31580 | 48.847 | -123.348 | 18 |
|  | Winter | 2016 Mar 02 to 2016 Mar 10 | WTN Hydrophone | 11250 | 48.847 | -123.348 | 18 |
| **Mouat Point** | Summer | 2016 May 01 to 2016 Jun 08 | JASCO AMAR^b^ | 26160 | 48.774 | -123.343 | 15 |
|  | Winter | 2018 Jan 08 to 2018 Feb 09 | WTN Hydrophone | 45915 | 48.777 | -123.319 | 15 |
| **Port Renfrew** | Summer | 2019 Jul 01 to 2019 Aug 01 | DFO AMAR^c^ | 44415 | 48.505 | -124.517 | 171 |
|  | Winter | 2019 Jan 01 to 2019 Feb 01 | DFO AMAR^c^ | 44387 | 48.505 | -124.517 | 171 |
| **Sheringham Point** | Summer | 2015 May 29 to 2015 Jul 19 | WTN hydrophone | 73631 | 48.376 | -123.921 | 14 |
|  | Winter | 2014 Dec 17 to 2014 Dec 26 | WTN hydrophone | 11978 | 48.376 | -123.921 | 14 |
| **Sturdies Bay** | Summer | 2015 Sep 25 to 2015 Oct 27 | WTN hydrophone | 46379 | 48.876 | -123.309 | 11 |
|  | Winter | 2016 Feb 28 to 2016 Apr 25 | WTN hydrophone | 9900 | 48.876 | -123.309 | 11 |
| **Swiftsure Bank** | Summer | 2019 Jun 01 to 2019 Jul 01 | DFO AMAR^c^ | 43208 | 48.515 | -124.936 | 74 |
|  | Winter | 2019 Jan 01 to 2019 Feb 01 | DFO AMAR^c^ | 44416 | 48.515 | -124.936 | 74 |
| **Tilly Point** | Summer | 2017 Jun 20 to 2017 Jul 12 | WTN Hydrophone | 31038 | 48.732 | -123.205 | 16 |
|  | Winter | 2018 Jan 03 to 2018 Feb 09 | WTN Hydrophone | 48745 | 48.732 | -123.205 | 16 |

^a^Data used with permission from the Vancouver Fraser Port Authority and Transport Canada

^b^Data provided by JASCO Applied Sciences

^c^Data used with permission by Dr. Svein Vagle (DFO)
