# Supplementary figures and images for: Modeling the detection range of pulsed calls from resident killer whale in nearshore waters of British Columbia, Canada
Source: PLoS One. 2025 Sep 26;20(9):e0331942. doi: 10.1371/journal.pone.0331942 (PMC12469096; doi:10.1371/journal.pone.0331942)

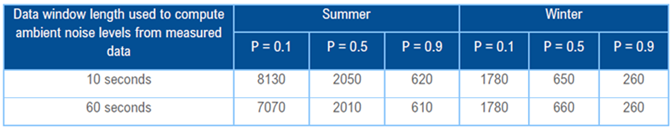

Supplement: S4 Table — (TIF) [file pone.0331942.s004.tif]

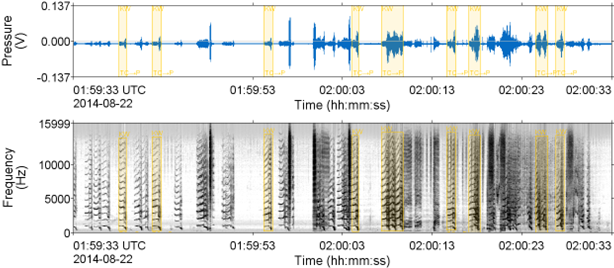

Supplement: S1 Fig — Yellow boxes indicate annotations used to determine the frequency distributions of the calls. (PNG) [file pone.0331942.s005.png]

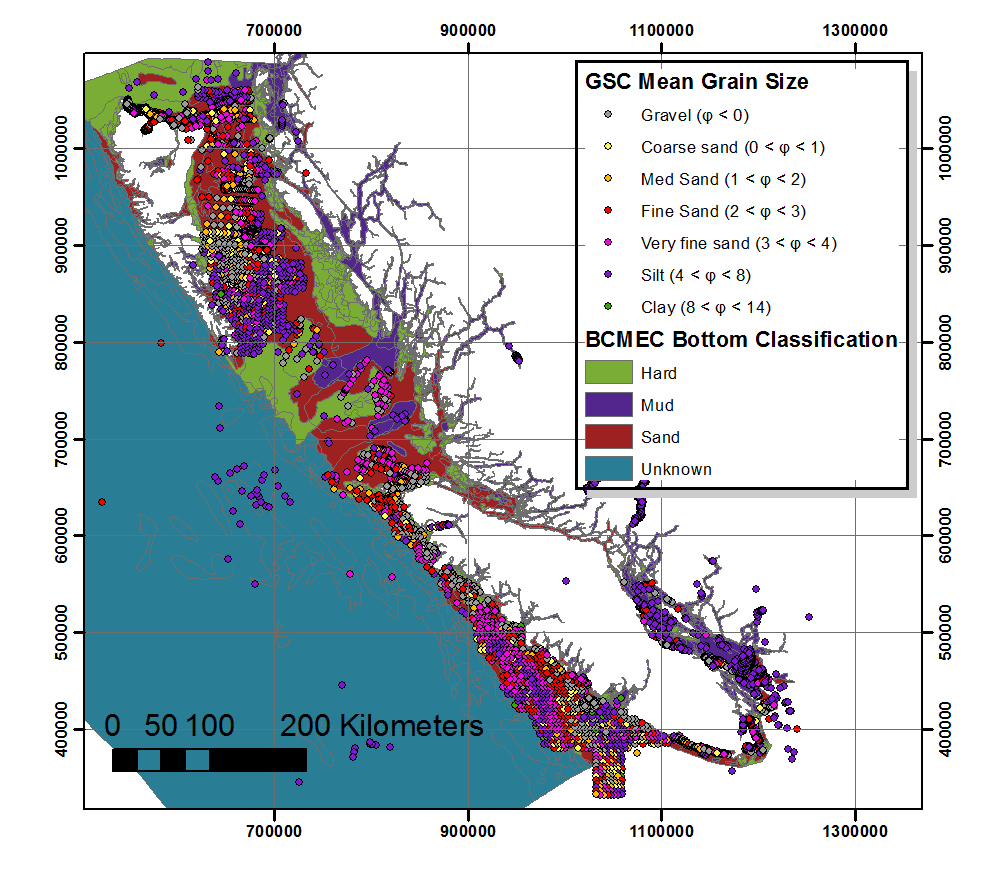

Supplement: S2 Fig — (TIF) [file pone.0331942.s006.tif]

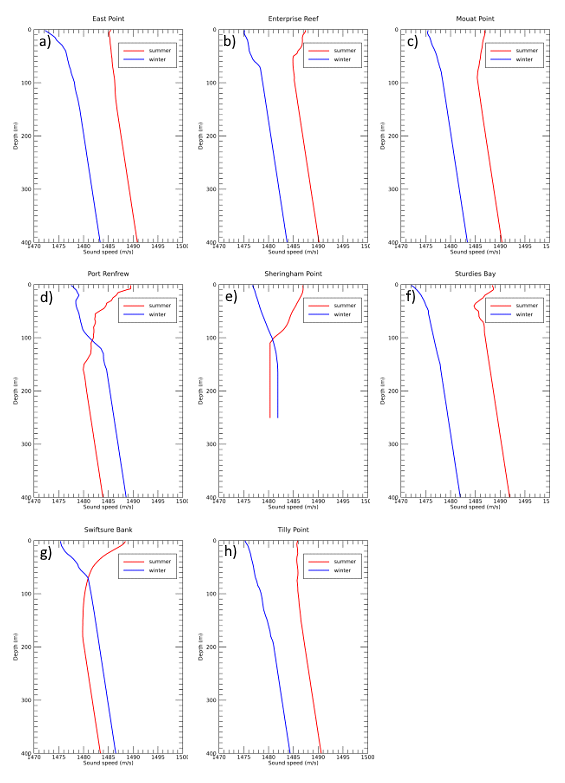

Supplement: S3 Fig — (TIF) [file pone.0331942.s007.tif]

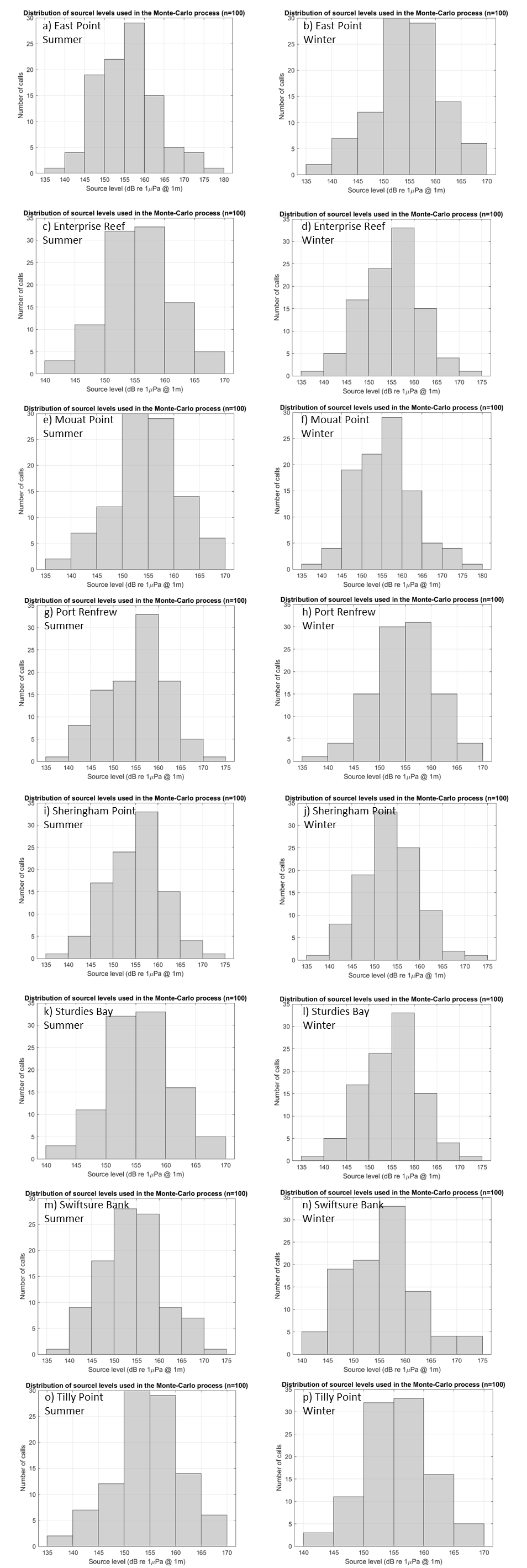

Supplement: S4 Fig — For (left) summer and (right) winter background conditions at (top to bottom) East Point, Enterprise Reef, Mouat Point, Port Renfrew, Sheringham Point, Sturdies Bay, Swiftsure Bank, and Tilly Point. (TIF) [file pone.0331942.s008.tif]

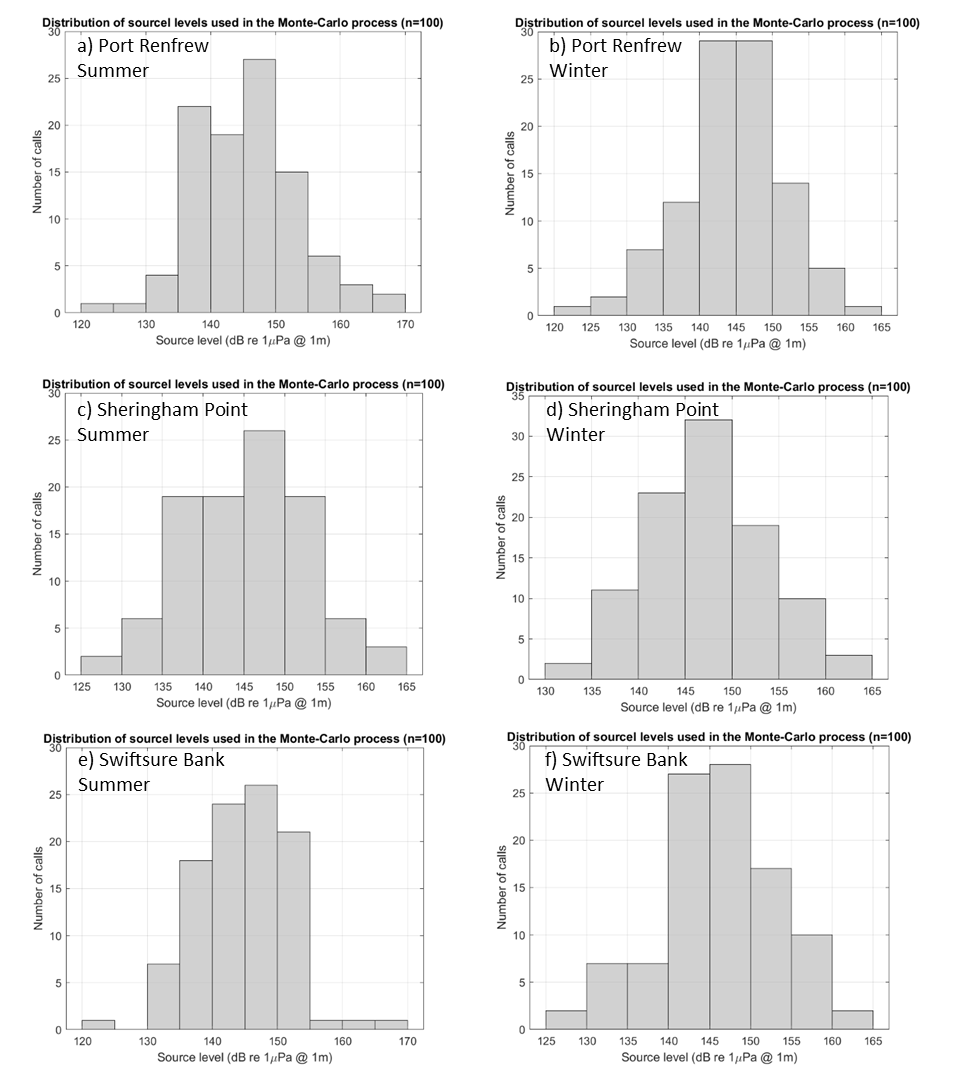

Supplement: S5 Fig — For (left) summer and (right) winter background conditions at (top to bottom) Port Renfrew, Sheringham Point, and Swiftsure Bank. (TIF) [file pone.0331942.s009.tif]

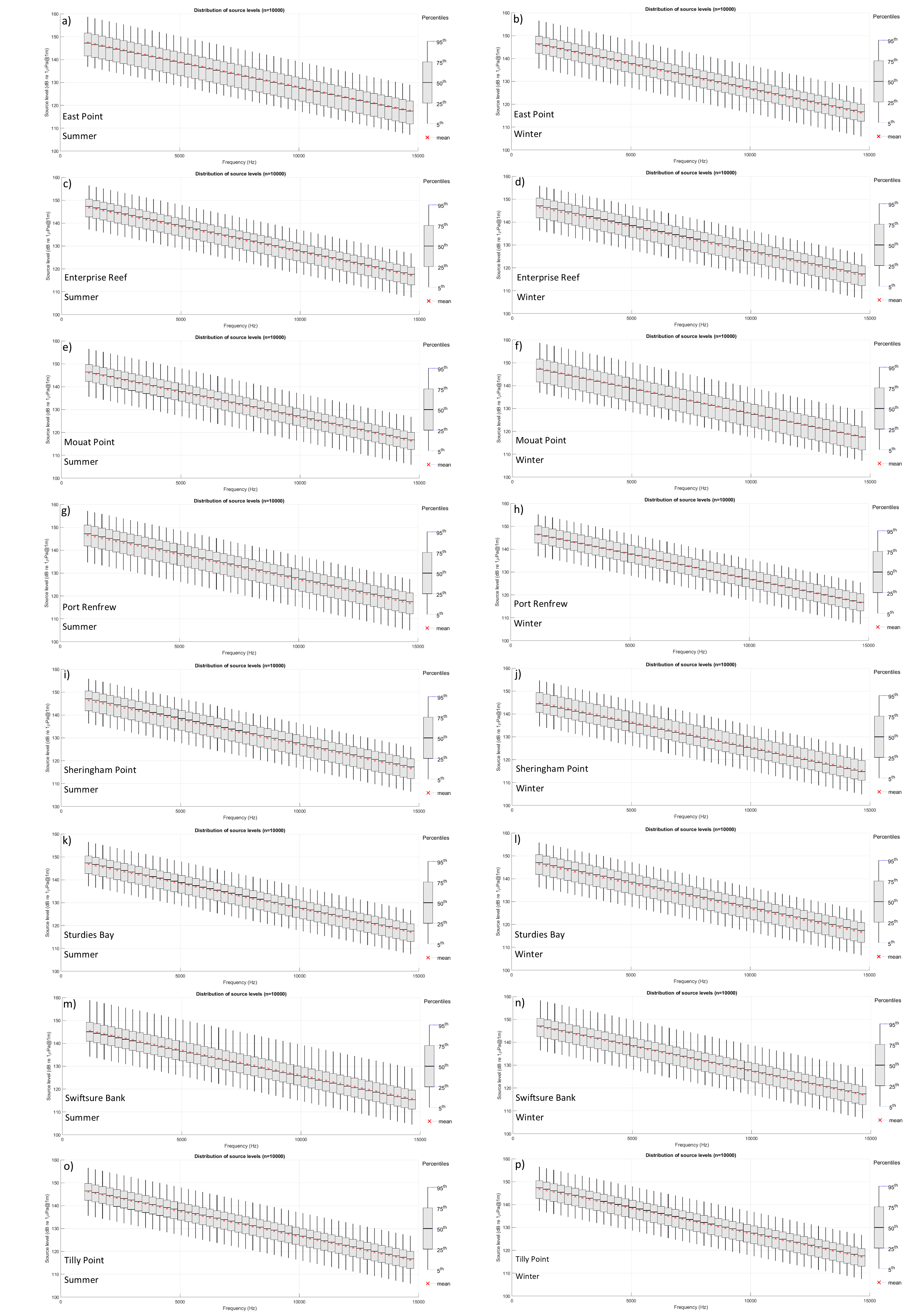

Supplement: S6 Fig — For (left) summer and (right) winter background conditions at (top to bottom) East Point, Enterprise Reef, Mouat Point, Port Renfrew, Sheringham Point, Sturdies Bay, Swiftsure Bank, and Tilly Point. (TIF) [file pone.0331942.s010.tif]

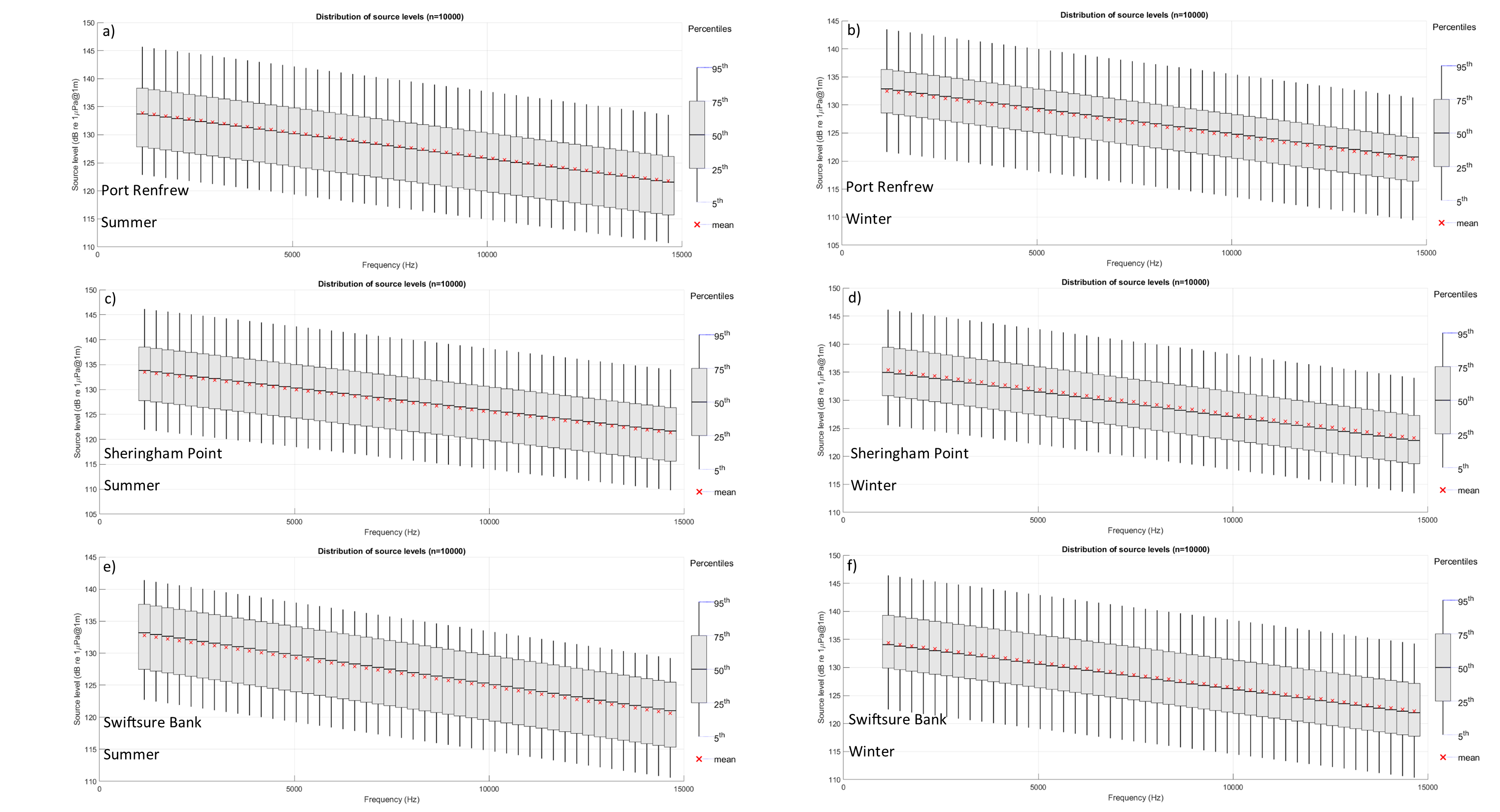

Supplement: S7 Fig — For (left) summer and (right) winter background conditions at (top to bottom) Port Renfrew, Sheringham Point, and Swiftsure Bank. (TIF) [file pone.0331942.s011.tif]

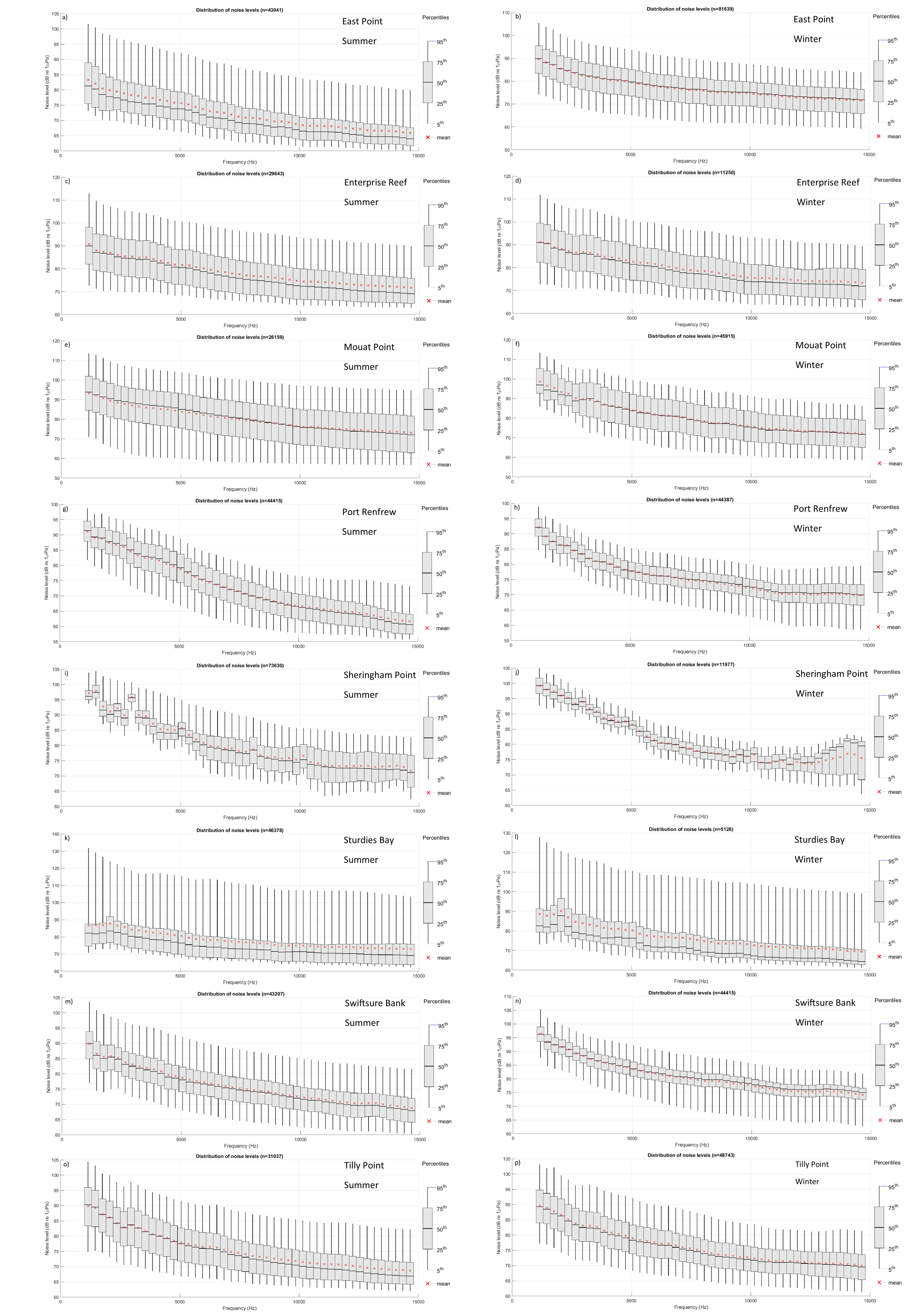

Supplement: S10 Fig — For (left) summer and (right) winter background conditions at (top to bottom) East Point, Enterprise Reef, Mouat Point, Port Renfrew, Sheringham Point, Sturdies Bay, Swiftsure Bank, and Tilly Point. (TIF) [file pone.0331942.s014.tif]

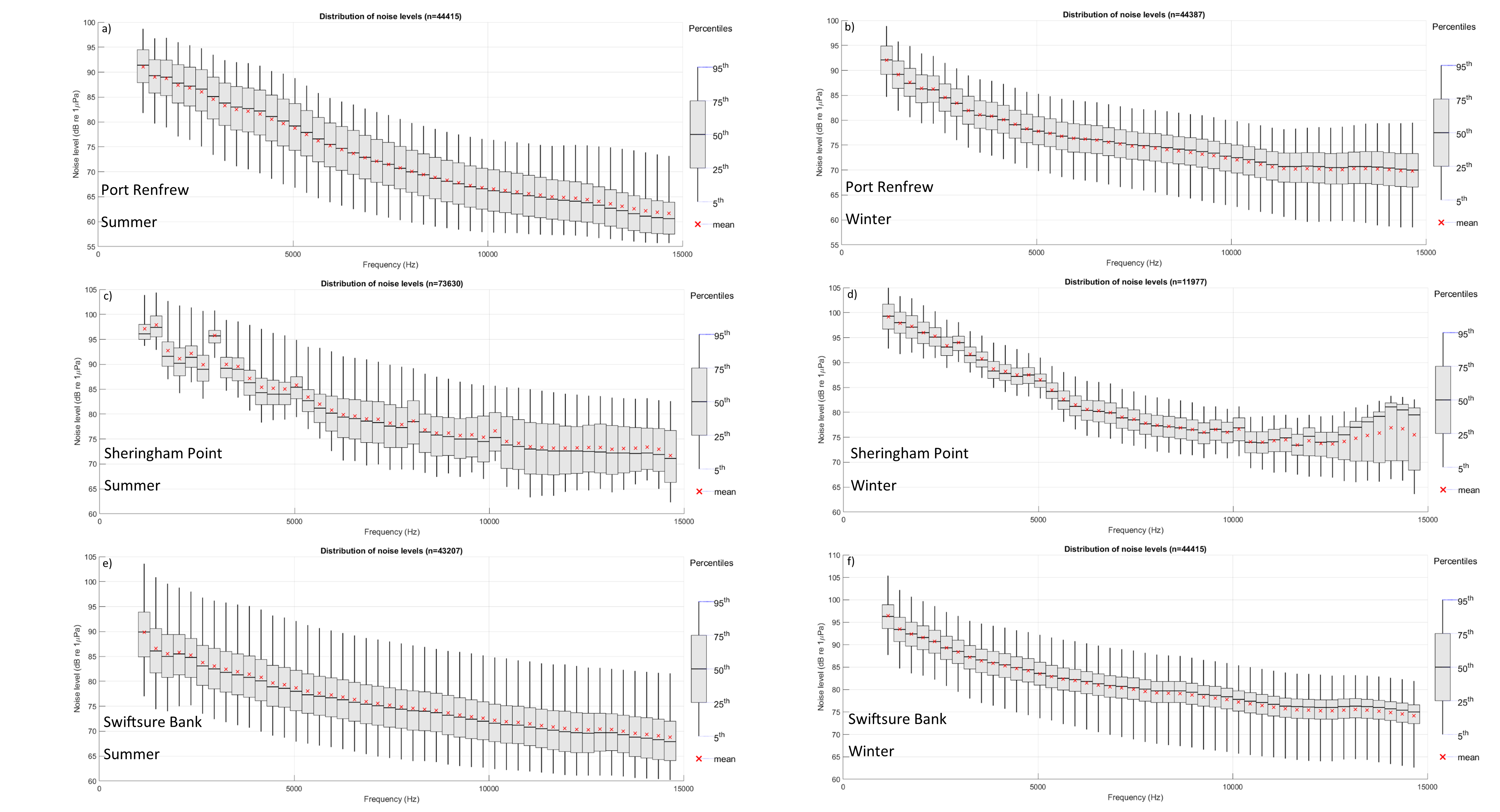

Supplement: S11 Fig — For (left) summer and (right) winter background conditions at (top to bottom) Port Renfrew, Sheringham Point, and Swiftsure Bank. (TIF) [file pone.0331942.s015.tif]

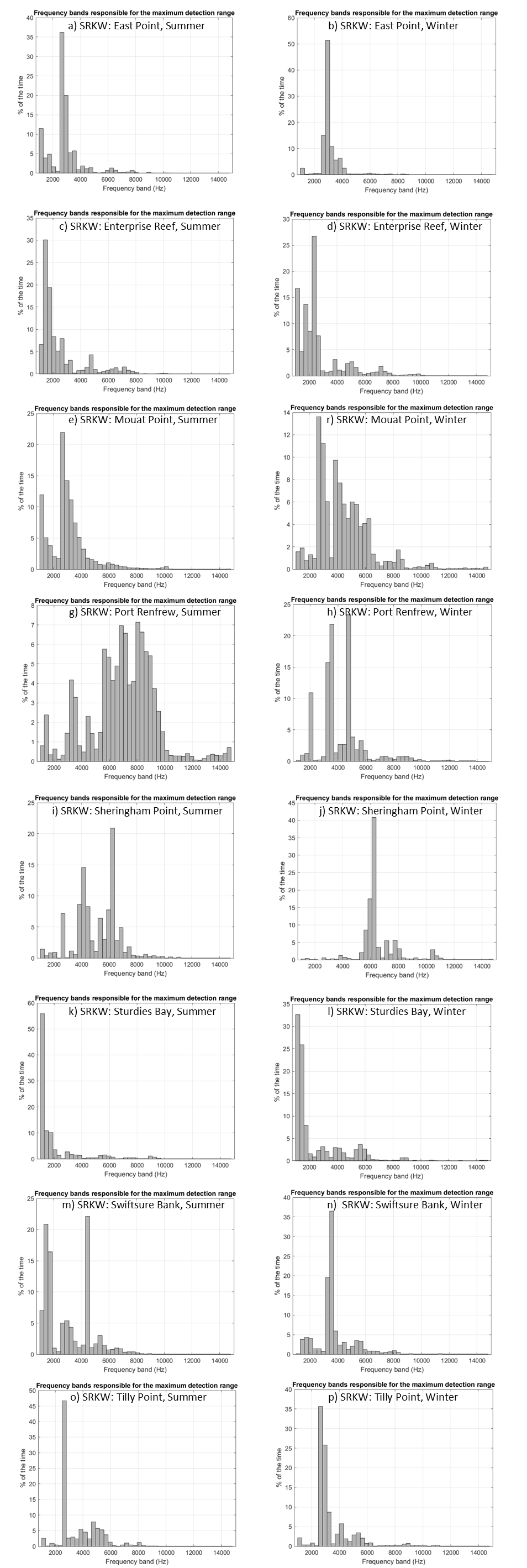

Supplement: S12 Fig — For (left) summer and (right) winter background conditions at (top to bottom) East Point, Enterprise Reef, Mouat Point, Port Renfrew, Sheringham Point, Sturdies Bay, Swiftsure Bank, and Tilly Point. (TIF) [file pone.0331942.s016.tif]

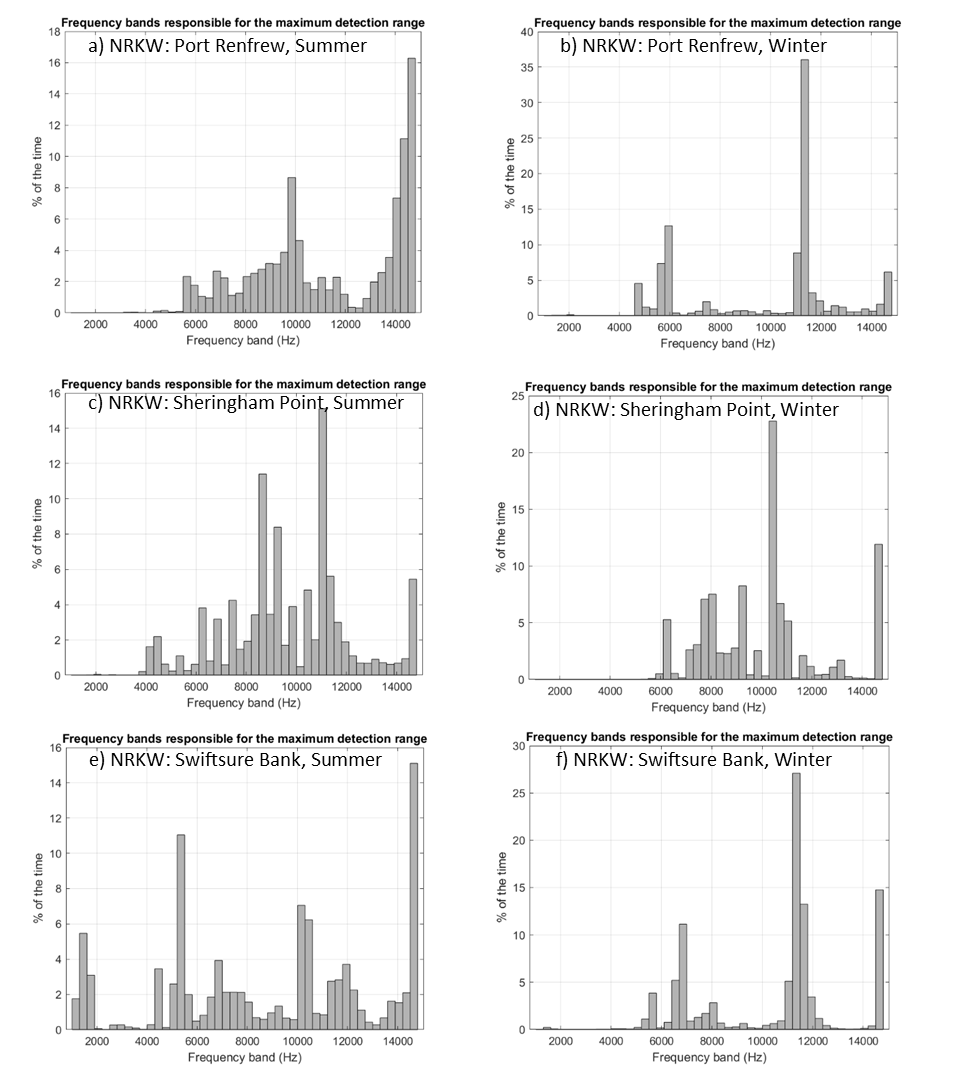

Supplement: S13 Fig — For (left) summer and (right) winter background conditions at (top to bottom) Port Renfrew, Sheringham Point, and Swiftsure Bank. (TIF) [file pone.0331942.s017.tif]

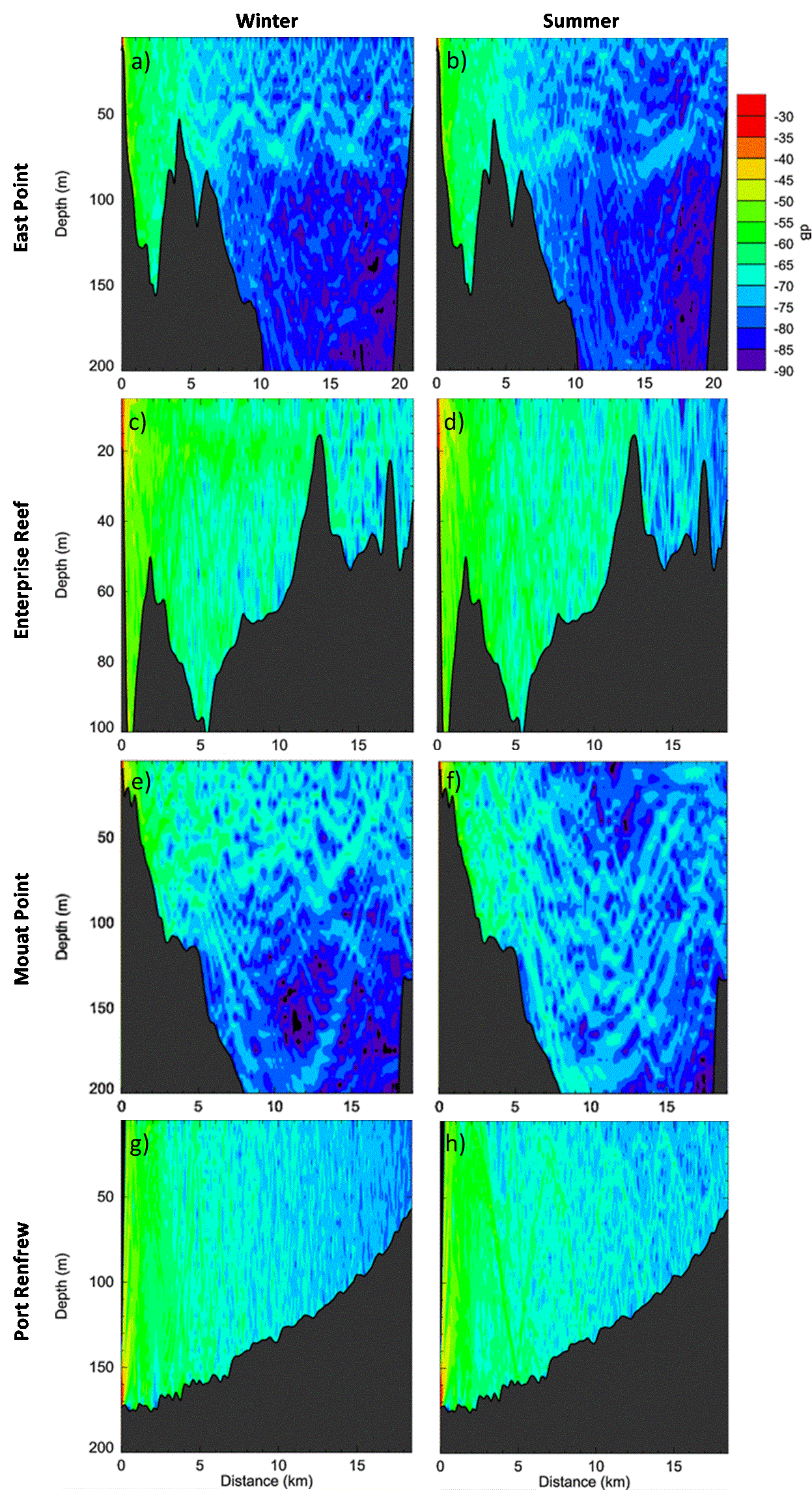

Supplement: S14 Fig — (TIF) [file pone.0331942.s018.tif]

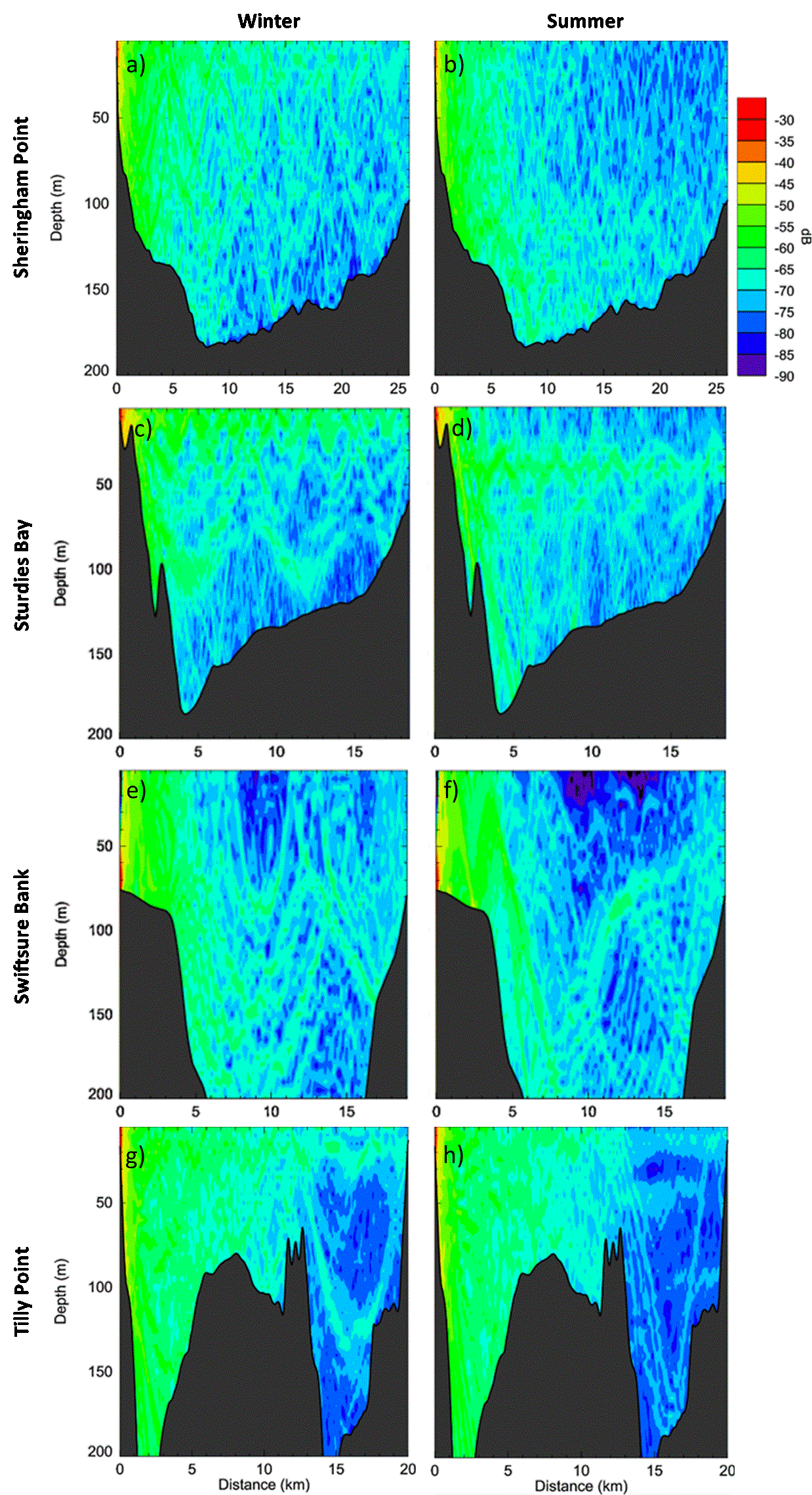

Supplement: S15 Fig — (TIF) [file pone.0331942.s019.tif]

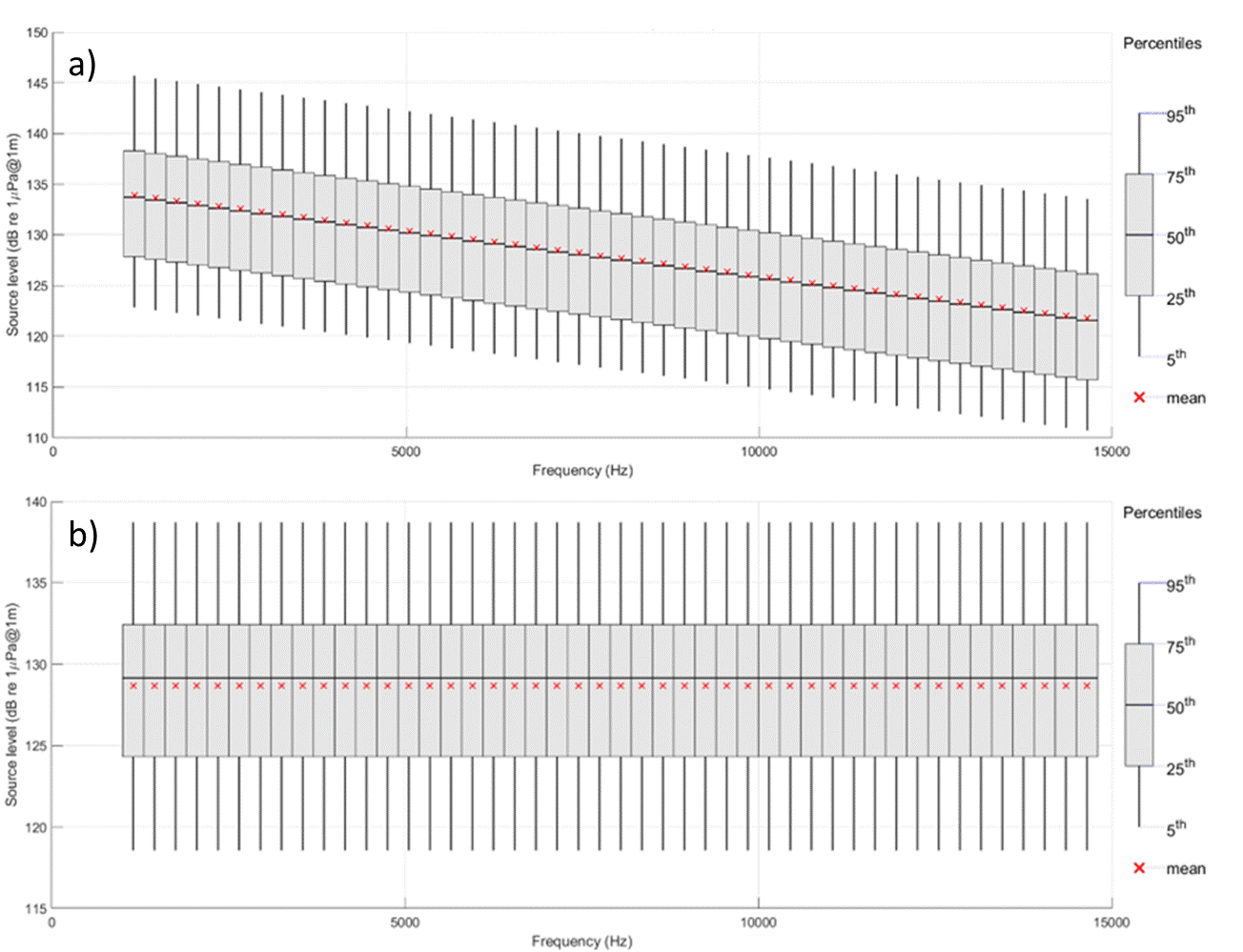

Supplement: S16 Fig — Statistical distribution of source levels (in 300 Hz bands) from the Monte Carlo simulation, as a function of frequency. (TIF) [file pone.0331942.s020.tif]

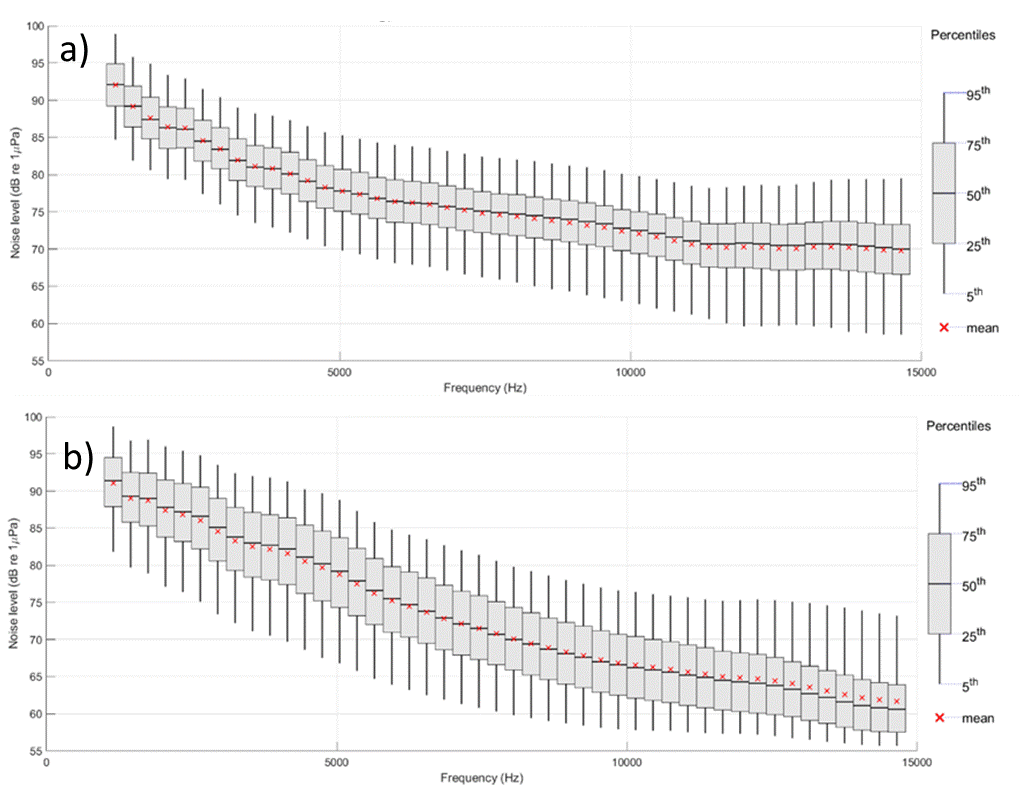

Supplement: S17 Fig — Percentile distribution of background sound levels (in 300 Hz bands). (TIF) [file pone.0331942.s021.tif]

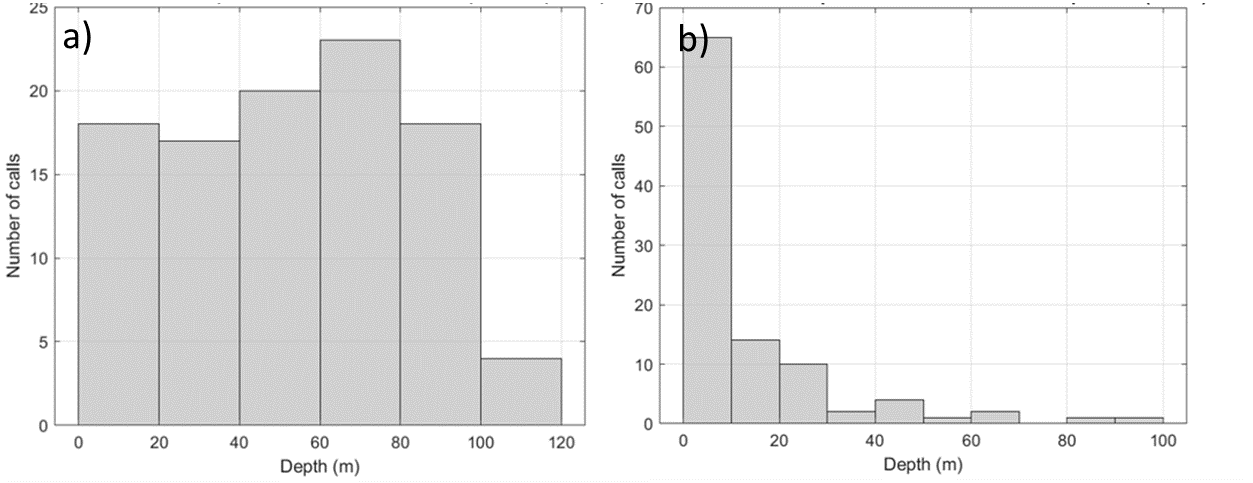

Supplement: S18 Fig — (TIF) [file pone.0331942.s022.tif]
