# Supplementary material for: Modeling the detection range of pulsed calls from resident killer whale in nearshore waters of British Columbia, Canada
Source: PLoS One. 2025 Sep 26;20(9):e0331942. doi: 10.1371/journal.pone.0331942 (PMC12469096; doi:10.1371/journal.pone.0331942)
Supplement: Attachment — Submitted filename: PONE-D-24-60689_reviewer_ed.pdf [file pone.0331942.s023.pdf]

PLOS ONE

Modeling the detection range of pulsed calls from resident killer whale in nearshore waters of British Columbia, Canada.

--Manuscript Draft--

**Manuscript Number:**	PONE-D-24-60689
**Article Type:**	Research Article
**Full Title:**	Modeling the detection range of pulsed calls from resident killer whale in nearshore waters of British Columbia, Canada.
**Short Title:**	Modeling the detection range of pulsed calls from resident killer whale
**Corresponding Author:**	Xavier Mouy<br>JASCO Applied Sciences (Canada) Ltd<br>Woods Hole, Massachusetts UNITED STATES OF AMERICA
**Keywords:**	passive acoustic monitoring; Salish Sea, Monte Carlo simulations
**Abstract:**	Passive underwater listening stations are often used to monitor the presence, distribution and movements of marine mammals. This requires an understanding of the distances at which marine mammal sounds can be detected at a location and at different times given varying ambient sound conditions. Here we describe a Monte Carlo approach for determining call detection probabilities as a function of distance for networked underwater listening stations deployed by Fisheries and Oceans Canada in the Salish Sea to track endangered Southern and Northern Resident Killer Whales. In our approach, we used ambient sound levels measured in situ, modelled propagation losses determined by an acoustic propagation model, and applied Monte Carlo simulations to capture the variability in call source levels and animal depths. Given that only some parts of the call frequency spectrum may be responsible for the maximum detection range, the analysis was carried out independently for consecutive 300 Hz frequency bands. Median detection range estimates ranged from 700 m at the noisiest time and location to 8 km at the quietest. A sensitivity analysis revealed that the frequency distribution of source levels used for the analysis was a major factor affecting median detection range results, while differences in propagation losses between summer and winter were less important.
**Order of Authors:**	Xavier Mouy
	Melanie Austin
	Jennifer Wladichuk
	Harald Yurk
**Additional Information:**	
**Question**	**Response**
**Financial Disclosure**<br><br>Enter a financial disclosure statement that describes the sources of funding for the work included in this submission. Review the submission guidelines for detailed requirements. View published research articles from *PLOS ONE* for specific examples.<br><br>This statement is required for submission and **will appear in the published article** if the submission is accepted. Please make	Yes

Powered by Editorial Manager® and ProduXion Manager® from Aries Systems Corporation

sure it is accurate.<br><br>**Funded studies**<br>Enter a statement with the following details:<br>• Initials of the authors who received each award<br>• Grant numbers awarded to each author<br>• The full name of each funder<br>• URL of each funder website<br>• Did the sponsors or funders play any role in the study design, data collection and analysis, decision to publish, or preparation of the manuscript?<br><br>Did you receive funding for this work?	
Please add funding details.<br>as follow-up to "**Financial Disclosure**<br><br>Enter a financial disclosure statement that describes the sources of funding for the work included in this submission. Review the submission guidelines for detailed requirements. View published research articles from *PLOS ONE* for specific examples.<br><br>This statement is required for submission and **will appear in the published article** if the submission is accepted. Please make sure it is accurate.<br><br>**Funded studies**<br>Enter a statement with the following details:<br>• Initials of the authors who received each award<br>• Grant numbers awarded to each author<br>• The full name of each funder<br>• URL of each funder website<br>• Did the sponsors or funders play any role in the study design, data collection and analysis, decision to publish, or preparation of the manuscript?<br><br>Did you receive funding for this work?"	This study was funded by Fisheries and Oceans Canada and JASCO Applied Sciences. XM also received support from the Investment in Science Fund at the Woods Hole Oceanographic Institution.
Please select the country of your main research funder (please select carefully	CANADA - CA

as in some cases this is used in fee calculation).<br>as follow-up to "**Financial Disclosure**<br><br>Enter a financial disclosure statement that describes the sources of funding for the work included in this submission. Review the submission guidelines for detailed requirements. View published research articles from *PLOS ONE* for specific examples.<br><br>This statement is required for submission and **will appear in the published article** if the submission is accepted. Please make sure it is accurate.<br><br>**Funded studies**<br>Enter a statement with the following details:<br>• Initials of the authors who received each award<br>• Grant numbers awarded to each author<br>• The full name of each funder<br>• URL of each funder website<br>• Did the sponsors or funders play any role in the study design, data collection and analysis, decision to publish, or preparation of the manuscript?<br><br>Did you receive funding for this work?"	
**Competing Interests**<br><br>Use the instructions below to enter a competing interest statement for this submission. On behalf of all authors, disclose any competing interests that could be perceived to bias this work—acknowledging all financial support and any other relevant financial or non-financial competing interests.<br><br>This statement is required for submission and **will appear in the published article** if the submission is accepted. Please make sure it is accurate and that any funding sources listed in your Funding Information later in the submission form are also	The authors have declared that no competing interests exist.

declared in your Financial Disclosure statement.<br><br>View published research articles from *PLOS ONE* for specific examples.<br><br>**NO authors have competing interests**<br><br>Enter: *The authors have declared that no competing interests exist.*<br><br>**Authors with competing interests**<br><br>Enter competing interest details beginning with this statement:<br><br>*I have read the journal's policy and the authors of this manuscript have the following competing interests: [insert competing interests here]*<br><br>* typeset	
**Ethics Statement**<br><br>Enter an ethics statement for this submission. This statement is required if the study involved:<br><br>• Human participants<br>• Human specimens or tissue<br>• Vertebrate animals or cephalopods<br>• Vertebrate embryos or tissues<br>• Field research<br><br>Write "N/A" if the submission does not require an ethics statement.<br><br>General guidance is provided below. Consult the submission guidelines for detailed instructions. **Make sure that all information entered here is included in the Methods section of the manuscript.**	N/A

**Format for specific study types**<br><br>**Human Subject Research (involving human participants and/or tissue)**<br>• Give the name of the institutional review board or ethics committee that approved the study<br>• Include the approval number and/or a statement indicating approval of this research<br>• Indicate the form of consent obtained (written/oral) or the reason that consent was not obtained (e.g. the data were analyzed anonymously)<br><br>**Animal Research (involving vertebrate animals, embryos or tissues)**<br>• Provide the name of the Institutional Animal Care and Use Committee (IACUC) or other relevant ethics board that reviewed the study protocol, and indicate whether they approved this research or granted a formal waiver of ethical approval<br>• Include an approval number if one was obtained<br>• If the study involved *non-human primates*, add *additional details* about animal welfare and steps taken to ameliorate suffering<br>• If anesthesia, euthanasia, or any kind of animal sacrifice is part of the study, include briefly which substances and/or methods were applied<br><br>**Field Research**<br><br>Include the following details if this study involves the collection of plant, animal, or other materials from a natural setting:<br>• Field permit number<br>• Name of the institution or relevant body that granted permission	
**Data Availability**<br><br>Authors are required to make all data underlying the findings described fully available, without restriction, and from the time of publication. PLOS allows rare exceptions to address legal and ethical concerns. See the PLOS Data Policy and FAQ for detailed information.	Yes - all data are fully available without restriction

Powered by Editorial Manager® and ProduXion Manager® from Aries Systems Corporation

A Data Availability Statement describing where the data can be found is required at submission. Your answers to this question constitute the Data Availability Statement and **will be published in the article**, if accepted.<br><br>**Important:** Stating 'data available on request from the author' is not sufficient. If your data are only available upon request, select 'No' for the first question and explain your exceptional situation in the text box.<br><br>Do the authors confirm that all data underlying the findings described in their manuscript are fully available without restriction?	
**Describe where the data may be found in full sentences. If you are copying our sample text, replace any instances of XXX with the appropriate details.**<br><br>• If the data are **held or will be held in a public repository**, include URLs, accession numbers or DOIs. If this information will only be available after acceptance, indicate this by ticking the box below. For example: *All XXX files are available from the XXX database (accession number(s) XXX, XXX.*).<br>• If the data are all contained **within the manuscript and/or Supporting Information files**, enter the following: *All relevant data are within the manuscript and its Supporting Information files.*<br>• If neither of these applies but you are able to provide **details of access elsewhere**, with or without limitations, please do so. For example:<br><br>*Data cannot be shared publicly because of [XXX]. Data are available from the XXX Institutional Data Access / Ethics Committee (contact via XXX) for researchers who meet the criteria for access to confidential data.*<br><br>*The data underlying the results presented in the study are available from (include the name of the third party*	All SPL and propagation loss coefficients files are available from the OSF repository located here:<br>https://osf.io/6ctjq/?view_only=f4e6e83b9eab427bb530a74638ba3e52

and contact information or URL).<br>• This text is appropriate if the data are owned by a third party and authors do not have permission to share the data.<br><br>* typeset	
Additional data availability information:	Tick here if the URLs/accession numbers/DOIs will be available only after acceptance of the manuscript for publication so that we can ensure their inclusion before publication.

Powered by Editorial Manager® and ProduXion Manager® from Aries Systems Corporation

Manuscript Click here to access/download;Manuscript;Manuscript.docx

Modeling the detection range of pulsed calls from *resident* killer whale in nearshore waters of British Columbia, Canada.

Xavier Mouy [1,2 * ‡], Melanie Austin [1 ‡], Jennifer Wladichuk [1], Harald Yurk [3]

[1] JASCO Applied Sciences, 2305-4464 Markham Street, Victoria, BC, Canada

[2] Currently at: Woods Hole Oceanographic Institution, Applied Ocean Physics and Engineering department, 266 Woods Hole Road, Woods Hole, MA, USA.

[3] Fisheries and Oceans Canada, Pacific Science Enterprise Centre, 4160 Marine Drive, West Vancouver, BC, Canada

[*] Corresponding author

[‡] Equal contribution

Email: xavier.mouy@whoi.edu

Abstract

Passive underwater listening stations are often used to monitor the presence, distribution and movements of marine mammals. This requires an understanding of the distances at which marine mammal sounds can be detected at a location and at different times given varying ambient sound conditions. Here we describe a Monte Carlo approach for determining call detection probabilities as a function of distance for networked underwater listening stations deployed by Fisheries and Oceans Canada in the Salish Sea to track endangered Southern and Northern Resident Killer Whales. In our approach, we used ambient sound levels measured *in situ*, modelled propagation losses determined by an acoustic propagation model, and applied Monte Carlo simulations to capture the variability in call source levels and animal depths. Given that only some parts of the call frequency spectrum may be responsible for the maximum detection range, the analysis was carried out independently for consecutive 300 Hz frequency bands. Median detection range estimates ranged from 700 m at the noisiest time and location to 8 km at the quietest. A sensitivity analysis revealed that the frequency distribution of source levels used for the analysis was a major factor affecting median detection range results, while differences in propagation losses between summer and winter were less important.

Introduction

An important question in conservation biology, beyond understanding the factors that determine the presence of animals in a particular environment, is how much time they spend within suitable habitats, and how they move within and between them. Addressing these questions can improve our understanding of habitat preferences, assess the impact of human disturbances, and guide the development of effective mitigation and avoidance strategies (1, 2). However, detecting and tracking animal movements in marine environments, particularly for highly mobile species such as whales and dolphins, remains a significant challenge (3). Reliable detection of whales and the timely transmission of this information to monitoring stations is crucial when the goal is to mitigate the adverse effects of human activities (4, 5). For instance, such detection is essential for alerting intervention teams to implement mitigation measures before whales enter high-risk areas, such as those affected by oil spills, areas surrounding loud sound-producing activities, or the path of a moving vessel.

Resident killer whales (*Orcinus orca*) in British Columbia, Canada, are primarily represented by two distinct populations: the Southern Resident Killer Whale (SRKW) and the Northern Resident Killer Whale (NRKW). Both populations are known for their foraging preference on Chinook salmon *(Oncorhynchus tshawytscha*) (7). The SRKW population, which primarily inhabits the waters off the coast of Washington State to the waters north of Vancouver Island is listed as endangered, with only 73 individuals remaining (8). The NRKW population, which primarily inhabits the waters off Washington State up to Glacier Bay in Southeast Alaska has been increasing in numbers since 1973 (est. 340-348 in 2022) but are still listed as threatened (9). Both populations inhabit the inshore waters of the Salish Sea, which ranges from Puget Sound in the South to the Northern sections of the Strait of Georgia to the western entrance of Juan de Fuca Strait (Figure 1). The Salish Sea is also characterized by high levels of human activity on the water, including both small and large commercial vessel traffic, commercial and recreational fishing, whale and wildlife watching, and recreational boating. It has a higher density of human settlements than any other coastal area of British Columbia (10). SRKW [illegible]aces significant threats from reduced prey availability, environmental contamination, and vessel-based acoustic and physical disturbances (11). The distribution of both populations, however, appears to be influenced by prey availability and environmental conditions (12-16). Effective monitoring and conservation efforts are crucial to support the recovery of these endangered whales (11, 17).

Passive Acoustic Monitoring (PAM) is a powerful tool to detect vocalizing cetaceans and has been used effectively to determine habitat presence, spatial and temporal use of habitats, and movements of animals (e.g., (5, 18-21)). Its effectiveness has substantially increased in the last few decades due to the development of digital acoustic recording devices that can collect, store, and transmit large amounts of acoustic data (20). This technological advancement has enabled continuous and long-term acoustic monitoring of underwater environments. Compared to other monitoring methods such as visual observations, PAM has several advantages including that it is less affected by daylight, weather, and sea conditions (36). However, it is only useful for monitoring animals that produce sounds (37). Killer whales are highly soniferous which makes them good candidates for PAM. They produce a variety of sounds including echolocation clicks during foraging and navigation, as well as narrowband whistles and broadband pulsed calls used in social communication. Discrete pulsed calls are the most commonly heard vocalization that occur in all behavioral contexts and can be distinguished aurally and sorted into distinct repertoires (23). Numerous studies have shown that PAM is an effective tool for enhancing monitoring efforts of killer whale habitat use, particularly during periods when visual observation is challenging, such as during the winter months (24-35).

As part of the Recovery Plan for Southern Resident Killer Whales (11), the Government of Canada's Department of Fisheries and Oceans (DFO) installed a network of PAM stations located in the inland waters around the Southern Gulf Islands and the south-west coast of Vancouver Island in British Columbia, Canada (Fig. 1). The goal of this network is to provide near-real-time detection and tracking of killer whales, specifically endangered Southern Resident Killer Whales (SRKW), and send alerts about potential threats to the whales, such as the possibility of physical disturbance from whale-vessel interactions and potential vessel strikes. To achieve near-real-time detection the acoustic data streams from these PAM stations undergo automated killer whale call detection procedures.

To properly interpret the results from PAM networks, it is important to estimate the distance over which the signals of interest can be detected. This detection range is influenced by the ambient sound in the environment, the sound propagation, and the properties of the sounds we want to detect. Because these factors are not static, the detection range can be highly dynamic and may vary among locations and seasons (39). Ambient sound fluctuates with weather conditions (e.g. wind, waves) and anthropogenic activities (e.g. vessel traffic). Sound propagation is influenced by environmental factors, such as bathymetry, water temperature, and pressure which affect the sound speed profile (38). And whale calls and acoustic behavior have evolved in these dynamic soundscapes which has led to adapted acoustic signaling. Commonly, propagation loss (PL) modelling is used to estimate detection ranges and parabolic PL models have been favored (41-43). In most cases in which PL modelling was applied, environmental conditions influencing the detectability of calls have been assumed somewhat constant and averages were used in the model assumptions. In more recent year's environmental conditions are seen as dynamic influences on the detectability of whale calls (44) and ambient sound variation has been considered an important influence on the detectability of calls (45). To improve the interpretability of PAM studies, it is important to recognize the association of influences such as ambient sound variation and the distribution of energy across the signal frequency spectrum on call detectability.

The objective of this study is to estimate the detection range of SRKW and NRKW calls at several locations of the DFO PAM stations over time and evaluate their ability to detect killer whales entering the Salish Sea. This effort focused solely on the detection of discrete pulsed calls since they occur in all behavioral contexts. To estimate the influence of the dynamic physical acoustic environment on call detectability, call detection range variation was modelled at eight PAM locations. A probabilistic modelling approach was applied using randomized inputs of call source levels and ambient sound levels over dominant frequency ranges to model detection range using modelled PL of the source level depending on the depth of calling whale

(randomly selected based on a killer whale depth distribution from tagging data) to predict received call levels at the eight PAM stations.

Fig 1. Map of the study area indicating locations in and adjacent to the Salish Sea in British Columbia, Canada where call detectability of killer whale calls was investigated.

Methods

Approach

The acoustic detection range of a killer whale call was estimated by calculating the distance from a bottom mounted hydrophone where the received sound level of a call remains more than a detection threshold (DT) above the ambient sound level in the same frequency band. The received sound level of a killer whale call (RL) is the difference between the sound level at the source (i.e., the whale) and the propagation loss between the whale and the hydrophone (46):

$$RL = SL - PL, \qquad (1)$$

where SL is the source level in dB re 1 μPa^{2}m^2 and PL is the propagation loss in dB re 1 m^2. We calculated PL using a specialized acoustic model that integrates parabolic equation and ray tracing methods (MONM; JASCO Applied Sciences, Ltd.) and accounts for source depth, receiver depth, and environmental and bathymetric conditions between the source and receiver. For this analysis, we obtained killer whale call source levels from published and unpublished literature and ambient sound levels recorded at (or near) the model locations.

The maximum distance at which a call can be detected is where the call's received level, RL, exceeds the ambient sound level, NL, at the recorder in the same frequency band by at least the detection threshold, DT:

$$RL(f,z,r) \geq NL(f) + DT, \qquad (2)$$

DT was set to 5 dB for this analysis. The detection threshold used here strictly represents the signal processing detection threshold for automated detectors and is not related to the listening detection threshold of the animals. This approach assumes

detection using a single omni-directional hydrophone and does not account for processing gain that is possible using a hydrophone array.

At a given source depth, the detection range was estimated separately for each 300 Hz frequency band between 1000 and 14800 Hz (46 frequency bands total) and was calculated as:

$$R(f) = 10^{\frac{SL(f) - NL(f) - DT}{PL(f)}} \quad (3)$$

The final detection range was then defined as:

$$R_{max} = \arg max_f \left(R(f)\right) \quad (4)$$

The 300 Hz frequency bandwidth was selected, as we assume it is the smallest bandwidth necessary for an automated detector to detect killer whale pulsed calls. The frequency boundaries 1000 and 14800 Hz were chosen to cover the frequency range of killer whale pulsed calls (47, 48).

Ambient noise level, NL, varies over time due to sounds from passing ships, weather, and breaking wave sound, and often from non-acoustic water flow noise over the sensor caused by tidal currents. The ambient sound levels at the hydrophones vary substantially over time due to these influences. To simulate the actual conditions at each model site, ambient sound levels were calculated from acoustic recordings collected by the shore-cabled DFO hydrophones and DFO AMAR recorders (Autonomous Multichannel Acoustic Recorders, JASCO Applied Sciences) or by JASCO AMARs deployed near DFO hydrophones if actual DFO data were unavailable.

The detection range was calculated for each minute of the ambient sound data from the DFO or JASCO recordings; the probability of detecting a killer whale call at a given range was then taken to be the number of 1-minute recordings with a detection range equal to or greater than that range divided by the total number of 1-minute recordings.

To estimate the detection range of the killer whale pulsed calls, a Monte Carlo simulation was used to account for the measured variability in source levels and animal depths. Detection ranges were calculated 10,000 times for all ambient sound levels available by randomly choosing 100 normally distributed source level values, with means and standard deviations from the literature (see below), and 100 animal depths from a log-logistic distribution based on animal tagging data (see below). Each iteration of the Monte Carlo process provided a probability of detection for each range. The distribution of the 10,000 detection probabilities obtained at the end of the Monte Carlo simulation are represented for each range by the percentiles 25, 50, and

75. The detection range of SRKW discrete pulsed calls was estimated at eight PAM locations (Fig 1). The detection range of NRKW pulsed calls was also estimated for three locations (Port Renfrew, Sheringham Point, and Swiftsure Bank).

Source Level Estimation

Killer whales produce three primary types of sounds: clicks, whistles, and pulsed calls (Table 1). Clicks are short pulses that usually occur in a series. A click duration ranges from 0.1 to 25 ms (49). Whistles are single narrow-band tones in the 1.5–18 kHz frequency band. They have little harmonic structure. Their duration ranges from 50 ms to 12 s (49). Pulsed calls are harmonically structured with frequency ranges between 80 Hz and 40 kHz (50, 51). Pulsed calls were modelled in this analysis because they typically propagate better over greater distances than whistles and clicks and are used most often by the whales (49, 52). We used the source levels reported by Holt et al. (53) for SRKW (as they cover stereotyped calls from three different pods) and the source levels reported by Wladichuk et al. (54) for NRKW. The source levels for NRKW do represent those of calls produced by two acoustic clans (A and G clans (47)). There are three clans in the NRKW and clans show differences in habitat use. A-clan and G-clan have been reported to use areas monitored by two of the PAM stations for which detection range was modelled (Swiftsure Bank and Port Renfrew). Source levels reported in the literature are described by their mean and standard deviation. To include the variability of the source level values in the detection range analysis, 100 source level values were randomly selected during the Monte-Carlo process from a Gaussian distribution defined by the mean and standard deviation from Holt et al. (53) and Wladichuk et al. (54).

Table 1. Acoustic source levels of killer whale calls from relevant literature for different call types and populations of killer whales in this study.

Study Area	Population	Call type(s)	Source levels in dB re 1 µPa @ 1 m (mean ± SD)	Reference
**Johnstone Strait, BC**	Northern resident	Whistles	140.2 ± 4.1	Miller (50)
		Variable calls	146.6 ± 6.6	
		Stereotyped calls	152.6 ± 5.9	
**Johnstone Strait, BC**	Northern resident	Stereotyped calls	145.3 ± 6.8	Wladichuk et al. (54)
**San Juan Islands, WA**	Southern resident (J pod)	Stereotyped call S1	155.3 ± 7.4	Holt et al. (55)
**San Juan Islands, WA**	Southern resident (J, K, and L pods)	Stereotyped calls	155.1 ± 6.5	Holt et al. (53)

The distribution of the energy of killer whale calls across frequencies is an important parameter that can affect how far a call can be detected. Unfortunately, all reported source levels in the literature are broadband (i.e., a single value for the entire frequency band of the killer whale calls). To address this, we analyzed 35 SRKW pulsed calls and 14 NRKW pulsed calls with high Signal-to-Noise Ratio (SNR). The SRKW call data set was collected with bottom mounted hydrophones deployed by the DFO Cetacean Research Program, Ecosystem Science Division at various locations, and is composed of a selection of typical call types utilized by all SRKW pods, including types S1, S3, S4, S10, S13, S42, and S44 (56). The NRKW calls were collected by a hydrophone deployed off Northern Vancouver Island in the Johnstone Strait area in 2019 and provided by Dr. Jennifer Wladichuk (JASCO, University of Victoria). The calls reflect a subset of call types used by NRKW predominantly by members of two acoustic clans (A and G clan), (47)) but could be calls from either clan, as both were present during the measurements. The measurements, however, may not represent the full range of source levels within call repertories of both clans. Given the lack of information available in the literature, we assumed that the high SNR suggested that the vocalizing animals were very close to the hydrophone and that these calls could be used to estimate the distribution of energy across frequencies at the source.

The PAMlab software (JASCO Applied Sciences), was used to annotate the killer whale calls (Fig 2). A custom MATLAB script was then used to calculate the sound pressure level (SPL) in each 300 Hz frequency band (Fig 2 c-d) to match the minimum bandwidth that is accepted by the automated detector used to analyze data from the PAM stations. The script next performed a linear fit to the measured data (least-square fit) between 1000 and 14800 Hz to model the slope of the SPL across frequencies (Fig 2, red line). The slope of the fitted line was -0.66 dB per 300 Hz band for SRKW and -0.27 dB per 300 Hz band for NRKW calls. Source levels used in the Monte-Carlo process were all represented with this frequency distribution.

Fig 2. Frequency distribution of killer whale calls. Spectrogram of a selection of (a) southern resident killer whale pulsed call recordings and (b) northern resident killer whale pulsed call recordings used to determine the energy distribution of killer whale calls across frequency. Relative spectral distribution of (c) southern resident killer whale call source levels and (d) northern resident killer whale call source levels in 300 Hz bands across 1-15 kHz range based on 35 and 14 high SNR calls, respectively, recorded from small vessels.

Vocalization depth

Propagation loss is depth dependent, so the depth at which an animal vocalizes greatly impacts how far from the hydrophone its call can be detected. This was accounted for in the Monte Carlo process by repeating the detection range calculations for 100 source depths randomly selected from a distribution representing the typical depth distribution of SRKW, modelled from tag data collected by Brianna Wright (DFO – Cetacean Research Program). A log-logistic model, $f(z|\mu,\sigma)$, was fitted to the tag data using Maximum Likelihood:

$$f(z|\mu,\sigma) = \frac{e^{y}}{\sigma z(1+e^{y})^{2}}, \tag{5}$$

where $y = \frac{\log(z)-\mu}{\sigma}$, z is the animal's depth ($z \geq 0$), $\mu = 2.0212$, and $\sigma = 0.7739$. Because the hydrophones are located in water depths less than 200 m, the log-logistic model was created using all the depths from the tag data less than 200 m. Fig 3 shows the probability distribution of the empirical data collected by the tag (n = 2,215,700) and the probability distribution of 2,215,700 depth samples randomly drawn from the log-logistic model.

Fig 3. Dive depth probability distribution and distribution of samples drawn from the log-logistic model for the Monte Carlo simulation.

Propagation loss modelling

Detection ranges were modelled at each of the DFO PAM station sites, (Table 2 and Fig 4). For each location, propagation loss was modelled along transects in different directions (Fig 4, red lines. S1 Table), to sample the propagation loss characteristics at multiple depths as a function of range and azimuth from each location. JASCO's Marine Operations Noise Model (MONM) was used to calculate the propagation loss. MONM takes as input the source location and depth, a geoacoustic profile based on the sediment properties, a sound speed profile for the water column, and a record of the bathymetry along the modelled transects. MONM computes range-, depth-, and frequency-dependent propagation loss using either a split-step Padé solution (57) to the wave equation (used here for frequencies below 2.5 kHz) or a ray-tracing engine based on the Bellhop algorithm (58), which was used here for frequencies above 2.5 kHz. The model accounts for absorption, which can be important at the frequencies considered in this study. Calculations were computed at three frequencies within each 300 Hz band and averaged to provide a propagation loss estimate for each frequency band.

All resulting propagation loss values were plotted as a function of range and the data were fit with an equation of the form:

$$PL(f,z,R) = A(f,z) - n(f,z)logR \quad (6)$$

for frequency (f) in Hertz, depth (z) in meters, and range (R) in meters. The resulting PL values were used in the Monte Carlo simulation for the corresponding depths and frequency bands. For interpretation purposes, the parameter n in Eq 6 is representative of a geometric spreading loss term and it characterizes the amount by which the sound level decreases with the logarithm of distance away from the source for each modelled location.

Table 2. Model site coordinates).

Model site	Latitude (°)	Longitude (°)	Easting	Northing	Water Depth (m)
**East Point**	48.781	−123.049	496427.0	5403087.9	16
**Enterprise Reef**	48.847	−123.348	474502.9	5410499.6	19
**Mouat Point**	48.777	−123.319	476546.4	5402738.1	16
**Port Renfrew**	48.504	−124.517	387952.7	5373481.3	172
**Sheringham Point**	48.376	−123.921	431764.4	5358486.1	15
**Sturdies Bay**	48.876	−123.309	477306.3	5413706.9	12
**Swiftsure Bank**	48.515	−124.936	357034.3	5375393.4	75
**Tilly Point**	48.732	−123.205	484910.2	5397719.7	17

Fig 4. Map of the model sites with transects used for propagation loss computations. Colour-shaded bubbles group sites according to geoacoustic properties. Yellow circles denote locations of ambient sound measurements.

Five arc second ocean bathymetry data for the Salish Sea area were obtained from the high-resolution NOAA digital elevation model (59). These data were supplemented to the north and west with lower resolution soundings from a Canadian Hydrographic Service data set provided, with permission, by Nautical Data International Inc. Bathymetry data were re-projected onto a UTM Zone 10N coordinate grid with 100 × 100 m resolution, for use with MONM.

Sound propagation in shallow water is strongly influenced by the geoacoustic properties of the seafloor. These include the density, the compressional wave (P-wave) speed, the shear wave (S wave) speed, the compressional wave attenuation, and the shear wave attenuation of the seabed sediments. MONM incorporates these parameters when calculating propagation loss. Geoacoustic profiles for each model site were constructed to simulate the major features of the sediments. The geoacoustic properties for all the model sites were estimated based on the mean grain size from surface and sub-surface sediment grain-size samples from the Geological Survey of Canada (GSC) and the BC Marine Ecological Classification (BCMEC) maps (60).

Geoacoustic parameters were then computed based on Buckingham's grain-shearing model (61). The grain-shearing model computes geoacoustic properties of seabed sediment (sand, silt, clay) from porosity and grain size. Four geoacoustic profiles were created based on location and the geological data (S1 Fig, S2 Table). The colored bubbles in Fig 4 indicate which profile (A through D) was used at each model site.

Water column sound speed profiles for summer and winter were derived from data collected by DFO in 2018 and 2019 near each of the model sites. These were augmented, typically with deeper depth data, from the US Naval Oceanographic Office's Generalized Digital Environmental Model (GDEM) V 3.0 (62, 63). GDEM provides an ocean climatology of temperature and salinity for the world's oceans on a latitude-longitude grid with 0.25° resolution, with a temporal resolution of one month, based on global historical observations from the US Navy's Master Oceanographic Observational Data Set (MOODS). At each site, the CTD data were first extended to the deepest water depths (i.e., 400 m except 250 m for Sheringham Point). The temperature-salinity profiles were converted to sound speed profiles according to Coppens (64). The data were then smoothed using boxcar averaging and interpolated to get values at specified depths for modelling. If applicable, multiple profiles were averaged to compute the final profile. Winter and summer sound propagation conditions are very distinct (S2 Fig). In winter, cooler surface waters, wind-driven mixing, and atmospheric cooling result in a strong surface-duct sound profile. In summer, heating of the surface results in a downward refracting sound profile at most of the sites. Note that we did not use a full wave acoustic propagation model like in Helble et al, 2022 (43) but used MONM to model propagation losses on a wide frequency range, which in our case was more computationally efficient and scalable to multiple locations.

Ambient sound level characterization

Ambient sound levels were calculated from acoustic recordings collected by the DFO-PAM hydrophones or by Autonomous Multi-Channel Acoustic Recorders (AMARs; JASCO) deployed near PAM sites when the DFO data were unavailable (Fig 4 yellow circles; S3 Table). Ambient sound levels were obtained from approximately one month of acoustic data collected at each site and season (winter vs summer, S3 Table). The East Point winter data were collected by an AMAR deployed by JASCO and were used with permission from Vancouver Fraser Port Authority and Transport Canada. The acoustic data used for the Mouat Point site (summer) were collected by an AMAR and were provided for this analysis by JASCO Applied Sciences. Acoustic recordings at the Port Renfrew and Swiftsure Bank site were collected by AMARs and were provided by Dr. Svein Vagle (DFO). All other acoustic recordings were collected by the cabled icListenHF hydrophones (OceanSonics)

deployed by DFO. All acoustic data acquired with AMARs used an M36-V35-100 calibrated omnidirectional hydrophone (GeoSpectrum Technologies Inc.; −165 ± 5 dB re 1 V/µPa nominal sensitivity) and a gain of 0 dB.

Ambient sound processing was performed using the PAMlab software (JASCO Applied Sciences). The raw pressure waveform data were scaled according to the mean calibrated pressure sensitivity of the recorder and adjusted for the frequency response of the hydrophone sensor. Time domain pressure waveforms were analyzed to find SPL for each minute of data. SPL, which is a physical measure of sound amplitude, can be averaged over time and integrated over different frequency bands. For this study, SPL was calculated for each 300 Hz frequency band between 1000 and 14800 Hz (46 frequency bands in total). The frequency boundaries 1000 and 14800 Hz were chosen to cover the frequencies of killer whale pulsed calls (47, 48).

Results

The 100 randomly drawn broadband source levels from the Monte Carlo simulations for each model site approximate the normal distribution from which they were drawn (S3 Fig, S4 Fig). The broadband source level samples ranged in value from 135 to 170 dB re 1 µPa, and 120 to 165 dB re 1 µPa for SRKW and NRKW, respectively. Simulated source levels were maximum at low frequencies (S5 Fig and S6 Fig), as was the model used for the distribution that the band levels were drawn from. Distributions of vocalization depths did not exceed 100 m depth (S7 Fig and S8 Fig) and most of the simulated calls depths were within the top 20 m of the water column. Background sound levels at each model site were highest at 1000 Hz and decreased as frequency increased (S9 Fig and S10 Fig). The mean band levels were all below 100 dB re 1 µPa. At several sites, sound levels were higher in winter than in summer. The propagation loss modelling results indicate that sound levels decrease most rapidly with range at Sturdies Bay (the shallowest model site) and the least rapidly with range at Port Renfrew (the deepest model site). Propagation loss is greater in the summer compared to the winter at East Point, Mouat Point, Sheringham Point, and Sturdies Bay while smaller in the summer than in winter at Port Renfrew. There is little seasonal dependence of the propagation loss at Enterprise Reef, Swiftsure Bank, and Tilly Point. Fig 5 shows an example of propagation

loss modelled at Sturdies Bay in the 1900–2200 Hz band for winter and summer conditions. S13 Fig and S14 Fig show examples propagation loss modelled at the other locations.

Fig 5. Modelled propagation loss at Sturdies Bay in a) winter and b) summer for the 1900–2200 Hz band, as a function of range and depth along a single radial at a bearing of 40° (across the Strait of Georgia).

Table 3 summarizes the median detection range for all sites, call types, and seasons. Contours depicting the median detection range at each site for P = 0.5 for SRKW in summer and winter are shown in Fig 6. All detection range probabilities are plotted in Fig 7 and Fig 8 for SRKW and NRKW, respectively. Detection range estimates suggest that the East Point, Sturdies Bay, Tilly Point, and Enterprise Reef listening stations have the longest median detection ranges for SRKW pulsed calls (50% detection probability at 2.2, 4.2, 2.4, and 2.0 km, respectively, in winter; 7.9, 2.0, 2.0, and 2.2 km, respectively, in summer). The shortest SRKW median detection range (50% probability of detection) is modelled to be 650 m in winter, at Swiftsure Bank; in summer it is 1.1 km at Mouat Point. There is a 10% probability of SRKW pulsed calls being detected at median ranges between 1.5 and 25 km in winter or between 4.2 and 37.5 km in summer (site dependent). Pulsed calls can be detected with a 90% probability at site-dependent, median ranges between 130 and 540 m in winter and between 90 and 800 m in summer. The set of NRKW pulsed calls included in the study have smaller detection ranges than SRKW pulsed calls for all sites and seasons. The longest median detection ranges for NRKW pulsed calls are reached at Sheringham Point with 750 m in winter and 600 m in summer for P = 0.5. Swiftsure Bank has the shortest median detection range distance for NRKW pulsed calls; 240 m in winter and 510 m in summer (P = 0.5). NRKW pulsed calls, Sheringham Point has the longest median detection ranges with 750 m in winter and 600 m in summer for P = 0.5. For most sites, the detection ranges are shorter during winter than summer with the exception of Sturdies Bay for SRKW (Fig 7f) and Sheringham Point for NRKW (Fig 8b)

Table 3. Median detection range (in meters) for Probability of Detection (P) of 0.1, 0.5, and 0.9.

Model Site	Call type	Season	P = 0.1	P = 0.5	P = 0.9
**East Point**	**SRKW**	Winter	21,000	2,200	340
		Summer	21,700	7,900	800
**Enterprise Reef**	**SRKW**	Winter	25,000	2,000	150
		Summer	21,600	2,200	160
**Mouat Point**	**SRKW**	Winter	12,900	1,400	150
		Summer	37,500	1,100	90
**Port Renfrew**	**SRKW**	Winter	2,600	1,000	400

		Summer	4,500	1,400	470
	**NRKW**	Winter	900	300	160
		Summer	2,000	850	400
**Sheringham Point**	**SRKW**	Winter	1,500	850	440
		Summer	4,200	1,600	470
	**NRKW**	Winter	1,400	750	420
		Summer	1,900	600	210
**Sturdies Bay**	**SRKW**	Winter	10,800	4,200	130
		Summer	5,300	2,000	130
**Swiftsure Bank**	**SRKW**	Winter	2,000	650	270
		Summer	6,500	1,600	380
	**NRKW**	Winter	680	240	150
		Summer	1,600	510	190
**Tilly Point**	**SRKW**	Winter	8,600	2,400	540
		Summer	8,600	2,000	480

Fig 6. Median detection ranges for probability of detection, P, of 0.5 for SRKW in summer and winter at each model site.

Fig 7. Detection range probabilities for SRKW pulsed calls in summer (blue) and winter (orange). The solid lines are the median values, and the shaded areas define the 25th and 75th percentiles. Results are shown for (a) East Point, (b) Enterprise Reef, (c) Mouat Point, (d) Port Renfrew, (e) Sheringham Point, (f) Sturdies Bay, (g) Swiftsure Bank, and (h) Tilly Point.

Fig 8. Detection range probabilities for NRKW pulsed calls in summer (blue) and winter (orange). The solid lines are the median values, and the shaded areas define the 25th and 75th percentiles. Results are shown for (a) Port Renfrew, (b) Sheringham Point, and (c) Swiftsure Bank.

The maximum detection ranges for SRKW calls were most often associated with the lower frequency bands (below 8 kHz) but varied at each site and season due to the various ambient sound and propagation loss conditions (Fig 9, S11 Fig and S12 Fig). The maximum detection ranges for the set of NRKW calls were most often associated with higher frequency bands (above 10 kHz).

Fig 9. Percentage of the results for which each frequency band yielded the maximum detection range from the Monte Carlo simulation. Results are shown for SRKW at Sturdies Bay in (a) summer and (b) winter, for SRKW at Swiftsure

Bank in (c) summer and (d) winter, and at Swiftsure Bank in (e) summer and (f) winter. Results for all other locations are in S11 Fig and S12 Fig.

A sensitivity analysis was conducted to better understand which factors most influence the detection range results. This analysis focused on the detection range results for NRKW at Port Renfrew because NRKW have been observed at these locations but not at the other modelling sites. Detection ranges were recalculated several times by modifying in turn each of the following factors of the Monte Carlo process: source level frequency distribution (uniform versus negative slope, S15 Fig), ambient sound levels (winter versus summer, S16 Fig), the propagation loss (winter versus summer, S13 Fig, S14 Fig), and the animal depth distribution (uniform versus loglogistic, S17 Fig). Table 4 summarizes the results of the sensitivity analysis; the resulting detection range probabilities are compared in Fig 10. Fig 11 shows the frequency bands responsible for the maximum detection ranges for each of the two considered source level frequency distributions. This analysis showed that the frequency distribution of the source levels had the strongest effect on the detection range probability, the seasonal ambient sound levels had the second strongest influence (detection ranges were longer in summer), then the animal depth distribution (detection ranges were longer when whales were assumed to spend most of their time near the surface), and finally the seasonal propagation loss had a minimal effect on the detection range probabilities.

Table 4. Sensitivity analysis based on calculations for Northern Resident Killer Whale calls (NRKW-A) at Port Renfrew. Difference of the median detection range for P = 0.5 for each of the Monte Carlo factors tested.

Factor	Median detection range offset for P = 0.5 (m)
Frequency distribution of source levels<br>(no slope – negative slope)	850<br>(1700–850)
Ambient sound levels<br>(summer-winter)	600<br>(850–250)
Vocalization depth distribution<br>(log-logistic-uniform)	230<br>(850–620)
Propagation loss<br>(winter-summer)	30<br>(880–850)

Fig 10. Sensitivity analysis: Effect of changing the source level frequency distribution on the detection range, the seasonal ambient sound levels, the vocalization depth distribution, and the seasonal propagation loss. Detection range probabilities for Northern Resident Killer Whale (NRKW) pulsed calls at Port Renfrew comparing in turn (a) a negatively sloped frequency distribution of source levels (blue) and a uniform frequency distribution of source levels (orange) in summer, (b) summer ambient sound levels (blue) and winter ambient sound levels (orange), (c) summer propagation losses (blue) and winter propagation losses (orange), and (d) a log-logistic animal depth distribution (blue) and a uniform animal depth distribution (orange) for summer. In each panel the solid lines are the median values and the shaded areas define the 25th and 75th percentiles.

Fig 11. Sensitivity analysis: Effect of the source level frequency distribution on the detection bands on the frequency of detection. Percentage of the results for which each frequency band yielded the maximum detection range from the Monte Carlo simulation for Northern Resident Killer Whale (NRKW) in summer, using (a) a negatively sloped frequency distribution of source levels and (b) a uniform frequency distribution of source levels.

Discussion

In this study we evaluated the detection range of SRKW and NRKW at different seasons and different locations in the Salish Sea. Detection ranges fluctuated substantially between locations. PAM stations around the Southern Gulf Islands (Sturdies Bay, Enterprise Reef, Mouat Point, Tilly Point and East Point) had larger detection ranges than stations located in the Strait of Juan de Fuca and on Swiftsure Bank (Sheringham Point, Port Renfrew, Swiftsure Bank). This difference is driven by the higher ambient sound levels measured in the Juan de Fuca Strait than in the protected inshore waters of the Salish Sea that are favoring acoustic masking (S9 Fig and S10 Fig). This difference in ambient sound levels observed from our data is consistent with other acoustic measurements conducted in the Salish Sea (65). The Juan de Fuca Strait is a major shipping lane funneling commercial vessels transiting from the Pacific to several ports in both the USA and Canada, including the Port of Vancouver, which is the largest port in Canada and the third largest in North America by tons of cargo (66). Because it is facing the Pacific Ocean, the soundscape in the Juan de Fuca Strait is influenced by both local and offshore sources. This makes this area sometimes naturally noisier than the inshore waters of the Salish Sea, which increases acoustic masking of killer whale calls and reduces the detection range. Mouat Point is one of the locations with the shortest detection range. This PAM station is located at the intersection of several ferry routes connecting Vancouver Island, the Southern Gulf Islands, and Vancouver on Canada’s mainland. As a result, it is noisier than the other locations in the area (S9 Fig), leading to lower detections ranges. In winter, Sturdies Bay has lower ambient sound levels than the other locations (S9 Fig) which results in greater detection ranges (Fig 7). Sturdies Bay is in Active Pass, which is a major thoroughfare for both commercial vessels including ferries and fishing vessels, as well as recreational vessels. The latter are more frequently present during the summer when there are also many more ferries transiting through the Pass compared to winter (67). While this location may indeed be quieter, ambient sound data from this location and season were more limited than others (n=5126), and ambient sound condition may be underestimated. In summer, East Point has the largest detection range for SRKW. This is mostly due to the lower ambient sound levels in the frequency band 2-3 kHz (Fig S9) which is the band responsible for more than 50% of the maximum detection range realizations (Fig S11). The East Point PAM is in Boundary Pass, which is a waterway used by

commercial vessels on their way to and from the Port of Vancouver but is not used by ferries. While ferries travel on regular schedules throughout the day, freight carrying vessels pass the East Point PAM on a more intermittent schedule sometimes with long periods between vessels. This may have influenced the ambient sound level measurements. Furthermore, in contrast to the Tilly Point PAM, which is also located in Boundary Pass but its soundscape is also influenced by activities in two adjacent straits. The East Point PAM is a little bit sheltered from sounds coming from other areas than Boundary Pass.

SRKW median detection ranges ($P = 0.5$) are longer in summer than winter at five of the eight modelled locations (Table 3) and similar at two locations (Mouat Point and Tilly Point). The difference between summer and winter is mainly due to the seasonal variability in ambient sound levels at these locations. Ambient sound in the Salish Sea, as in most places, is known to be consistently more elevated in the winter than in the summer due to stronger winds (65, 68). Sturdies Bay is an exception and has shorter detection ranges in summer than winter. As already indicated, this is most likely due to the under sampling of ambient sound conditions in winter due to the limited data available and due to the aforementioned differences of the ambient conditions in summer and winter. Several types of acoustic recorders were used to collect the ambient data used for the summer and winter modelling. Differences in the mooring configurations, recorder depths, and recorder and hydrophone sensitivities could result in differences between the summer and winter ambient conditions that are not strictly due to natural seasonal differences.

Detection ranges for the subset of calls of NRKW that were investigated are shorter than those of SRKW (Table 3). This difference is explained by the different source levels of these NRKW calls versus SRKW calls. Stereotyped pulsed calls were from one acoustic clan of NRKW collected in a quieter environment than the locations of the modelled detection range. The NRKW calls have a reported mean source level approximately 7 dB lower than SRKW (Table 1) and this is consequently the reason why NRKW calls were masked at much shorter distances. The frequency components of the killer whale calls responsible for the maximum detection range differ between NRKW and SRKW. Maximum detection ranges tend to be mostly achieved from the higher frequency component of the calls of NRKW (i.e. > 6 kHz, Fig S12) while maximum detection ranges for SRKW calls were largely reached at lower frequencies (i.e. < 4 kHz, Fig S11). However, some of the NRKW calls showed higher source levels at frequencies above 5 kHz than at lower frequencies (Fig 2d) and localized level maxima were also present in higher frequencies in SRKW calls (Fig 2 b). This is likely the result of calls with more than one distinct frequency component, a low frequency (LF) and a high frequency (HF) component (Fig 12, (69)).

Fig 12. Examples of two-component discrete pulsed calls of a) SRKW and b) NRKW. The lower frequency components (LFCs) are transmitted omnidirectionally while the higher frequency components (HFCs) that may be produced parallel to the LFCs transmit more directionally (Miller 2002)

The HF component transmits more directionally (51) and therefore shows a higher source level than the corresponding frequency of the LF component when transmitted over shorter distances. While directionality likely accounted for in the variability of the broadband source levels, we did not have enough information about the directivity patterns of the collected calls by frequency to be able to incorporate it in the detection range estimates. Call source levels may not be static and may vary based on the ambient sound level in which they are produced. Holt et al. (55) suggested that SRKW increase broadband call source levels in noisier environments (i.e. Lombard effect). Incorporating this effect to the detection range modeling is possible but would require a more comprehensive characterization (e.g., more individual call samples from more locations) to benefit the detection range estimates. Based on empirical measurements, the energy of NRKW calls is more homogeneously distributed across frequencies than SRKW calls (i.e., decrease of -0.27 dB per 300 Hz band for NRKW vs -0.66 dB per 300 Hz band for SRKW, Fig 2). For SRKW, because most of the energy is concentrated at low frequencies, low frequency components of the calls can be detected at farther ranges than the high frequency components that are attenuated rapidly. The NRKW call detection ranges were modeled in an environment where ocean ambient sound is more pronounced at low frequencies (Juan de Fuca Strait off Port Renfrew), and since the energy of NRKW calls is spread relatively evenly across frequencies, it means that the low-frequency components of their calls are quickly masked. As a result, the higher-frequency components of killer whale calls, which fall outside the primary masking range, are detected at greater distances. Yurk et al. (39) suggested that calls may have adapted design features, such as two-voice calling to reduce masking. For example, two-voice calling is a strategy used by Emperor penguins to find their mate after returning to a noisy colony (70) . The sensitivity analysis conducted showed that the frequency distribution of source levels and the sound levels were the two factors that have the greatest influence on the detection range modeling. Consequently, it is important to carefully characterize these two factors to obtain the most realistic detection range estimates. It is important to set the maximal detection frequency of the automated detector high enough to capture both the low and high frequency components of calls.

It is relatively straightforward to estimate ambient sound levels using *in situ* measurements. Here we used sound pressure levels from recordings collected by calibrated instruments. We used sound pressure levels averaged over 1 minute as it is becoming a common time resolution for soundscape analysis (71-73) The sensitivity analysis showed that using shorter noise windows does not affect the outcome of the detection range modeling substantially (S3 Table) Using as much data as

possible lead to represent the variations of ambient sound conditions at each study site and provides more reliable detection range estimates. We used at least 10,000 ambient sound measurements for each modelling scenario. Because of the limited data available, we could only use 5,000 ambient sound measurements for Studies Bay in winter. Consequently, results from this modeling scenario should be interpreted with caution. The frequency distribution of source levels is the most sensitive parameter for the detection range modeling. It is also the most difficult to estimate. Source levels of killer whale calls (or whale calls in general) are typically only reported as normally distributed values (characterized by their mean and standard deviation) covering all frequencies of the call (i.e., broadband). Here, to estimate the relative frequency distribution of source levels, we used measurements of killer whale calls with high SNR, assuming that the animals were close to the instrument and that the acoustic attenuation was negligeable. Based on local knowledge of the area, we believe that this is a realistic assumption. However, performing acoustic localization and backpropagating received levels would be preferrable (when possible). Also, it is important to gather a comprehensive sample of calls preferably collected at various locations with different ambient sound levels. This is often difficult to achieve as one also requires calls with high SNR. The potential effect of the dual components of killer whale calls on the detection range could not be fully assessed in this study because of lack of sufficient examples from different environments. It would need to be considered, however, when detection range is modelled in quieter locations. The dual component influence on the source levels may explain the lower levels considered for NRKW calls in this study compared to those reported elsewhere (50). One of the important results of this study, however, is the demonstration of an influence of source levels in different frequency bands on the estimated call detection rate. In order to improve the reliability of detection range estimates, we encourage researchers to report source level measurements not only as a broadband value but always in different frequency bands. Vocalization depth distribution and the difference in propagation loss did not substantially influence the outcome of the detection range modeling. While there are differences in propagation loss values between summer and winter, the differences occur mainly at long ranges (beyond several kilometers). Consequently, the median detection ranges are not affected appreciably by these seasonal differences of the propagation loss.

The approach used in this paper is designed to compute the probability of detecting a single vocalizing whale. If many whales are co-located and vocalizing together, then the probability of detection is expected to increase due to the increased volume of calls produced at different distances from the receiving hydrophone. One of the important reasons for this study was to determine if automated acoustic detection can ~~possibly~~ be used effectively in disturbance mitigation. In this scenario it is important to detect the first few calls at the greatest distance possible at each location. As such the focus on a call produced

by a single individual provides information about the best possible scenario. Detecting a single vocalizing whale becomes even more relevant if the source of disturbance is moving as well, such as a vessel because the effect of vessel strike experienced at the individual level. These results, therefore, can be considered a conservative characterization of the detection range probabilities. All detection range probabilities presented were estimated using a detection threshold of 5 dB (Eq 2). This parameter is dependent on the detection process used and will vary depending on the monitoring scenario. If all acoustic data are manually annotated by an analyst, the detection threshold would likely be closer to 0 dB. The performance of an automated detector, however, needs to be quantified by signal-to-noise ratio intervals in order to select the appropriate detection threshold value (67). One needs to define a satisfactory precision and recall level depending on the purpose of the system, e.g. whale tracking via a network of PAM systems located at various geographic locations to alert vessel operators of the presence of whales. Results from our study suggest that a network of PAM stations will be most effective at detecting the presence of killer whales (including SRKW) during the summer, and better at some locations than others. While the proposed approach is focusing on the detectability of killer whales, it can be easily generalized to other species by adapting the dive profiles (if tag data are available) and using an appropriate propagation loss model. The proposed approach could also assist when designing a passive acoustic monitoring plan for mitigation of noise exposure during commercial activities that produce high sound levels by determining PAM locations that maximize detection ranges at times when the activities occur. In critical areas, using multiple synchronized hydrophones would allow to perform beamforming and add an array gain to the Eq 1 which would substantially increase the detection range (46, 74). Note that future work will include field studies to ground truth the detection range estimates from the modeling with empirical *in situ* measurements. This analysis also highlighted the value of knowing the frequency distribution of source levels. We would encourage future studies to report source levels in different frequency bands rather than only broadband values.

Conclusion

Estimating the detection range of marine mammal sounds is important for designing and interpreting results from passive acoustic monitoring studies and when designing monitoring networks to track whales acoustically. Yet, it is not often characterized. In this study we estimated the detection range of stereotypic pulsed calls from Southern and Northern Resident Killer Whales at several passive monitoring stations deployed by Fisheries and Ocean Canada in waters off the British

Columbia coast. The detection range was estimated using ambient sound levels measured in situ, modelled propagation losses determined by the acoustic model MONM and applied Monte Carlo simulations to capture the variability in call source level and animal depth. Beside indicating the probability of detection at each location, the approach employed informs on the frequency bands of the killer whale calls that are the most important for being detected at range. We show that the distribution of the source level of calls across frequencies in conjunction with the ambient sound level are principal factors that affect detection ranges, but are rarely reported

Acknowledgments

We would like to thank Brianna Wright (Fisheries and Oceans Canada, DFO) for providing animal depth data from DTAGs, Svein Vagle (DFO) and Caitlin O'Neil (DFO) for providing the acoustic data from Swiftsure Bank and Port Renfrew, Paul Cottrell (DFO) for providing the acoustic data from the Whale Tracking Network operated by DFO Marine Mammal Management, and the Vancouver Fraser Port Authority and Ocean Networks Canada for supporting the data acquisition at East Point. Northern Resident Killer Whale calls used in this study were collected by Jennifer Wladichuk (JASCO Applied Sciences) with field support from Jens Koblitz (Max Planck Institute of Animal Behavior), Peter Van Buren (DFO), and Marie Zahn (University of Washington). This study was funded by Fisheries and Oceans Canada and JASCO Applied Science. XM also received support from the Investment in Science Fund at the Woods Hole Oceanographic Institution.

References

1. Allen AM, Singh NJ. Linking Movement Ecology with Wildlife Management and Conservation. Frontiers in Ecology and Evolution. 2016;3.
2. Hawkins ER, Harcourt R, Bejder L, Brooks LO, Grech A, Christiansen F, et al. Best Practice Framework and Principles for Monitoring the Effect of Coastal Development on Marine Mammals. Frontiers in Marine Science. 2017;4.
3. Runge CA, Martin TG, Possingham HP, Willis SG, Fuller RA. Conserving mobile species. Frontiers in Ecology and the Environment. 2014;12(7):395-402.
4. Verfuss UK, Gillespie D, Gordon J, Marques TA, Miller B, Plunkett R, et al. Comparing methods suitable for monitoring marine mammals in low visibility conditions during seismic surveys. Marine Pollution Bulletin. 2018;126:1-18.
5. Zimmer WMX. Passive acoustic monitoring of cetaceans. Cambridge, UK: Cambridge University Press; 2011. 366 p.
6. de Vere AJ, Lilley MK, Frick EE. Anthropogenic impacts on the welfare of wild marine mammals. Aquatic Mammals. 2018;44(2).
7. Ford JKB, Ellis GM. Selective foraging by fish-eating killer whales *Orcinus orca* in British Columbia. Marine Ecology Progress Series. 2006;316:185-99.

8. Research CfW. Southern Resident Orca Population 2024 [Available from: https://www.whaleresearch.com/orca-population.
9. [DFO] Fisheries and Oceans Canada. Population status update for the Northern Resident Killer Whale (Orcinus orca) in 2022. . 2023.
10. Sobocinski KL, Harvell CD, Baloy NJK, Broadhurst G, Dethier MN, Flower A, et al. Urban seas as hotspots of stress in the Anthropocene ocean: The Salish Sea example. Elementa: Science of the Anthropocene. 2022;10(1).
11. [DFO] Fisheries and Oceans Canada. Recovery Strategy for the Northern and Southern Resident Killer Whales (Orcinus orca) in Canada [Proposed]. 2018.
12. Ettinger AK, Harvey CJ, Emmons C, Hanson MB, Ward EJ, Olson JK, et al. Shifting phenology of an endangered apex predator mirrors changes in its favored prey. . Endanger Spec Res. 2022;48:Pages 211-23.
13. Olson JK, Wood J, Osborne RW, Barrett-Lennard LG, Larson S. Sightings of southern resident killer whales in the Salish Sea 1976-2014: the importance of a long-term opportunistic dataset. Endanger Spec Res. 2018;37:Pages 105-18.
14. Pilkington J, Stredulinsky EH, Gavrilchuk K, Thornton S, Ford J, Doniol-Valcroze T. Patterns of winter occurrence of three sympatric killer whale populations off eastern Vancouver Island, Canada, based on passive acoustic monitoring. Frontiers in Marine Science. 2023;10.
15. Shields MW, Hysong-Shimazu S, Shields JC, Woodruff J. Increased presence of mammal-eating killer whales in the Salish Sea with implications for predator-prey dynamics. . PeerJ 6:e6062 https://doiorg/107717/peerj6062. 2018.
16. Shields MW, Lindell J, Woodruff J. Declining spring usage of core habitat by endangered fish-eating killer whales reflects decreased availability of their primary prey. Pacific Conservation Biology. 2018;24:pages 189-93.
17. [DFO] Fisheries and Oceans Canada. Action Plan for the Northern and Southern Resident Killer Whale (Orcinus orca) in Canada. Species at Risk Act Action Plan Series.; 2017.
18. Hannay DE, Delarue J, Mouy X, Martin BS, Leary D, Oswald JN, et al. Marine mammal acoustic detections in the northeastern Chukchi Sea, September 2007–July 2011. Continental Shelf Research. 2013;67:127-46.
19. Van Parijs SM, Clark CW, Sousa-Lima RS, Parks SE, Rankin S, Risch D, et al. Management and research applications of real-time and archival passive acoustic sensors over varying temporal and spatial scales. Marine Ecology Progress Series. 2009;395:21-36.
20. Sousa-Lima RS, Norris TF, Oswald JN, Fernandes DP. A review and inventory of fixed autonomous recorders for passive acoustic monitoring of marine mammals. Aquatic Mammals. 2013;39(1):23-53.
21. Browning E, Gibb R, Glover-Kapfer P, Jones KE. Passive acoustic monitoring in ecology and conservation. Woking, UK: WWF-UK; 2017.
22. Gibb R, Browning E, Glover-Kapfer P, Jones KE. Emerging opportunities and challenges for passive acoustics in ecological assessment and monitoring. Methods in Ecology and Evolution. 2019;10(2):169-85.
23. Ford J. Dialects. in Encyclopedia of Marine Mammals: Academic Press; 2018. p. 253-4.
24. Bradley Hanson M, Emmons CK, Ward EJ, Nystuen JA, Lammers MO. Assessing the coastal occurrence of endangered killer whales using autonomous passive acoustic recorders. The Journal of the Acoustical Society of America. 2013;134(5):3486-95.
25. Burham RE, Palm RS, Duffus DA, Mouy X, Riera A. The combined use of visual and acoustic data collection techniques for winter killer whale (Orcinus orca) observations. Global Ecology and Conservation. 2016;8:24-30.
26. Emmons CK, Hanson MB, Lammers MO. Passive acoustic monitoring reveals spatiotemporal segregation of two fish-eating killer whale Orcinus orca populations in proposed critical habitat. Endanger Spec Res. 2021;44:253-61.
27. Leu AA, Hildebrand JA, Rice A, Baumann-Pickering S, Frasier KE. Echolocation click discrimination for three killer whale ecotypes in the Northeastern Pacific. The Journal of the Acoustical Society of America. 2022;151(5):3197-206.

28. Myers HJ, Olsen DW, Matkin CO, Horstmann LA, Konar B. Passive acoustic monitoring of killer whales (Orcinus orca) reveals year-round distribution and residency patterns in the Gulf of Alaska. Scientific Reports. 2021;11(1):20284.
29. Rice A, Deecke VB, Ford JKB, Pilkington JF, Oleson EM, Hildebrand JA, et al. Spatial and temporal occurrence of killer whale ecotypes off the outer coast of Washington State, USA. Marine Ecology Progress Series. 2017;572:255-68.
30. Riera A, Ford JK, Hildebrand JA, Chapman NR. Acoustic monitoring of killer whale populations off the west coast of Vancouver Island. The Journal of the Acoustical Society of America. 2011;129(4_Supplement):2607-.
31. Matkin CO, Ellis G, Barrett-Lennard LG, Yurk H, Saulitis E, Scheel D, et al. Photographic and acoustic monitoring of killer whales in Prince William Sound and Kenai Fjords. 2003.
32. Yurk H, Filatova OA, Matkin CO, Barrett-Lennard LG, Brittain M. Sequential Habitat Use by Two Resident Killer Whale (Orcinus orca) Clans in Resurrection Bay, Alaska as Determined by Remote Acoustic Monitoring. Aquatic Mammals. 2010;36(1).
33. Schall E, Van Opzeeland IC. Calls produced by Ecotype C killer whales (Ocinus orca) off the Eckstroem Iceshelf, Antarctica. Aquatic Mammals. 2017;43(2):117-26.
34. Riera A, Pilkington JF, Ford JKB, Stredulinsky EH, Chapman NR. Passive acoustic monitoring off Vancouver Island reveals extensive use by at-risk Resident killer whale (Orcinus orca) populations. Endanger Spec Res. 2019;39:221-34.
35. Rand ZR, Wood JD, Oswald JN. Effects of duty cycles on passive acoustic monitoring of southern resident killer whale (Orcinus orca) occurrence and behavior. The Journal of the Acoustical Society of America. 2022;151(3):1651-60.
36. Frasier KE, Garrison LP, Soldevilla MS, Wiggins SM, Hildebrand JA. Cetacean distribution models based on visual and passive acoustic data. Scientific Reports. 2021;11(1):8240.
37. Simard Y, Roy N. Detection and localization of blue and fin whales from large-aperture autonomous hydrophone arrays: A case study from the St. Lawrence estuary. Can Acoustics. 2008;36(1):104-10.
38. Vagle S, Burnham RE, O'Neill C, Yurk H. Variability in Anthropogenic Underwater Noise Due to Bathymetry and Sound Speed Characteristics. Journal of Marine Science and Engineering. 2021;9(10):1047.
39. Yurk H, O'Neill C, Quayle LS, Vagle S, Mouy X, Austin M, et al. Adaptive Call Design to Escape Masking While Preserving Complex Social Functions of Calls in Killer Whales. In: Popper AN, Sisneros J, Hawkins AD, Thomsen F, editors. The Effects of Noise on Aquatic Life: Principles and Practical Considerations. Cham: Springer International Publishing; 2023. p. 1-15.
40. Clark CW, Ellison WT, Southall BL, Hatch L, Van Parijs SM, Frankel A, et al. Acoustic masking in marine ecosystems: intuitions, analysis, and implication. Marine Ecology Progress Series. 2009;395:201-22.
41. Stafford KM, Mellinger DK, Moore SE, Fox CG. Seasonal variability and detection range modeling of baleen whale calls in the Gulf of Alaska, 1999–2002. The Journal of the Acoustical Society of America. 2007;122(6):3378-90.
42. Samaran F, Adam O, Guinet C. Detection range modeling of blue whale calls in Southwestern Indian Ocean. Applied Acoustics. 2010;71(11):1099-106.
43. Helble TA, D'Spain GL, Hildebrand JA, Campbell GS, Campbell RL, Heaney KD. Site specific probability of passive acoustic detection of humpback whale calls from single fixed hydrophones. The Journal of the Acoustical Society of America. 2013;134(3):2556-70.
44. Shabangu FW, Yemane D, Stafford KM, Ensor P, Findlay KP. Modelling the effects of environmental conditions on the acoustic occurrence and behaviour of Antarctic blue whales. PLoS ONE. 2017;12(2):e0172705.
45. Shabangu FW, Yemane D, Best G, Estabrook BJ. Acoustic detectability of whales amidst underwater noise off the west coast of South Africa. Marine Pollution Bulletin. 2022;184:114122.
46. Urick RJ. Principles of Underwater Sound. 3rd ed. New York, London: McGraw-Hill; 1983. 423 p.
47. Ford JKB. Vocal traditions among resident killer whales (*Orcinus orca*) in coastal waters of British Columbia. Can J Zool. 1991;69(6):1454-83.

48. Heise K, Barrett-Lennard L, Chapman R, Dakin T, Erbe C, Hannay DE, et al. Proposed Metrics for the Management of Underwater Noise for Southern Resident Killer Whales. Vancouver, Canada: Report for the Coastal Ocean Research Institute. © Ocean Wise 2017; 2017.
49. Ford JKB. Acoustic behaviour of resident killer whales (*Orcinus orca*) off Vancouver Island, British Columbia. Can J Zool. 1989;67(3):727-45.
50. Miller PJ. Diversity in sound pressure levels and estimated active space of resident killer whale vocalizations. Journal of Comparative Physiology A. 2006;192:449-59.
51. Miller PJO. Mixed-directionality of killer whale stereotyped calls: A direction of movement cue? Behavioral Ecology and Sociobiology. 2002;52(3):262-70.
52. Holt MM. Sound exposure and southern resident killer whales (Orcinus orca): A review of current knowledge and data gaps. 2008.
53. Holt MM, Noren DP, Emmons CK. Effects of noise levels and call types on the source levels of killer whale calls. Journal of the Acoustical Society of America. 2011;130(5):3100-6.
54. Wladichuk J, Dosso S, Lawson J, Koblitz J. Source level measurements of killer whale echolocation clicks. . 179th Acoustical Society of Americal Virtual Conference2020.
55. Holt MM, Noren DP, Veirs V, Emmons CK, Veirs S. Speaking up: Killer whales (*Orcinus orca*) increase their call amplitude in response to vessel noise. Journal of the Acoustical Society of America. 2009;125(1):EL27-EL32.
56. Ford J. A catalogue of underwater calls produced by killer whales (Orcinus orca) in British Columbia. . 1987.
57. Collins MD. A split-step Padé solution for the parabolic equation method. Journal of the Acoustical Society of America. 1993;93(4):1736-42.
58. Porter MB, Bucker HP. Gaussian beam tracing for computing ocean acoustic fields. Journal of the Acoustical Society of America. 1987;82(4):1349-59.
59. High resolution NOAA digital elevation model [Internet]. National Oceanic and Atmospheric Administration, U.S. Department of Commerce. 2013. Available from: http://www.ngdc.noaa.gov/dem/squareCellGrid/download/655.
60. Howes DE, Zacharias MA, Harper JR. British Columbia Marine Ecological Classification : Marine Ecosections and Ecounits: Coastal Task Force, Resources Inventory Committee; 1997.
61. Buckingham MJ. Compressional and shear wave properties of marine sediments: Comparisons between theory and data. Journal of the Acoustical Society of America. 2005;117(1):137-52.
62. Carnes MR. Description and Evaluation of GDEM-V 3.0. US Naval Research Laboratory, Stennis Space Center, MS; 2009. Contract No.: NRL Memorandum Report 7330-09-9165.
63. Teague WJ, Carron MJ, Hogan PJ. A comparison between the Generalized Digital Environmental Model and Levitus climatologies. JGR. 1990;95(C5):7167-83.
64. Coppens AB. Simple equations for the speed of sound in Neptunian waters. Journal of the Acoustical Society of America. 1981;69(3):862-3.
65. Burnham RE, Vagle S, O'Neill C. Spatiotemporal patterns in the natural and anthropogenic additions to the soundscape in parts of the Salish Sea, British Columbia, 2018–2020. Marine Pollution Bulletin. 2021;170:112647.
66. [VFPA] Vancouver Fraser Port Authority. Port Information Guide. 2024.
67. Yurk H, Quayle L, Burnham R, MacConnachie S, LeBlond T. Evaluating effectiveness of shore cabled hydrophone networks as near real-time killer whale detection and tracking systems with special reference to DFO's Whale Tracking Network (WTN). Can Tech Rep Fish Aquat Sci. 2023;3543:ix:69 p.
68. Yang Z, García-Medina G, Wu W-C, Wang T, Leung LR, Castrucci L, et al. Modeling analysis of the swell and wind-sea climate in the Salish Sea. Estuar Coast Shelf Sci. 2019;224:289-300.
69. Miller PJO, Bain DE. Within-pod variation in the sound production of a pod of killer whales, Orcinus orca. Animal Behaviour. 2000;60(5):617-28.
70. Aubin T, Jouventin P, Hildebrand C. Penguins use the two-voice system to recognize each other. Proceedings of the Royal Society B: Biological Sciences. 2000;267(1448):1081-7.

71. Hatch L, Barkowski J, Baumann-Pickering S, Bell CW, Best B, Brown J, et al. SanctSound: Building Data Systems for Sound Decisions. In: Popper AN, Sisneros JA, Hawkins AD, Thomsen F, editors. The Effects of Noise on Aquatic Life: Principles and Practical Considerations. Cham: Springer International Publishing; 2024. p. 1547-57.
72. Wall C, Hatch L, Van Parijs S, Bochenek R, Berchok C, Davis G, et al. Connecting separate monitoring programs through the SoundCoop. The Journal of the Acoustical Society of America. 2024;155(3_Supplement):A97-A.
73. Ainslie MA, Miksis-Olds JL, Martin B, Heaney K, de Jong CAF, von Benda-Beckmann AM, et al. ADEON Underwater Soundscape and Modeling Metadata Standard. Version 1.0. Technical report by JASCO Applied Sciences for ADEON Prime Contract No. M16PC00003; 2018.
74. Kay S. Fundamentals of Statistical Signal Processing. II. Dection Theory. Upper Saddle River, NJ: Prentice Hall; 1998.

Supporting information

S1 Table. End points of model transects originating from each model site.

S2 Table. Geoacoustic profiles applied at the model sites.
Within each depth range, each parameter varies linearly within the stated range.

S3 Table. Sources of acoustic data used for ambient sound level measurements at each of the model sites, and for each modelled season.

S4 Table. Median detection ranges (in meters) for Probability of Detection (P) of 0.1, 0.5 and 0.9 for Monte Carlo simulations run using either 10 second or 60 second data windows to compute the ambient noise levels (computed for 1 week of ambient data).

S1 Fig. Geological Survey of Canada (GSC) and the BC Marine Ecological Classification (BCMEC) geological map of the western British Columbian coast

S2 Fig. Sound speed profiles Sound speed profiles used to model winter (blue) and summer (red) conditions at each of the model sites.

S3 Fig. Distribution of broadband source levels sampled in the Monte Carlo process for Southern Resident Killer Whale (SRKW) call detection range calculations. For (left) summer and (right) winter background conditions at (top to bottom) East Point, Enterprise Reef, Mouat Point, Port Renfrew, Sheringham Point, Sturdies Bay, Swiftsure Bank, and Tilly Point.

S4 Fig. Distribution of broadband source levels sampled in the Monte Carlo process for Northern Resident Killer Whale (NRKW) call detection range calculations. For (left) summer and (right) winter background conditions at (top to bottom) Port Renfrew, Sheringham Point, and Swiftsure Bank.

S5 Fig. Statistical distribution of source levels in 300 Hz bands from the Monte Carlo process for Southern Resident Killer Whale (SRKW) call detection range calculations, as a function of frequency. For (left) summer and (right) winter background conditions at (top to bottom) East Point, Enterprise Reef, Mouat Point, Port Renfrew, Sheringham Point, Sturdies Bay, Swiftsure Bank, and Tilly Point.

S6 Fig. Statistical distribution of source levels in 300 Hz bands from the Monte Carlo process for Northern Resident Killer Whale (NRKW) call detection range calculations, as a function of frequency. For (left) summer and (right) winter background conditions at (top to bottom) Port Renfrew, Sheringham Point, and Swiftsure Bank.

S7 Fig. Distribution of vocalization depths sampled in the Monte Carlo process for Southern Resident Killer Whale (SRKW) call detection range calculations, as a function of frequency. For (left) summer and (right) winter background conditions at (top to bottom) East Point, Enterprise Reef, Mouat Point, Port Renfrew, Sheringham Point, Sturdies Bay, Swiftsure Bank, and Tilly Point.

S8 Fig. Distribution of vocalization depths sampled in the Monte Carlo process for Northern Resident Killer Whale (NRKW) call detection range calculations, as a function of frequency. For (left) summer and (right) winter background conditions at (top to bottom) Port Renfrew, Sheringham Point, and Swiftsure Bank.

S9 Fig. Percentile distribution of ambient sound levels (in 300 Hz bands) from the recorded data samples used in the Monte Carlo process for Southern Resident Killer Whale (SRKW) call detection range calculations, as a function of frequency. For (left) summer and (right) winter background conditions at (top to bottom) East Point, Enterprise Reef, Mouat Point, Port Renfrew, Sheringham Point, Sturdies Bay, Swiftsure Bank, and Tilly Point.

S10 Fig. Percentile distribution of ambient sound levels (in 300 Hz bands) from the recorded data samples used in the Monte Carlo process for Northern Resident Killer Whale (NRKW) call detection range calculations, as a function of frequency. For (left) summer and (right) winter background conditions at (top to bottom) Port Renfrew, Sheringham Point, and Swiftsure Bank.

S11 Fig. Percentage of the results for which each frequency band yielded the maximum detection range in the Monte Carlo process for Southern Resident Killer Whale (SRKW) call detection range calculations, as a function of frequency. For (left) summer and (right) winter background conditions at (top to bottom) East Point, Enterprise Reef, Mouat Point, Port Renfrew, Sheringham Point, Sturdies Bay, Swiftsure Bank, and Tilly Point.

S12 Fig. Percentage of the results for which each frequency band yielded the maximum detection range in the Monte Carlo process for Northern Resident Killer Whale (NRKW) call detection range calculations, as a function of frequency. For (left) summer and (right) winter background conditions at (top to bottom) Port Renfrew, Sheringham Point, and Swiftsure Bank.

S13 Fig. Propagation loss modelled in the 1900–2200 Hz band at a-b) East Point, c-d) Enterprise Reef, e-f) Mouat Point, and g-h) Port Renfrew, for winter (left column) and summer (right column) conditions, as a function of range and depth along a single radial.

S14 Fig. Propagation loss modelled in the 1900–2200 Hz band at a-b) Sheringham Point, c-d) Sturdies Bay, e-f) Swiftsure Bank, and g-h) Tilly Point, for winter (left column) and summer (right column) conditions, as a function of range and depth along a single radial.

S15 Fig. a) Negatively slopped and b) uniform frequency distribution of Northern Resident Killer Whale (NRKW) source levels used for the sensitivity analysis. Statistical distribution of source levels (in 300 Hz bands) from the Monte Carlo simulation, as a function of frequency.

S16 Fig. a) Winter and b) summer ambient levels at Port Renfrew used for the sensitivity analysis. Percentile distribution of background sound levels (in 300 Hz bands).

S17 Fig. a) Uniform and b) log-logistic depth distributions used for the sensitivity analysis.

Fig 1

Click here to access/download;Figure;Fig1.tif

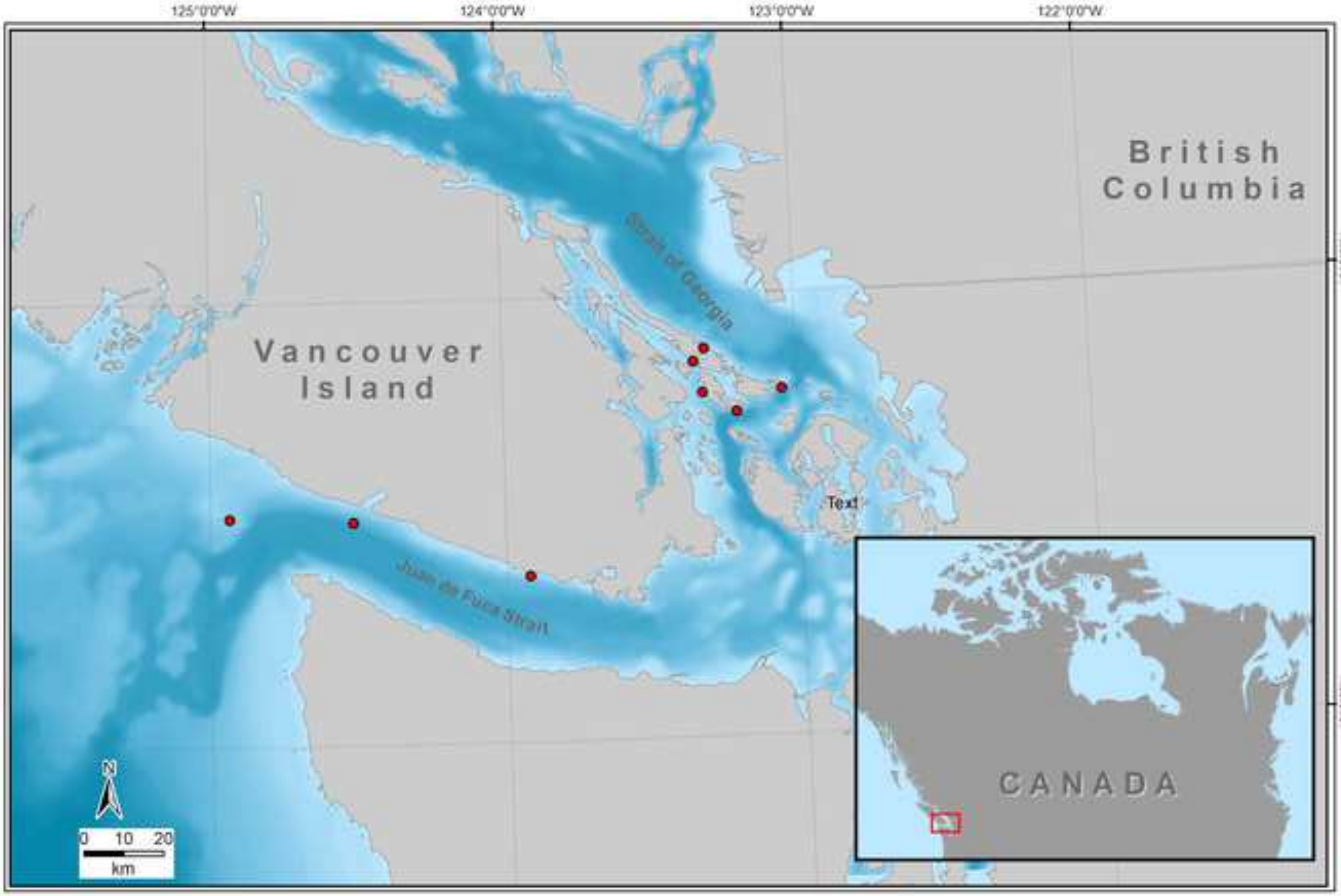

Fig 2

Click here to access/download;Figure;Fig2.tif

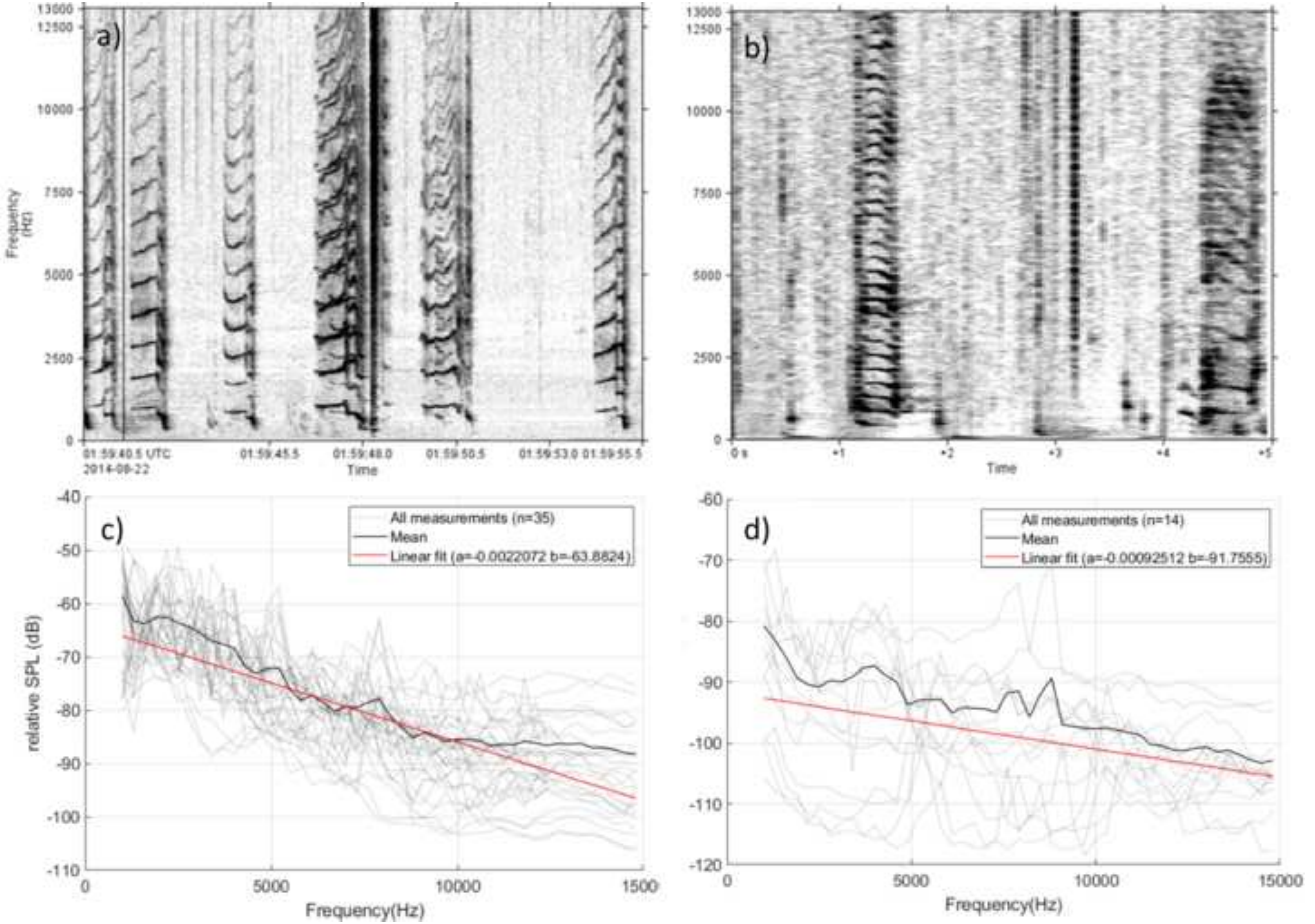

Click here to access/download;Figure;Fig3.tif

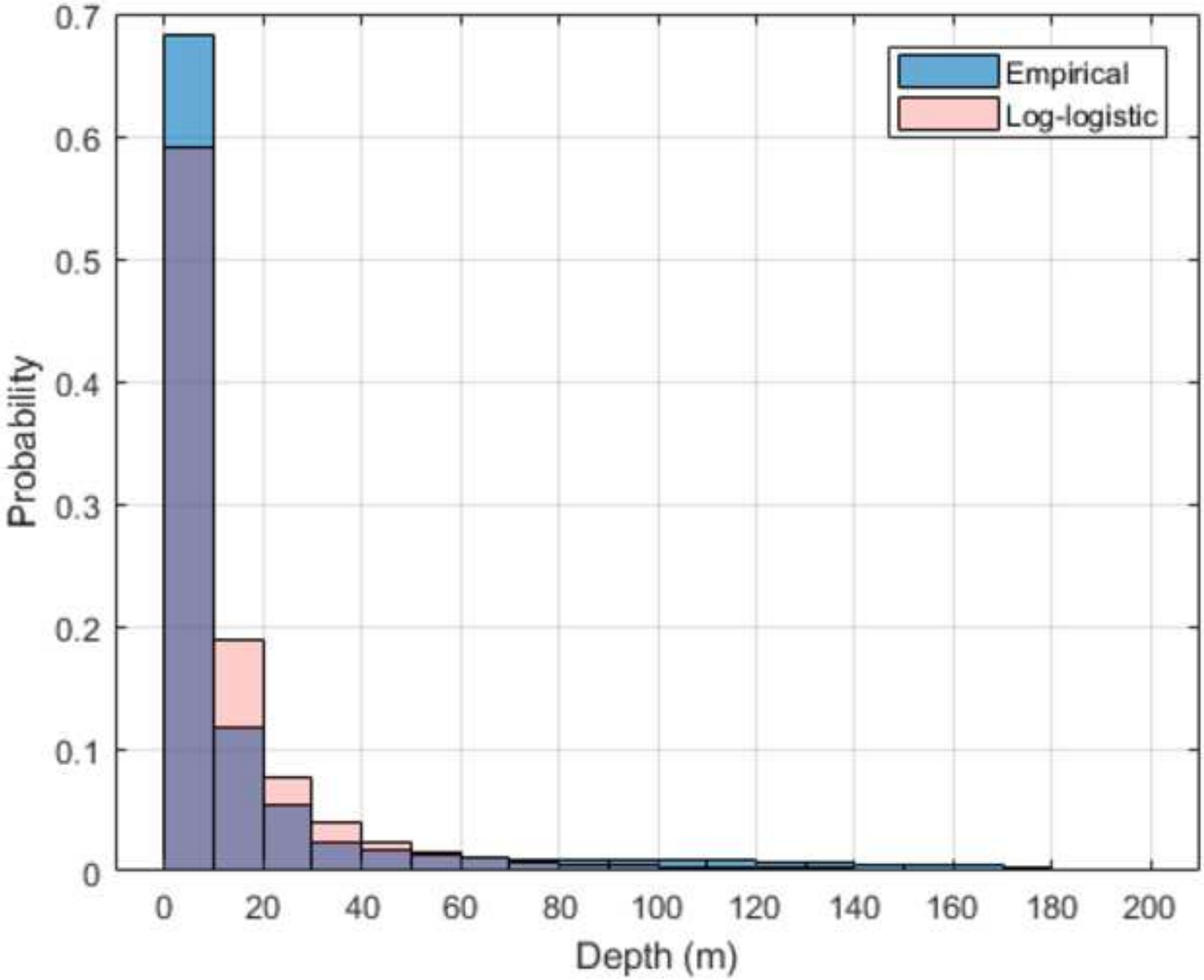

Fig 4

Click here to access/download;Figure;Fig4.tif

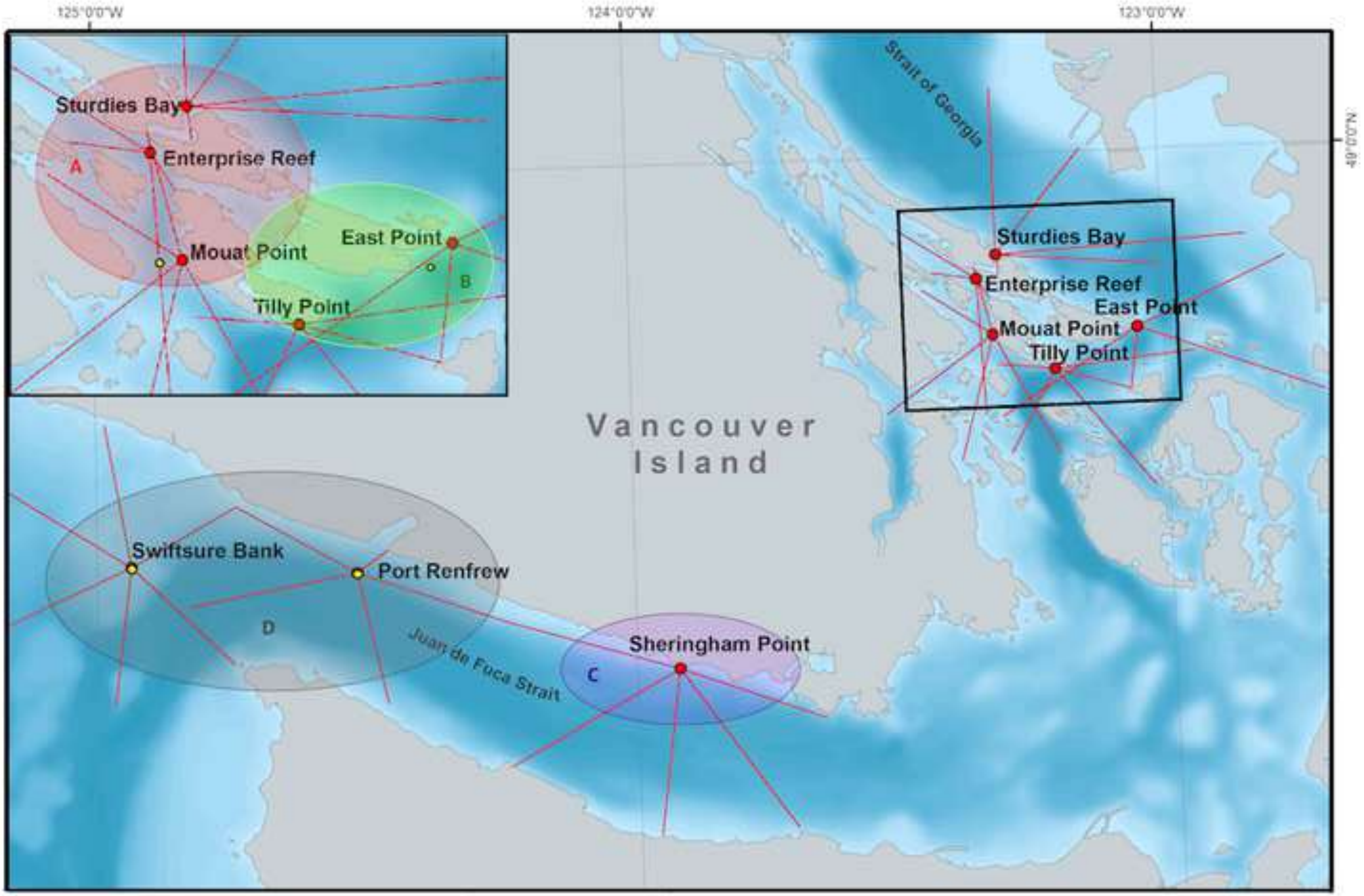

Fig 5

Click here to access/download;Figure;Fig5.tif

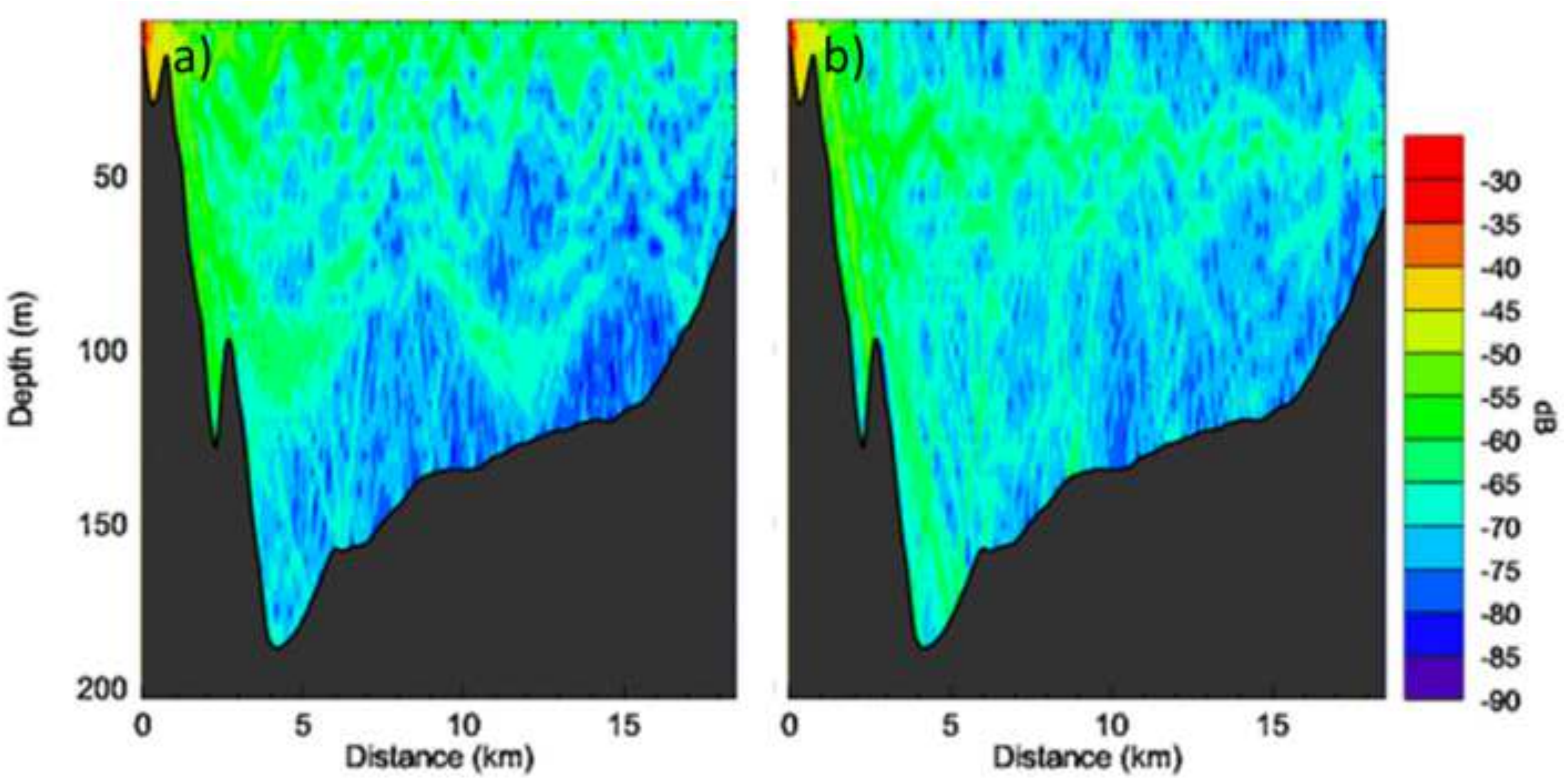

Fig 6

Click here to access/download;Figure;Fig6.tif

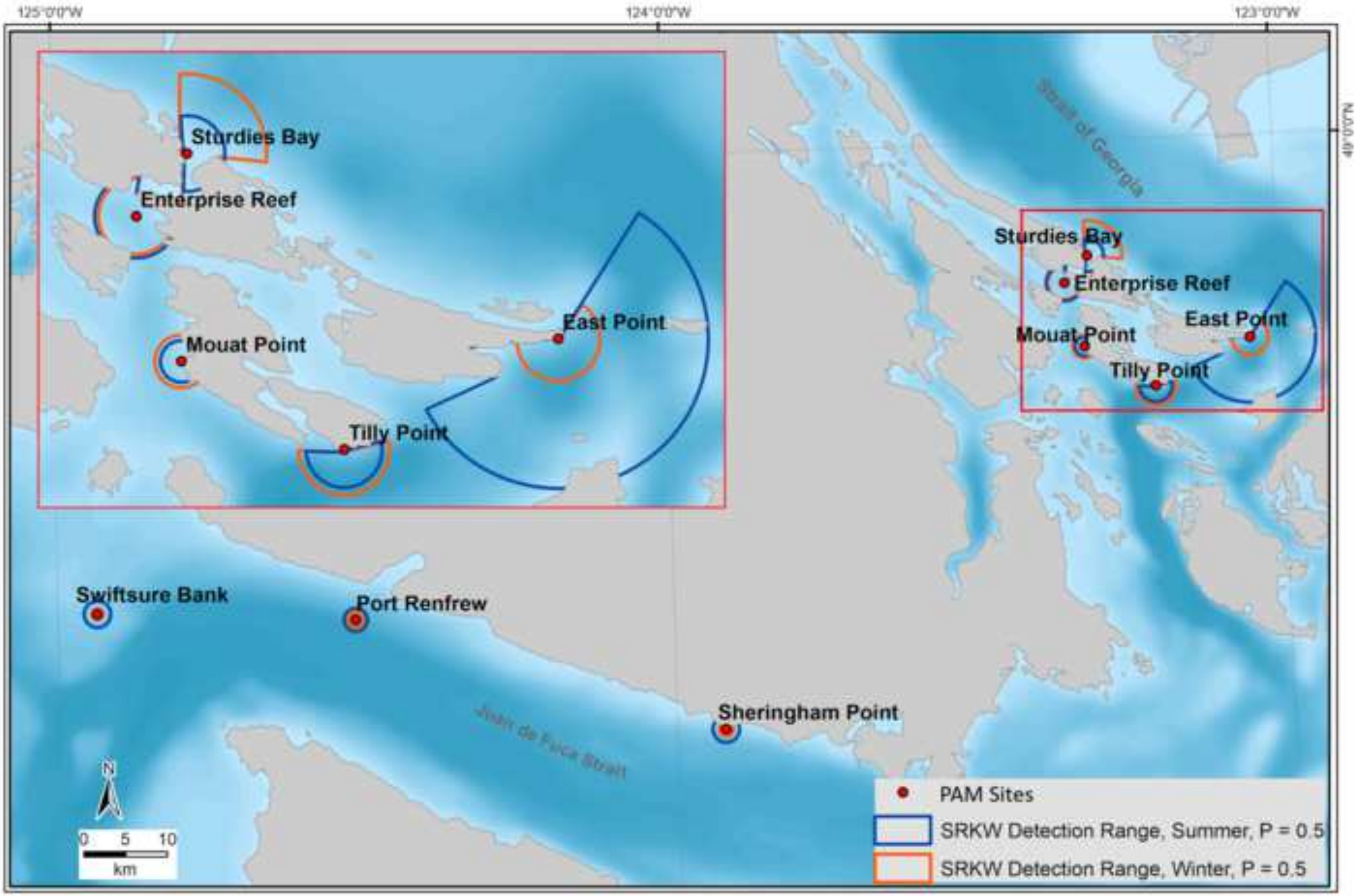

Fig 7

Click here to access/download;Figure;Fig7.tif

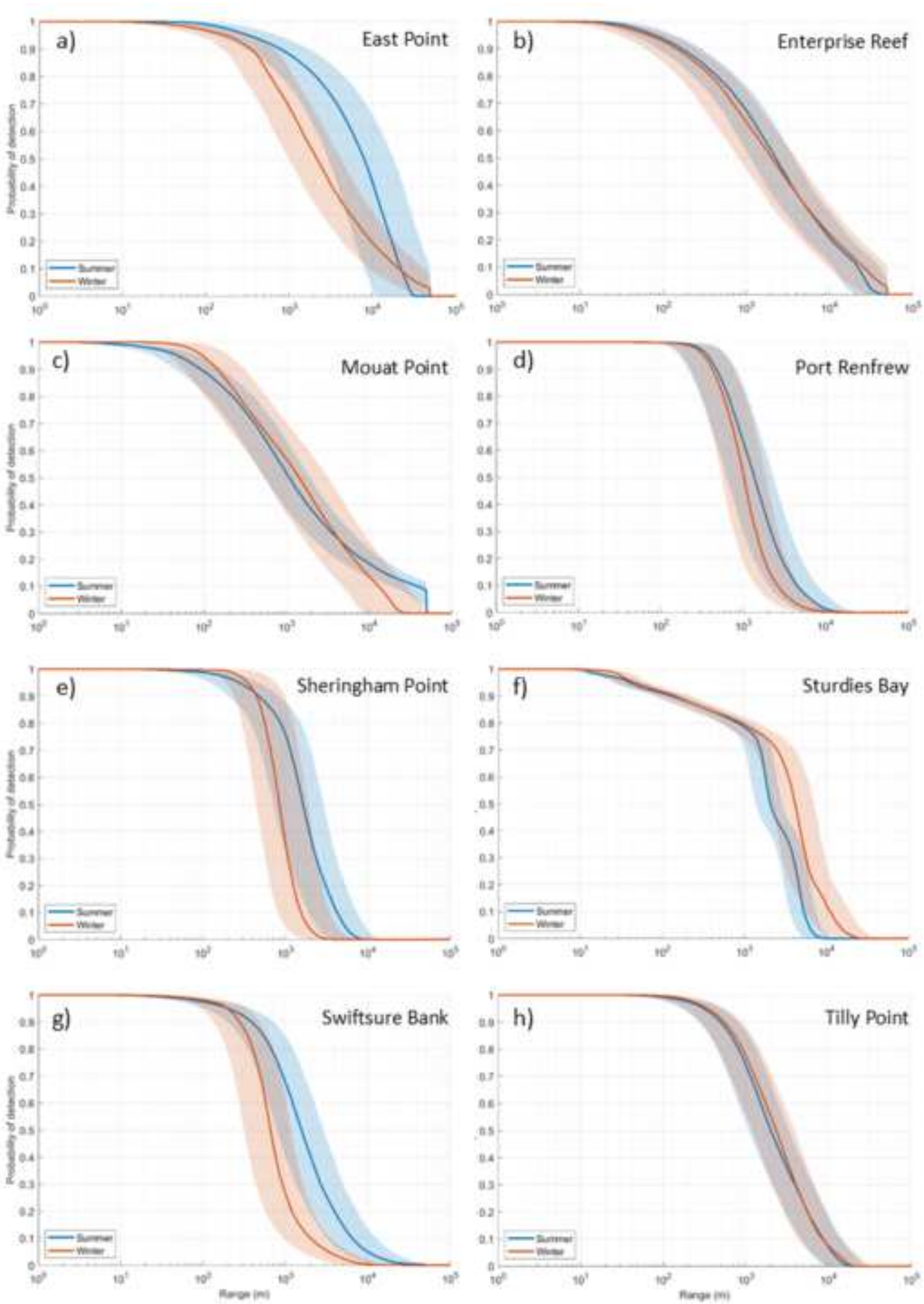

Fig 8

Click here to access/download;Figure;Fig8.tif

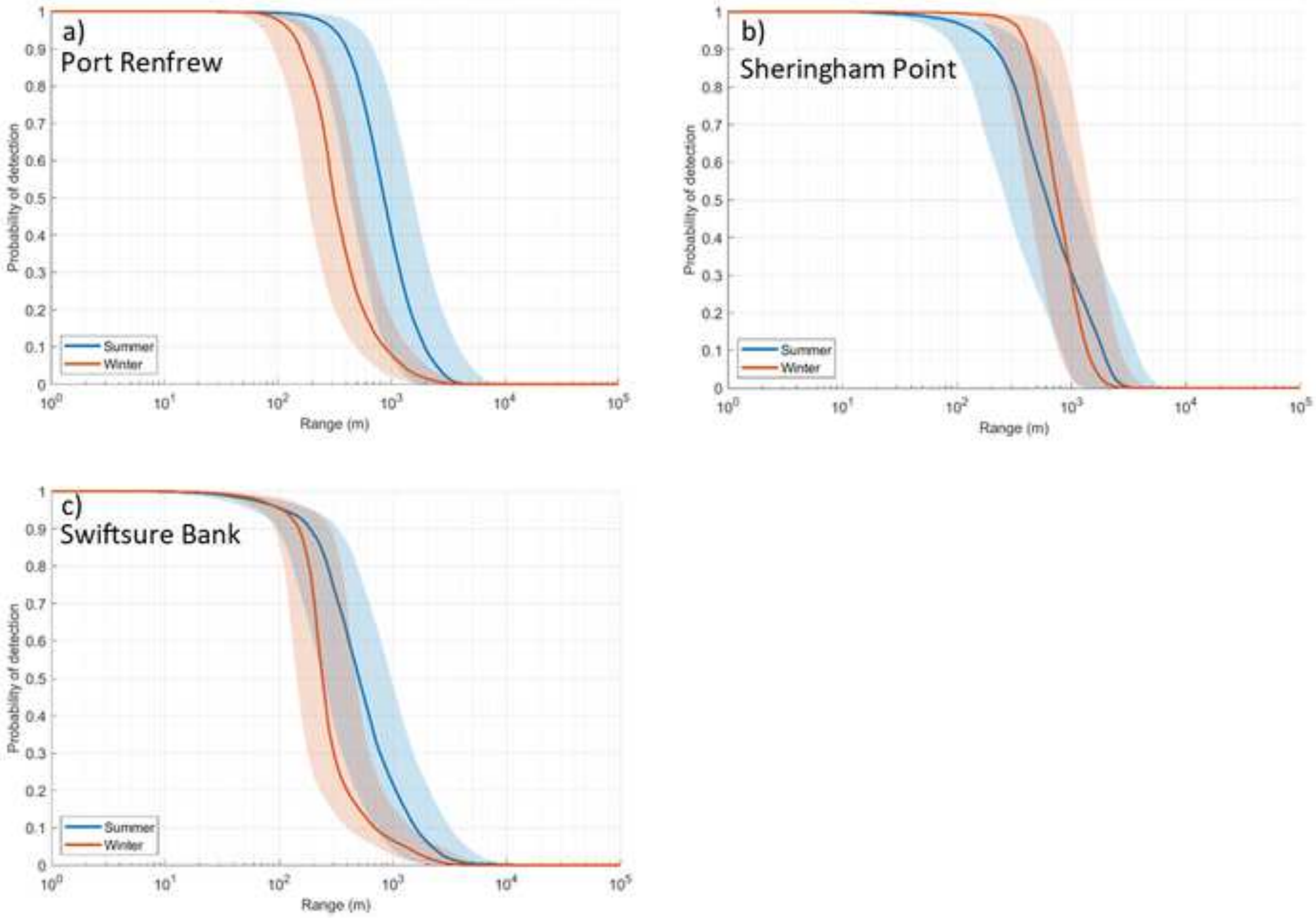

Fig 9

Click here to access/download;Figure;Fig9.tif

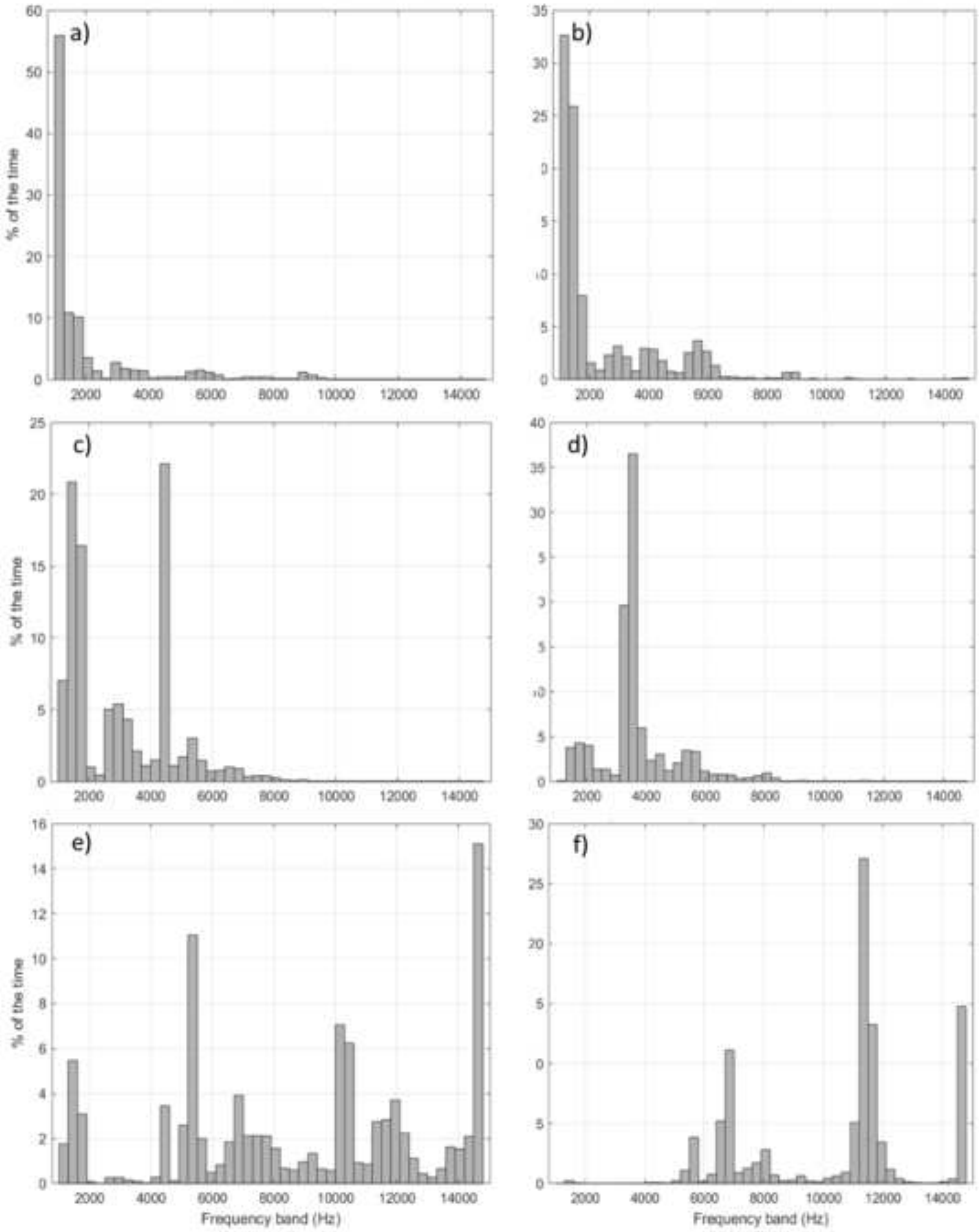

Fig 10

Click here to access/download;Figure;Fig10.tif

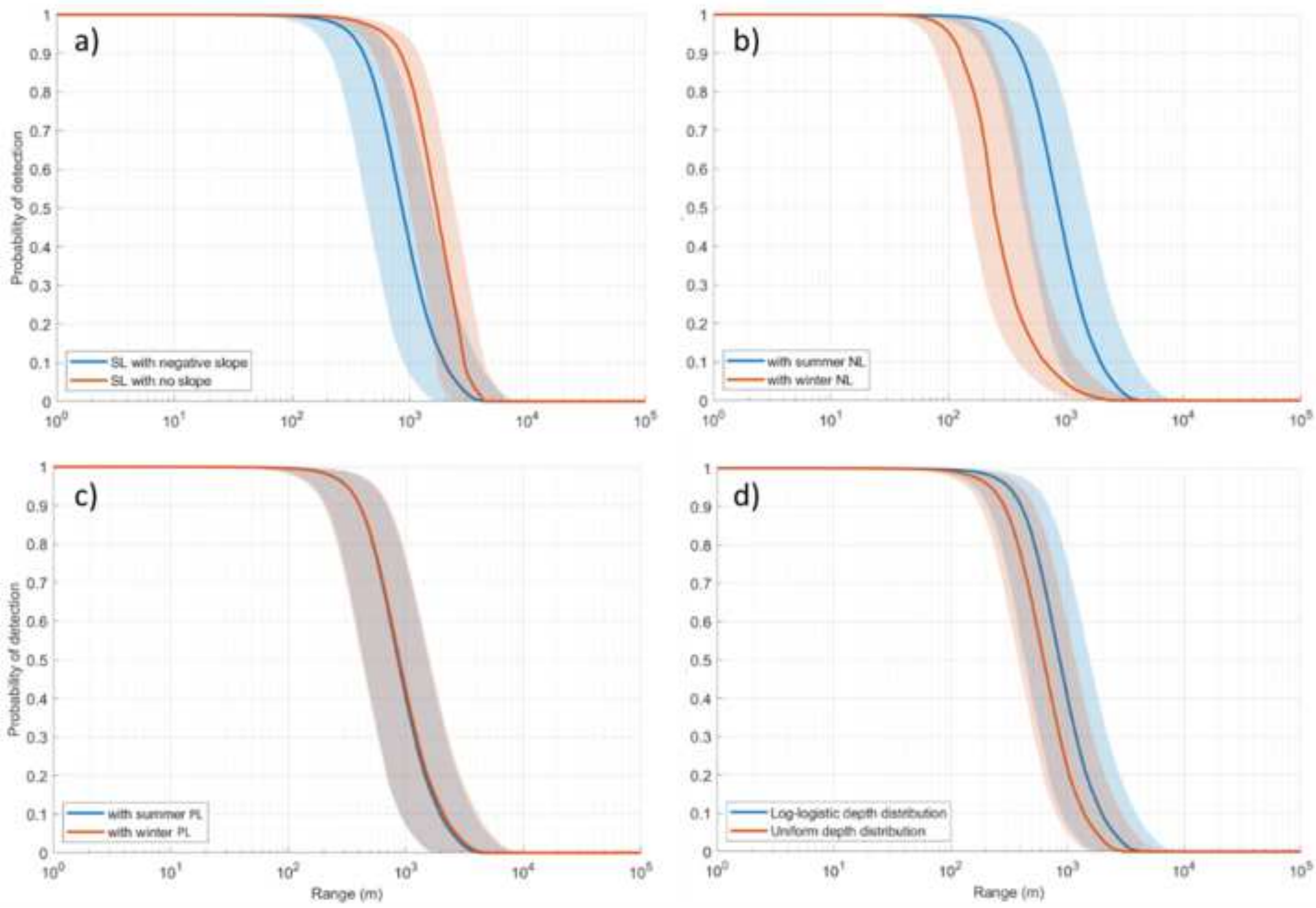

Fig 11

Click here to access/download;Figure;Fig11.tif

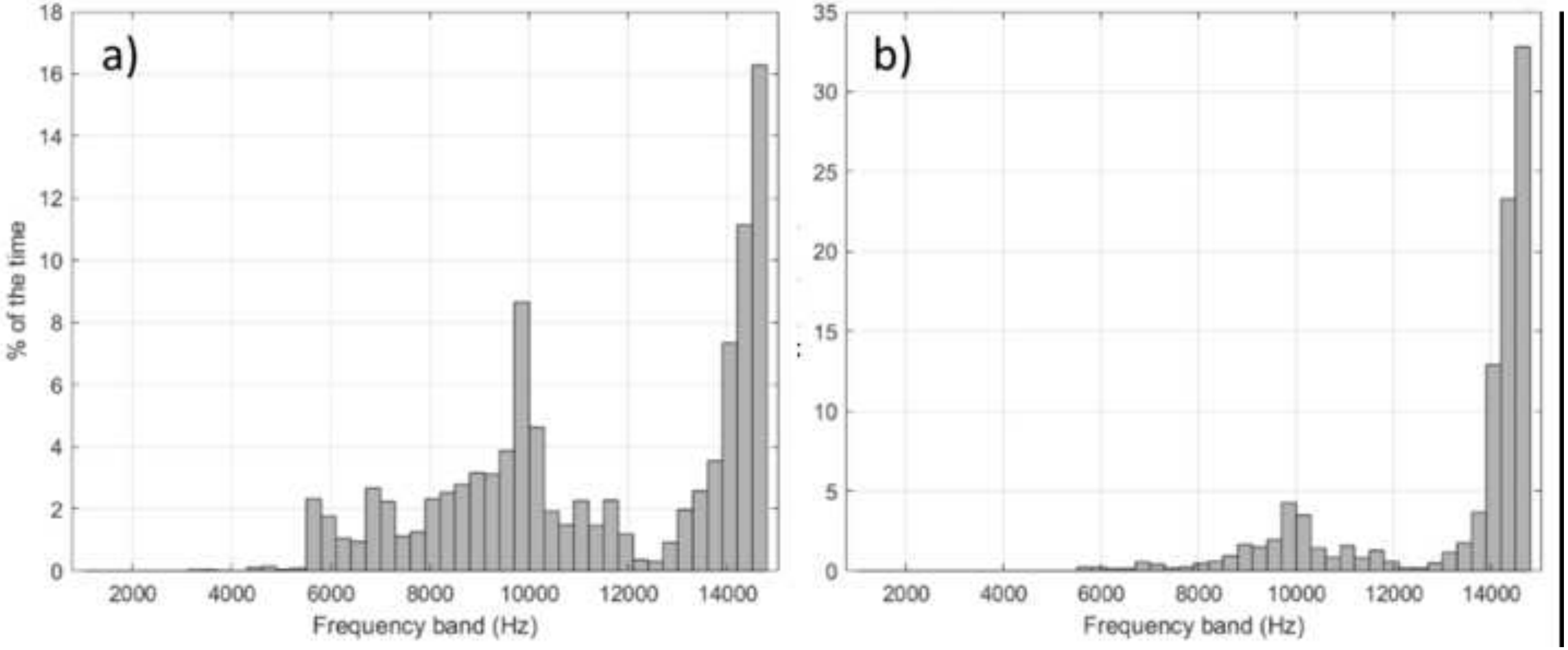

Fig 12 Click here to access/download;Figure;Fig12.tif

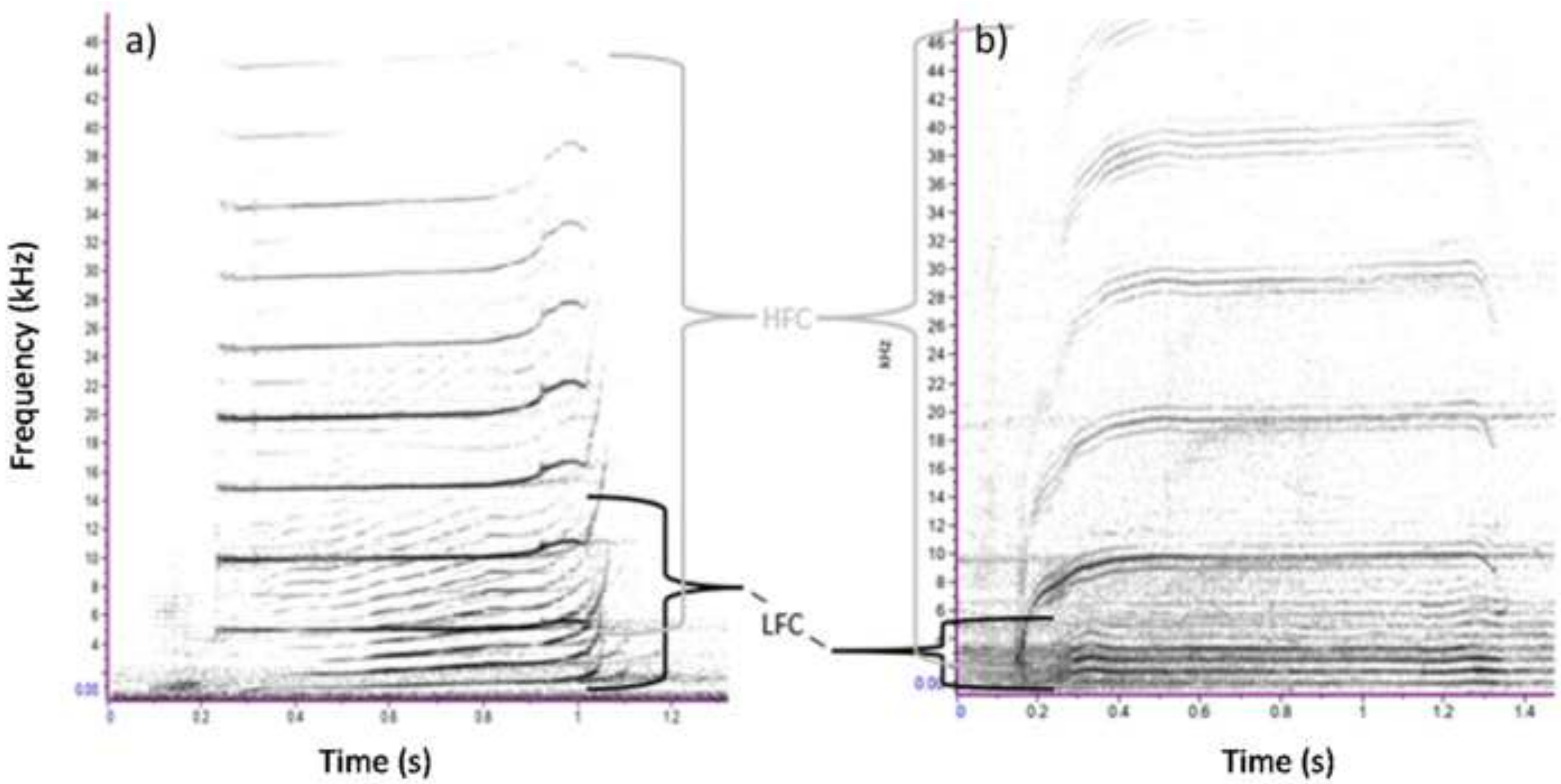

S1 Table

Click here to access/download
Supporting Information
S1 Table.docx

S2 Table

Click here to access/download
Supporting Information
S2 Table.tif

S3 Table

Click here to access/download
Supporting Information
S3 Table.docx

S4 Table

Click here to access/download
Supporting Information
S4 Table.tif

S1 Fig

Click here to access/download
Supporting Information
S1 Fig.tif

S2 Fig

Click here to access/download
Supporting Information
S2 Fig.tif

S3 Fig

Click here to access/download
Supporting Information
S3 Fig.tif

S4 Fig

Click here to access/download
Supporting Information
S4 Fig.tif

S5 Fig

Click here to access/download
Supporting Information
S5 Fig.tif

S6 Fig

Click here to access/download
Supporting Information
S6 Fig.tif

S7 Fig

Click here to access/download
Supporting Information
S7 Fig.tif

S8 Fig

Click here to access/download
Supporting Information
S8 Fig.tif

S9 Fig

Click here to access/download
Supporting Information
S9 Fig.tif

S10 Fig

Click here to access/download
Supporting Information
S10 Fig.tif

S11 Fig

Click here to access/download
Supporting Information
S11 Fig.tif

S12 Fig

Click here to access/download
Supporting Information
S12 Fig.tif

S13 Fig

Click here to access/download
Supporting Information
S13 Fig.tif

S14 Fig

Click here to access/download
Supporting Information
S14 Fig.tif

S15 Fig

Click here to access/download
Supporting Information
S15 Fig.tif

S16 Fig

Click here to access/download
Supporting Information
S16 Fig.tif

S17 Fig

Click here to access/download
Supporting Information
S17 Fig.tif
