# Supplementary material for: Modeling the detection range of pulsed calls from resident killer whale in nearshore waters of British Columbia, Canada
Source: PLoS One. 2025 Sep 26;20(9):e0331942. doi: 10.1371/journal.pone.0331942 (PMC12469096; doi:10.1371/journal.pone.0331942)
Supplement: Attachment — Submitted filename: Response to Reviewers.docx [file pone.0331942.s024.docx]

Response to Reviewers for Manuscript PONE-D-24-60689
Journal: PLOS ONE
Authors: Xavier Mouy, Melanie Austin, Jennifer Wladichuk, Harald Yurk

Date: 14 June 2025

Dear Dr. Vitor Hugo Rodrigues Paiva

We would like to thank the reviewers and editor for their thoughtful and constructive feedback, which has significantly helped improve our manuscript. Below, we provide detailed responses to each of the reviewers’ comments. All changes made in the manuscript have been noted with tracked changes and we indicate line numbers where relevant. You will find below the detailed response to each of the reviewers’ comments.

We apologize for including the funding information in the Acknowledgments Section of the manuscript. Funding-related text has now been removed from the manuscript.

The Funding Statement should read: “This study was funded by Fisheries and Oceans Canada and JASCO Applied Sciences. Xavier Mouy also received support from the Investment in Science Fund at the Woods Hole Oceanographic Institution”.

The Role of Funder statement should read “The authors are part of the organizations JASCO Applied Sciences and Fisheries and Oceans Canada which funded this work and consequently participated to the study design, analysis, preparation of the manuscript, and decision to publish. The Woods Hole Oceanographic Institution had no role in study design, data collection and analysis, decision to publish, or preparation of the manuscript”

Best Regards
Xavier Mouy, on behalf of all co-authors

Response to reviewers

1. **Reviewer #1**:
   1.1. Methods need “…a **more detailed description of how high SNR calls** were annotated, e.g. individual calls, call bouts, any buffer before and after call, etc. and state explicitly over what time domain the SL calculation was done.”
        1.1.1. Authors’ response:
               We have added the following information to the Source Level Estimation section L165:
               “All calls used in this study were annotated by bioacousticians specialized in killer whale acoustics with extensive experience in identifying call types and defining call type repertoires (Ford 1991, Yurk 2002, Pilkington et al. 2023) using spectrogram and sound simultaneously. Calls were annotated as single calls and call bouts were used to identify a specific killer whale population (S1 Fig) and killer whale presence was confirmed via photo identification of individuals and groups (matrilines and pods)(Bigg 1982).”

We also added examples of manual annotations in Figure S1 Fig.

1.2. Methods need “A clearer description of the **depth distribution data** used, e.g. what type of tag was used, were these calling depths or just general depth ranges?”
     1.2.1. Authors’ response:
            The depth distribution was achieved using Dtags (Johnson and Tyack 2003) and estimated based on attached tags to individual resident killer whales (*Orcinus orca ater*) (Wright et al. 2017). Wright and colleagues deployed 34 archival Dtags on 32 individual killer whales from the Northern Resident population. All deployments took place between Northern Vancouver Island and mainland British Columbia. See map below from Wright et al. 2017

We added the following text in the Vocalization Depth section L 192:
“This was accounted for in the Monte Carlo process by repeating the detection range calculations for 100 source depths randomly selected from a distribution representing the typical depth distribution of SRKW, modelled using *in situ* data collected by Wright et al. (2017) from 34 archival Dtags (Johnson and Tyack 2003) attached to 32 individual Northern Resident killer whales located between Northern Vancouver Island and mainland British Columbia.”

1.3. Methods need “Additional discussion on whether the **source level distributions** account for **directional biases** in killer whale vocalizations.” + “Address potential gaps in source level distribution assumptions and their implications on detection range estimates.”
     1.3.1. Authors’ response:
            Directionality was not considered during this study because source levels were estimated based on received sound levels recorded with single hydrophones and limited knowledge of the direction of the incoming signals. The source level estimation in our study uses linear curve fitting to the measured received levels of recorded calls and represent a source level decay with increasing frequency that better reflects the decay of source levels of the omnidirectional killer whale call components. More details are provided in response to comments by reviewer #2 regarding directionality.
1.4. “Suggest that Figure 9 have standardized y-axes on all plots and increased resolution.”
     1.4.1. Authors’ response:
            We recreated Figure 9 with standardized y-axes. The resolution of the figure was increased.
1.5. “Strengthen the discussion by explicitly **comparing detection range results** with those from other studies employing different methodologies (e.g., empirical validation vs. model-based approaches)."
     1.5.1. Authors’ response:
            We added the following text in the introduction (L82) to better set the problem our study is addressing:

“Most published cetacean acoustic detection range studies focus on baleen whales calls, which with the exception of those producing elaborate songs (e.g. humpback and bowhead whales) tend to have the majority of the call energy within a narrow frequency band (e.g., blue, fin, right whales; Stafford et al. 2007, Samaran et al. 2010). This tends to make it easier to measure source levels of calls and determine propagation loss in the narrow frequency band in which the call energy is located. In contrast, many odontocete calls (e.g., killer and pilot whale calls, beluga whale calls and burst pulses of smaller dolphins) and humpback whale songs are broadband signals whose energy is spread out over a wider frequency band. It appears more difficult to determine detection ranges of broadband vocalizations due to the more complex frequency dependence of propagation losses, source levels, and interfering noise levels.”

We also added the text below in the discussion (L400) to discuss comparison with studies employing different methodologies:

“Many studies estimating the acoustic detection ranges of baleen whale calls, such blue, fin and right whales use source level, background noise levels, and propagation losses estimated over a very narrow frequency band (42, 46). Detection range estimates become more challenging when examining broadband sounds such as killer whale pulse calls. Miller (2006) has estimated the detection range of resident killer whales in British Columbia by conducting acoustic localization to measure source level in third-octave bands and using a simplified propagation loss equation. While this study provided insights on killer whale communication range it did not take into account how the animal vocalization depth and seasonal water column properties (e.g. changes in sound speed profiles) affected the propagation loss and approximated noise condition based on sea-state rather than in-situ measurements. Such effects are important to understand and account for, particularly in the context of noise mitigation. Our approach, based on Monte Carlo simulations (Metropolis and Ulam, 1949), took into account the variability of the propagation losses with seasons, animal depths and frequency, the variability of the interfering background noise, and the frequency dependent variability in source levels. Other studies based on Monte Carlo simulations and acoustic propagation modeling have estimated detection probabilities for beaked, humpback whale and delphinid sounds (Küsel et al., 2011; Helble et al., 2013b; Hildebrand et al., 2015, Frasier et al., 2016). The approach used in this study similarly relies on Monte Carlo simulations but does not account for directionality, as the low-frequency components of killer whale calls that propagate over longer distances are not directional. Modelling studies such as Frasier et al. (2016) have shown good agreement between in situ detection results and model predictions which suggests that simulation studies can provide reliable detection range estimates. Comparing our simulation results with field measurements is a goal for future efforts.”

1.6. The manuscript would benefit from “additional text and plot in the results to show probability of detection under different ambient noise levels.”
     1.6.1. Authors’ response:

To illustrate the probability of detection under different ambient noise levels we added a figure (now Fig.12) that shows the probability of detection at East Point during the summer under low, medium and high ambient noise conditions.

**Fig 12. Detection range probabilities for SRKW pulsed calls in summer at East Point calculated separately for high (blue), medium (red), and low (orange) ambient noise conditions. The solid lines are the median values, and the shaded areas define the 25th and 75th percentiles.**

We also added this accompanying text at the end of the Results section:

“In order to better illustrate the effect of ambient noise levels on the acoustic detection range, the probability of acoustic detection of SRKW at East Point during the summer (Fig.7a, blue curve) was recomputed separately for high, medium and low ambient noise conditions (Fig. 12). High conditions correspond to broadband levels > 94.06 dB re 1 µPa, medium to broadband levels between 87.47 and 94.06 dB re 1 µPa, and low to broadband levels < 87.47 dB re 1 µPa. SPL intervals for each condition were defined by the 33rd and 66th percentile of broadband value in order to have each condition represented equally (14,348 1-minute recordings). Fig.12 shows that for a same location and season the fluctuation of ambient noise levels substantially impact the acoustic detection range going from a median detection range of 1640 m in the worst ambient noise conditions (high ambient noise conditions) to 15,500 m in the best conditions (low ambient noise conditions) for P = 0.5.”

1.7. “Add more text on how **Lombard effects** (e.g. if killer whales change their call amplitudes in response to noise) could affect detection range estimates. Presumably this could be simulated and while I wouldn’t request that for this study it could be noted as a future direction for the work.”
     1.7.1. Authors’ response:

We added the following text to the discussion (L443):

“This effect was not considered in the present study, as the Lombard response reported by Holt et al. was based solely on broadband source levels. In contrast, detection ranges in this study were estimated using variation in source levels across the 500 Hz to 15 kHz band, in 300 Hz steps. No information is currently available regarding Lombard responses at this finer 300 Hz band resolution, and assuming an evenly distributed Lombard response across the modeled frequency range is unlikely, given the sound production mechanisms in odontocetes (Madsen et al. 2023). Incorporating this effect to the detection range modeling is possible but would require a more comprehensive characterization (e.g., more call samples from multiple locations and a better understanding of the frequency dependence of the response) to benefit the detection range estimates.”

1.8. “Discuss whether **directional characteristics of killer whale calls** might impact estimated detection probabilities”.
     1.8.1. Authors’ response:
            Directional call characteristics likely influence detection probabilities, but these effects are frequency hence range dependent. As indicated in L436, only the high frequency parts of the calls are directional and do not propagate far. The low frequency parts of the calls are omnidirectional and propagate further than the high frequency components. The low frequency components of the calls are responsible for the maximum acoustic detection ranges that we estimate and therefore are thought to be minimally impacted by the directionality of the calls. This is discussed further in the responses to Reviewer #2.

We added the text below at L439 to clarify:

“Directional HF call components, however, attenuate over shorter distances than LF components. Maximum detection range may therefore be primarily influenced by the range of omnidirectional LF call components.”

1.9. “Add discussion of how this approach may be used in **Density Estimation from PAM**. This may also be a useful addition to the Introduction” + “The authors miss an opportunity to discuss how this approach could be used in Density Estimation from PAM recorders given we know cue counts (from tag data).”
     1.9.1. Authors’ response:
            While this is a worthwhile goal and should be addressed in future study, we feel that this study does not provide enough relevant information on the subject of density estimations using PAM, because it focusses on the detection ranges of single calls. Killer whales like many other delphinids are highly vociferous and a study on density estimation should focus on that aspect of the vocal behavior.
1.10. Minor text edits and comments can be found on the attached document.
      1.10.1. Authors’ response:

All text edits and comments in the pdf documents have been addressed.

2. **Reviewer #2:**
   2.1. “My main issue with the paper is that that the authors are not calculating the probability of detection. They are calculating the probability of a detecting a killer whale pulsed call for a killer whale that happens to be facing the hydrophone at a mean source level over a time series of noise. In the paper, the authors are often careful to point out that they are measuring maximum detection range but regularly use terms like “probability of detection” that would generally be associated with a true detection function used in distance sampling. The problem with measuring maximum range is that it is not particularly useful because at that range you will always miss most animals unless they produce sounds that are omni-directional. In this case, the authors are measuring killer whale pulsed calls which are produced via the same mechanism as clicks and are thought to be directional, especially at higher frequencies which are included in the simulation (Miller et al, 2002).”
        2.1.1. Authors’ response:

The reviewer’s statement that our study uses mean source level is not accurate: our approach uses many realizations of source levels from the distribution reported in the literature (see Table 1). The reviewer's comment that the study/model only considers source levels of directional call components is not supported by the methodological approach taken and the published evidence on killer whale call directionality. Presumably, the reviewer refers to the Miller (2002) publication describing the differences in directionality of call components to support the comment that our study only calculates the *“the probability of a detecting a killer whale pulsed call for a killer whale that happens to be facing the hydrophone at a mean source level over a time series of noise”* The paper in question clearly shows that calls have both directional and omnidirectional components (Miller 2002, Fig. 3) by describing the spectrograms of received call sound energy differences when the whales face the receiver (hydrophone array 200m in front of the whales) versus when the whales face away from the receiver (hydrophone array 500m behind the whales). See Figure below.

Killer whales like a number of other odontocetes produce two-voiced or bi-phonic calls (Miller et al. 2007, Filatova et al. 2015) with the two call components often showing distinct acoustic variation (Filatova 2020) as shown in Figure 12 of the manuscript. The resulting effects on call propagation is that the low frequency (LF) component propagates omnidirectionally while the high frequency (HF) component propagates directionally. As input for the detection range modelling used in our study, we assumed a linear decay of sound energy with increasing frequency (red line in Figure 2 c and d) and below).

This methodological approach favors the influence of the LF (omnidirectional) component on the detection range estimate as supported by the findings published by Miller in 2002 (Fig. 4 – upper graph):

The influence of the HF component is stronger in the source level estimates of Northern Resident Killer Whales (Fig. 2d) which may have been the result of a call sampling bias and has been discussed in line 455 to 469 of the manuscript. We added more details to the discussion to clarify that in the re-submitted version.

Miller, P. J. (2002). Mixed-directionality of killer whale stereotyped calls: a direction of movement cue?. Behavioral Ecology and Sociobiology, 52, 262-270.

Miller, P. J., Samarra, F. I., & Perthuison, A. D. (2007). Caller sex and orientation influence spectral characteristics of “two-voice” stereotyped calls produced by free-ranging killer whales. *The Journal of the Acoustical Society of America*, *121*(6), 3932-3937.

Filatova, O. A., Samarra, F. I., Deecke, V. B., Ford, J. K., Miller, P. J., & Yurk, H. (2015). Cultural evolution of killer whale calls: background, mechanisms and consequences. *Behaviour*, *152*(15), 2001-2038.

Filatova, O. A. (2020). Independent acoustic variation of the higher-and lower-frequency components of biphonic calls can facilitate call recognition and social affiliation in killer whales. *PloS one*, *15*(7), e0236749.

2.2. “The authors use equation 3 (which is incorrect, but I assume you used correctly in the simulations)”
     2.2.1. Authors’ response:
            Thank you for noticing this error in the manuscript. Equation 3 has been corrected in the manuscript.
2.3. “They then use a Monte Carlo simulation to calculate the uncertainty in the maximum range which they term the probability of detection with range for calls. This is not the probability of detection as the term is generally used, it is the probability of receiving a call at the mean source level for a killer whale facing the hydrophone. However, the authors have done all the hard work in getting together good quality inputs for a true probability of detection simulation and so it seems a shame to not take this further.
     2.3.1. Authors’ response:
            The model estimates the acoustic detection ranges of single calls and not the probability of acoustic detection of killer whales. The goal of the study was to determine the variation in detection ranges of single calls to simulate the ability of automated acoustic detection as a whale tracking tool for harm mitigation. The detection distance of a single call denotes the onset or longest distance of detectability by an automated detection system, which is relevant for assessing the effectiveness of acoustic tracking of killer whales through an area. The model would need to be adapted to be useful for other purposes. We added clarifying text about the goal in the re-submitted version of the manuscript.
2.4. “My advice would be to redo the analysis and use a Monte Carlo approach to simulate the probability of detecting a whale as in (e.g. Frasier et al, 2016). These methods would allow directionality to be incorporated and utilize all the very good work in this paper on propagation, noise level estimation and explore the frequency dependence of source levels. “Note that the probability of detection simulation approach uses distributions like source levels, depth and vertical angle (which you can get from tag data) along with beam profile to calculate a probability of detecting a sound with range. The uncertainty in the inputs and input distributions (rather than the distributions themselves) is used to calculate an error in the simulation rather than what the authors have done which is to take empirical distributions of source levels etc. and use them to calculate an uncertainty in the maximum detection range with distance. The probability of detection can be mapped to noise levels and then an average probability of detection over a time series of noise calculated. This can be used to determine an effective detection radius which can then be plotted on Figure 6 along with maximum detection range. This would make the paper much more useful and prevent the misuse of results (i.e. by folk who think this represent the probability of detecting a killer whale).”
     2.4.1. Authors’ response:
            Incorporating directionality requires fine scale data on the influence of directionality on killer whale call sources levels across the selected frequency range of 1-15 kHz. The existing empiric data on call source level distribution across this frequency range did not allow to distinguish which calls were directed towards the hydrophone and which not, especially for the LF component of calls. The model prediction favors the propagation of the omnidirectional LF component of calls because this component generally transmits further while the HF component attenuates faster. Because the purpose of the study was not to estimate the probability of detecting a killer whale at a specific location but determining the ability of acoustic detection to track killer whales, we are confident that the right methodology was applied.
2.5. “More generally it would be good in the results section to provide some plots showing time long term time series of the change in maximum range and also to explore which frequency bands are resulting in the maximum ranges as I imagine higher frequencies have higher absorption, but noise is higher at lower frequencies. Is there a portion of the call that is usually detected more easily or is this quite dependent on environmental factors?– that isn’t clear but all the work has been done from equation 4.”
     2.5.1. Authors’ response:
            We don’t believe that showing a time series of maximum detection ranges would help the clarity of the manuscript. However, we do agree that more explicitly showing the effect of ambient noise levels on the maximum detection ranges would be helpful. To that effect, we added a paragraph and a new figure at the end of the Results section to show detection ranges in different ambient noise levels conditions (low, medium, high). See section 1.6.1 of this document for further details.

Understanding which frequencies are responsible for the maximum detection ranges is an important part of our analysis that is already presented and discussed throughout the manuscript. Figures 9, 11, S11 and S12 show which frequency bands were responsible for the maximum detection range for each site, population (SRKW, NRKW) and season modelled in this study. Discussion on this aspect of our results can also be found in the paragraphs starting at L433 and L462.

2.6. “What I am proposing (and hoping for) is a significant rewrite and re-analysis and as such this constitutes major revisions. I would be happy to review the paper again if this were done, however, I understand that this may not be possible and there is still value in what has been done here. If the authors rejected these suggestions, then this version of the paper could be published if the specific issues below are rectified. Particularly, there needs to be careful attention to ensure that there is no ambiguity about what is termed “**probability of detection**” and some of the above points need to be added to the discussion.”
     2.6.1. Authors’ response:
            We have addressed the comments by clarifying the goal of the study and the underlying methodology. We do not believe that a re-analysis of the same data will produce results that are fundamentally different from the ones we presented because directionality was not considered and is only relevant to the propagation of HF parts of the calls that attenuate much faster than the LF parts. Killer whale calls are not characterized as completely directional or omnidirectional and components that are directional have less influence on the maximum detection range of single calls. We acknowledge that using sequences of calls increases the probability of representing various input angles and would improve the results. By using the complete known repertoire of discrete calls from at least one of the populations we are confident though that the mixture of different call types represents a relevant cross section of influences of both LF and HF components on the source level estimation. We changed “probability of detection” to “probability of acoustic detection” where it could have been ambiguous.
2.7. “I would also note that all figures are blurry in the pdf which I assume is not be the fault of the authors but is frustrating for a reviewer.”
     2.7.1. Authors’ response:

We are sorry to hear that figures were blurry on the reviewers version of the manuscript. We have verified our original image files and the resolution is fine. We will work with the journal to ensure the figures in the final manuscript are not blurry.

2.8. “Line 54 and it provides information when animals are underwater.”
     2.8.1. Authors’ response:
            We added this information to L54: “Compared to other monitoring methods such as visual observations, PAM has several advantages including that it can observe animal while they are underwater and is less affected by daylight, weather, and sea conditions.”
2.9. “Line 54 I know what you mean but PAM is not only useful for animal sounds – it is useful for a whole host of different things – noise etc. Would reword.”
     2.9.1. Authors’ response:

We acknowledge that PAM can serve a variety of purposes; however, in this manuscript, we deliberately focus on its use for monitoring marine fauna. We prefer not to revise this sentence, as it reflects the intended scope of the study.

2.10. “Line 70 Sudden switch from passive to active text. Would keep it consistent.”
      2.10.1. Authors’ response:

Thank you for the observation regarding the shift from passive to active voice. We reviewed the passage carefully and have opted to retain the current phrasing, as it reads clearly and aligns with the tone and style of the rest of the manuscript.

2.11. “Line 100. Does this account for absorption? That needs to be mentioned.”
      2.11.1. Authors’ response:

We agree that it is an important piece of information that we had initially omitted. We edited this sentence to indicate that the absorption is taken into account (L110):

“We calculated PL using a specialized acoustic model that integrates parabolic equation and ray tracing methods (MONM; JASCO Applied Sciences, Ltd.) and accounts for source depth, receiver depth, and environmental and bathymetric conditions between the source and receiver, and absorption”

2.12. “Line 106 – yeah but it’s an arbitrary threshold anyway so mentioning processing gains seems a little arbitrary? Would maybe just remove that.”
      2.12.1. Authors’ response:

We agree and removed this sentence.

2.13. “Line 110 Equation is wrong. In Eq 1 you have used PL as a transmission loss and it has units of dB re 1 m2. In eq 3 you have used PL as a coefficient i.e TL = PL*log10(R). The coefficient is dimensionless. Also, you have described PL in Eq 6 which is inconsistent with equation 4.”
      2.13.1. Authors’ response:
              Thank you for catching this. Equation 3 has been corrected to be consistent with this definition of PL. The text in question has been moved up in the document for clarity.
2.14. “Line 199 Equation 6 should be base ten log?”
      2.14.1. Authors’ response:

We have edited this equation (now numbered Eq. 2) to show “log_10_”

2.15. “Line 352 should maybe be average detection range (change throughout where appropriate)”
      2.15.1. Authors’ response:
              It should actually be the median detection range. We changed this through the manuscript where appropriate.
2.16. “Line 376 – so does this mean your icListen systems were not calibrated (not mentioned in methods)? If this is true, then you need to discuss somewhere why you think this not an issue – e.g. the manufacturer’s quoted sensitivities are known to be quite accurate. A SoundTrap sensitivity varies by 6dB which will have significant effects on your range estimation – you are not using these devices but icListen systems are similar…ish. If calibration is an issue then this should be added to the sensitivity analysis.”
      2.16.1. Authors’ response:
              Thank you for catching this. This sentence was not accurate as all systems were calibrated. We removed the mention to the sensitivity. This sentence now reads:
              “Differences in the mooring configurations, and recorder depths could result in differences between the summer and winter ambient conditions that are not strictly due to natural seasonal differences.”
2.17. “Line 439 No it is not a calculation of the probability of detecting a single vocalisation – see discussion above.”
      2.17.1. Authors’ response:

This comment has been discussed and addressed in previous responses to reviewers.

2.18. “Line 446 Yes, the language “detection range probabilities” is correct (probably should be “maximum detection range probabilities”) – use this above.”
      2.18.1. Authors’ response:
              This has been discussed and addressed in previous responses to reviewers.
2.19. “Line 466. Again, I don’t think maximum detection range is a particularly useful parameter (although frequently reported in ltrature) for any directional vocalization. Nice to know but not that useful for say density estimation. For example, if simulating the probability of detection, then your probability scales with whatever you set as the area you are monitoring over - and thus the area term in the density estimation equation cancels out. Similarly, many experimentally defined detection probabilities will have a maximum detection range quoted.”
      2.19.1. Authors’ response:
              This has been discussed and addressed in previous responses to reviewers.
2.20. “Line 472. Remove “probability of detection”.
      2.20.1. Authors’ response:
              This sentence has been reworded based on the suggestions from reviewers 2 made in the pdf document.
2.21. Reviewer #2 replied “No” to the question “Have the authors made all data underlying the findings in their manuscript fully available?”
      2.21.1. Authors’ response:

All the underlying data used for this study were uploaded to the OSF repository located here:

<https://osf.io/6ctjq/files/osfstorage?view_only=f4e6e83b9eab427bb530a74638ba3e52>
