# Supplementary material for: Modeling the detection range of pulsed calls from resident killer whale in nearshore waters of British Columbia, Canada
Source: PLoS One. 2025 Sep 26;20(9):e0331942. doi: 10.1371/journal.pone.0331942 (PMC12469096; doi:10.1371/journal.pone.0331942)
Supplement: Attachment — Submitted filename: Response_to_Reviewers_auresp_2.docx [file pone.0331942.s025.docx]

Response to Reviewers for Manuscript PONE-D-24-60689_R1
Journal: PLOS ONE
Authors: Xavier Mouy, Melanie Austin, Jennifer Wladichuk, Harald Yurk

Date: 16 August 2025

Dear Dr. Vitor Hugo Rodrigues Paiva

We would like to thank Reviewer #2 for agreeing to review our manuscript again and for providing thorough and constructive comments. We are also grateful to Reviewers #3 and #4 for bringing a fresh perspective to the manuscript and offering insightful suggestions. We have addressed all comments and hope that this version will meet the expectations of the editor and all reviewers. Below, we provide detailed responses to each comment. All changes made in the manuscript are marked with tracked changes, and we indicate line numbers where relevant.

Best Regards
Xavier Mouy, on behalf of all co-authors

Response to reviewers

1. **Reviewer #2**:
   1.1. the authors have decided to move to the terminology “probability of acoustic detection” which really should be “Probability of possibility of acoustic detection” (has a nice ring to it right?). I think that using “probability of on-axis detection” is probably a good compromise (even though in the LF case you will be looking at close to a true probability of detection) and I would like to see this in all plots and all references to the probability of detection or similar. For example, statements like L537 need to be changed
        1.1.1. Authors’ response:

We have opted to use “probability of on-axis detection” throughout the manuscript (in the text, figures and tables) as suggested by reviewer #2.

We also added the following in the “Methods - Approach” section:

L150: “While the low-frequency components of killer whale pulsed calls are omnidirectional, the higher-frequency components exhibit some degree of directionality (Miller 2002). In most cases, the low-frequency components determine the maximum detection range, allowing us to estimate the true probability of detection. In some instances, however, the higher-frequency components define the maximum detection range. In these latter cases, because directionality is not incorporated into the Monte Carlo simulation, only the probability of on-axis detection can be estimated. To prevent any potential misinterpretation, we therefore report all results as probabilities of on-axis detection*.*”

We added this sentence to the conclusion:

L547: “In most cases, the omnidirectional low-frequency components determine the maximum detection range, allowing us to estimate the true probability of detection. In some cases, however, the more directional high-frequency components set the maximum detection range, in which case the estimated probability of detection applies only to animals on-axis.”

We also updated axes labels in figures Fig 7, 8, 10, 12 with “probability of on-axis detection”

1.2. “The approach used in this paper is designed to compute the probability of detecting a single vocalizing whale” needs to be changed to “The approach used in this paper is designed to compute the maximum detection range for detecting a single vocalising whale.”
     1.2.1. Authors’ response:

This was changed.

1.3. “The approach presented shows the probability of detecting killer whale

calls at each location” needs to be changed to “The approach presented shows the probability of detecting killer whale on-axis calls at each location”.

1.3.1. Authors’ response:

We opted not to change this sentence but added the following sentence, right after to address the reviewer’s comment:

L548: “In most cases, the omnidirectional low-frequency components determine the maximum detection range, allowing us to estimate the true probability of detection. In some cases, however, the more directional high-frequency components set the maximum detection range, in which case the estimated probability of detection applies only to animals on-axis.”

1.4. Finally, both reviewers asked for further discussion of density estimation. Whilst I am happy for push back on some of the points responded to, I think the paper has to have a paragraph on this. In particular, the authors need to discuss the points above about how the low frequencies will be close to a true probability of detection and the high frequency cases not so much.
     1.4.1. Authors’ response:

We added a paragraph at the end of the Discussion section:

L538: “While this was not the primary focus of this study, the probability of on-axis detection estimated here can provide valuable information for animal density estimation using passive acoustics (85) . As noted previously, the low-frequency components of killer whale pulsed calls are omnidirectional and generally determine the maximum detection range. This was the case for the vast majority of SRKW simulations we conducted (S12 Fig). In such cases, the estimated probability of on-axis detection is very close to the true probability of acoustic detection and could be used directly in density estimation studies employing distance sampling or Spatially Explicit Capture–Recapture approaches based on single calls. However, as we found (primarily for NRKW), the higher-frequency, more directional components of pulsed calls can sometimes set the maximum detection range (S13 Fig). In these cases, directionality patterns would need to be defined (either with in situ measurements or using modeling) and directionality would need to be incorporated into the analysis using methods similar to those described by (72) before the estimates could be used for density estimation.”

1.5. The author response to reviewer 1: “While this is a worthwhile goal and should be addressed in future study, we feel that this study does not provide enough relevant information on the subject of density estimations using PAM, because it focusses on the detection ranges of single calls. Killer whales like many other delphinids are highly vociferous and a study on density estimation should focus on that aspect of the vocal behavior.” I don’t think is adequate. Density estimation is often performed on single calls via distance sampling or SECR of which a probability of detection of a single call is a key component
     1.5.1. Authors’ response:

Thank you for this correction. This was taken into account in the paragraph we added in the discussion section (see comment above).

2. **Reviewer #3**:
   2.1. The last sentence of the abstract is rather vague make more informative how your findings help future studies also make more explicit in conclusion
        2.1.1. Authors’ response:

We modified the last sentence:

L28: “Although developed for killer whales, this approach can be adapted to other vocal species to quantify species-specific detection range probabilities, guide optimal hydrophone placement to maximize coverage in noisy environments (with application to noise impact mitigation strategies), and provide needed inputs for passive acoustic density estimation models.”

2.2. Discussion should be more concise and instructive
     2.2.1. Authors’ response:

While the discussion section constitutes a substantial portion of the manuscript, the simulation results contain a large amount of information that must be carefully unpacked to ensure they are accurately presented and interpreted. We believe the points addressed in this section are important and instructive, and reducing them substantially could compromise clarity. That said, we have added content on density estimation to provide additional context and enhance the instructive value of the discussion.

2.3. I think you should mention the main differences between towed and moored hydrophone surveys and also mention that its optimal to have visual surveys conducted simultaneously (cite -https://doi.org/10.1111/mms.13113)
     2.3.1. Authors’ response:

To maintain focus and limit the length of the manuscript, we have not included a detailed comparison between towed and moored hydrophone surveys, as our study specifically addresses moored static recorders. We have previously published studies demonstrating the benefits of concurrent visual and PAM surveys for studying killer whales in British Columbia (e.g., Burnham et al., 2016; Frouin-Mouy, Mouy et al., 2022). While this combined approach can be optimal, it is often not feasible for long or large-scale surveys due to the substantial effort, cost, and visibility constraints (e.g., weather, nighttime). We therefore chose not to expand on this topic here in order to keep the manuscript focused and concise.

References:

- Burnham, R.E., Palm, R.S., Duffus, D.A., Mouy, X., and Riera, A., 2016. “The combined use of visual and acoustic data collection techniques for winter killer whale (Orcinus orca) observations,” Global Ecology and Conservation 8, 24-30. <https://doi.org/10.1016/j.gecco.2016.08.001>.
- Frouin-Mouy, H.*, Mouy, X.*, Pilkington, J., Küsel, E., Nichol, L., Doniol-Valcroze, T., Lee, L., 2022. “Acoustic and visual cetacean surveys reveal year-round spatial and temporal distributions for multiple species in northern British Columbia, Canada,” Scientific Reports 12, 19272. https://doi.org/10.1038/s41598-022-22069-4.
  2.4. You should briefly outline the functional differences between clicks, whistles and pulsed calls for biologists who are not familiar with cetacean acoustics - PlosOne reaches a wide audience.
       2.4.1. Authors’ response:

The functional difference between clicks whistles and pulsed calls is explained in the introduction between lines 67 and 70:

“They produce a variety of sounds including echolocation clicks during foraging and navigation, as well as narrowband whistles and broadband pulsed calls used in social communication. Discrete pulsed calls are the most commonly heard vocalizations that occur in all behavioral contexts and can be distinguished aurally and sorted into distinct repertoires identifying different populations and social groups within populations (26).”

2.5. It would be good to include a paragraph in the discussion about future possibilities to use AI/ML tools to assist in Killer Whale Acoustic detection and mention advances in this field for other species i.e., <https://www.projectceti.org/>
     2.5.1. Authors’ response:

While AI/ML is a topic of great interest to us, we believe it falls outside the scope of this manuscript, which focuses specifically on detection range modeling. In our current work, the only input required from detectors is the definition of the detection threshold parameter, DT, in Eq. (3), which can be determined experimentally and is independent of the AI/ML methodology used. For this reason, we have chosen not to include an AI/ML discussion here in order to maintain a concise and focused scope.

3. **Reviewer #4**:
   3.1. Lines 64-67: It would be relevant to provide a bit more detail on the acoustic characteristics of the vocalizations mentioned (typical frequency ranges and durations).
        3.1.1. Authors’ response:

We have moved the sound description paragraph that was originally in the Methods section here:

“Clicks are short pulses that usually occur in a series. A click duration ranges from 0.1 to 25 ms (50). Whistles are single narrow-band tones with little harmonic structure in the 1.5–18 kHz frequency band. Their duration ranges from 50 ms to 12 s (50). Pulsed calls are harmonically structured with frequency ranges between 80 Hz and 40 kHz (51, 52).”

3.2. Line 91: You should specify the typical values for this narrow frequency band to provide more context.
     3.2.1. Authors’ response:

We edited this sentence:

L92 : Most published cetacean acoustic detection range studies focus on baleen whale calls, which, except for species that produce elaborate songs (e.g., humpback and bowhead whales), typically concentrate the majority of their call energy within a narrow frequency band of a few tens of hertz (e.g., blue, fin, right whales; (42, 46)).

3.3. Line 94: Same comment but for the wide frequency band.
     3.3.1. Authors’ response:

We edited this sentence:

L98: “In contrast, many odontocete calls (e.g., killer and pilot whale calls, beluga whale calls and burst pulses of smaller dolphins) and humpback whale songs are broadband signals whose energy is spread out over a wider frequency band (several thousand hertz).”

3.4. Line 125: Could you explain the reason for choosing 5 dB as DT?
     3.4.1. Authors’ response:

This value was defined based on a previous study conducted in the Salish Sea. We added the following:

L130: DT was set to 5 dB for this analysis, as it was empirically shown to be the signal-to-noise ratio above which automatic killer whale call detectors perform reliably (Mouy et al. 2012).

3.5. Lines 152-156: This description of call types does not belong in the Methods. I recommend moving it to the Introduction, where it would fit as background information (see my comment on lines 64–67).
     3.5.1. Authors’ response:

We have moved the sound description paragraph to the Introduction section (see Author’s response 3.1.1.)

3.6. Fig 2: You should label “SRKW” and “NRKW” directly on top of the spectrogram panels so that we can immediately understand which column corresponds to which group, without needing to refer back to the caption.
     3.6.1. Authors’ response:

The figure has been updated accordingly.

3.7. Line 207: I don’t think the depth of the hydrophones was mentioned earlier in the Methods. It would be helpful to include a brief section/paragraph describing the deployment details of the instruments, including their depth and the time period over which recordings were made.
     3.7.1. Authors’ response:

Information about the hydrophones’ depth has now been added as a “Hydrophone depth” column in Table S3. This same table already includes the time window over which recordings were made. To make this clearer in the manuscript we added the following sentence:

L 268: “Table S3 indicates the time periods selected for each season, as well as the coordinates and depths of the acoustic recorders used.”

3.8. Fig 4:You should specify in the caption that the red lines correspond to the transects.
     3.8.1. Authors’ response:

The caption of Fig.4 was updated accordingly

3.9. Fig 5: You should label “winter” and “summer” directly on top of the plots.
     3.9.1. Authors’ response:

Fig. 5 was updated accordingly

3.10. Fig 6: You should specify blue for summer and orange for winter in the caption.
      3.10.1. Authors’ response:

The caption was updated accordingly

3.11. Fig 9, 10 11: You should add labels directly on the plots to ensure consistency with previous figures and to make the figure easier to interpret without having to refer to the caption each time.
      3.11.1. Authors’ response:

Fig. 9 was updated accordingly. Figures 10 and 11 remain unchanged because their labels include more than just location and season, and we believe that adding longer labels would not improve clarity.

3.12. Lines 400-401: This sentence appears to contradict line 391. How can Mouat Point be listed among the locations with larger detection ranges and also be described as having one of the shortest median detection ranges? Please clarify this point.
      3.12.1. Authors’ response:

We agree that this point was not sufficiently clear. Mouat Point is part of the Southern Gulf Islands but had shorter detection ranges. We have reworded the text as follows:

“Most PAM stations around the Southern Gulf Islands (Sturdies Bay, Enterprise Reef, Tilly Point and East Point) had larger detection ranges (i.e., median detection ranges for P = 0.5, Fig. 6) than stations located in the Strait of Juan de Fuca and on Swiftsure Bank (Sheringham Point, Port Renfrew, Swiftsure Bank).”

3.13. Lines 421-424: The explanation that shorter summer detection ranges may be due to "undersampling" of winter data seems counterintuitive. If winter data are limited, it is unclear how this would explain longer detection ranges in winter than in summer. Please clarify the logic here.
      3.13.1. Authors’ response:

We do not state that “undersampling” systematically leads to shorter detection ranges. Our only caution here is that this location is less well represented than others and may not capture the full variability of possible background noise conditions. The results for this location should therefore be interpreted with caution.

3.14. Line 422: “As already indicated”: this is slightly vague. Perhaps refer back more explicitly to the earlier section where this was discussed.
      3.14.1. Authors’ response:

This point was mentioned in the previous paragraph (L428–429). We removed the phrase “As already indicated” to avoid any potential confusion for the reader.

3.15. Lines 504-512: Please clarify whether the model represents a minimum or maximum expected detection performance and in what context.
      3.15.1. Authors’ response:

To clarify, we added:

L528-529: “…(i.e. increased probability of detecting calling animals from farther away).”

For conciseness and clarity, we also deleted parts of this paragraph that were not necessary and could lead to confusion.

3.16. Line 592: The citation is missing.
      3.16.1. Authors’ response:

Thank you for catching this. Reference 18 is now properly entered.
